# Potential impact of midwives in preventing and reducing maternal and neonatal mortality and stillbirths: a Lives Saved Tool modelling study

**DOI:** 10.1016/S2214-109X(20)30397-1

**Published:** 2020-12-01

**Authors:** Andrea Nove, Ingrid K Friberg, Luc de Bernis, Fran McConville, Allisyn C Moran, Maria Najjemba, Petra ten Hoope-Bender, Sally Tracy, Caroline S E Homer

**Affiliations:** a Novametrics, Duffield, UK; b Tacoma, WA, USA; c Bias, France; d Department of Maternal, Newborn, Child and Adolescent Health and Ageing, World Health Organization, Geneva, Switzerland; e Uganda Country Office, United Nations Population Fund, Kampala, Uganda; f United Nations Population Fund Office of Geneva, Geneva, Switzerland; g Faculty of Medicine and Health, University of Sydney, Sydney, NSW, Australia; h Maternal, Child and Adolescent Health, Burnet Institute, Melbourne, VIC, Australia

## Abstract

**Background:**

Strengthening the capacity of midwives to deliver high-quality maternal and newborn health services has been highlighted as a priority by global health organisations. To support low-income and middle-income countries (LMICs) in their decisions about investments in health, we aimed to estimate the potential impact of midwives on reducing maternal and neonatal deaths and stillbirths under several intervention coverage scenarios.

**Methods:**

For this modelling study, we used the Lives Saved Tool to estimate the number of deaths that would be averted by 2035, if coverage of health interventions that can be delivered by professional midwives were scaled up in 88 countries that account for the vast majority of the world's maternal and neonatal deaths and stillbirths. We used four scenarios to assess the effects of increasing the coverage of midwife-delivered interventions by a modest amount (10% every 5 years), a substantial amount (25% every 5 years), and the amount needed to reach universal coverage of these interventions (ie, to 95%); and the effects of coverage attrition (a 2% decrease every 5 years). We grouped countries in three equal-sized groups according to their Human Development Index. Group A included the 30 countries with the lowest HDI, group B included 29 low-to-medium HDI countries, and group C included 29 medium-to-high HDI countries.

**Findings:**

We estimated that, relative to current coverage, a substantial increase in coverage of midwife-delivered interventions could avert 41% of maternal deaths, 39% of neonatal deaths, and 26% of stillbirths, equating to 2·2 million deaths averted per year by 2035. Even a modest increase in coverage of midwife-delivered interventions could avert 22% of maternal deaths, 23% of neonatal deaths, and 14% of stillbirths, equating to 1·3 million deaths averted per year by 2035. Relative to current coverage, universal coverage of midwife-delivered interventions would avert 67% of maternal deaths, 64% of neonatal deaths, and 65% of stillbirths, allowing 4·3 million lives to be saved annually by 2035. These deaths averted would be particularly concentrated in the group B countries, which currently account for a large proportion of the world's population and have high mortality rates compared with group C.

**Interpretation:**

Midwives can help to substantially reduce maternal and neonatal mortality and stillbirths in LMICs. However, to realise this potential, midwives need to have skills and competencies in line with recommendations from the International Confederation of Midwives, to be part of a team of sufficient size and skill, and to work in an enabling environment. Our study highlights the potential of midwives but there are many challenges to the achievement of this potential. If increased coverage of midwife-delivered interventions can be achieved, health systems will be better able to provide effective coverage of essential sexual, reproductive, maternal, newborn, and adolescent health interventions.

**Funding:**

New Venture Fund.

## Introduction

Improving maternal and newborn health is one of the unfinished agendas of the Millennium Development Goals, and it remains a high priority area in the era of the Sustainable Development Goals. The Global Strategy for Women's, Children's, and Adolescents' Health (2016–2030) also highlights the importance of the health and wellbeing of every woman, child, and adolescent, including access to essential interventions and an effective health workforce.^1^

The awareness of the capacity of midwives to contribute to this global agenda has increased over the past decade. The 2014 *Lancet* Series on Midwifery showed that interventions provided by the midwifery workforce could reduce maternal and newborn deaths and stillbirths in low-income and middle-income countries (LMICs) by 30–80%, depending on the level of intervention coverage.^2^ The Series showed that more efficient use of resources and improved outcomes were achieved when the workforce included enough midwives who were educated, trained, licensed, regulated, and working in an enabling environment.^3^ The 2014 State of the World's Midwifery Report (SoWMy) showed that midwives educated and regulated according to international standards can provide more than 80% of the essential care needed for women and neonates.^4^ In high-income settings, midwife-led continuity of care has been associated with positive outcomes, including fewer preterm births, fewer fetal losses at any gestation, and high rates of positive experiences reported by women.^5^

**Research in context**

**Evidence before this study**

This study draws on the second paper from the *Lancet* Series on Midwifery (2014), the State of the World's Midwifery Report (2014), and we used the Lives Saved Tool (LiST) including 2017 maternal mortality ratio estimates, 2018 neonatal mortality rate estimates, and 2015 stillbirth rate estimates. The modelled interventions were those in the International Confederation of Midwives essential midwifery competencies and the Global Strategy for Women's, Children's and Adolescents' Health. The 2014 *Lancet* Series estimated that universal coverage of midwifery interventions could avert most maternal and neonatal deaths and stillbirths. This estimate included a range of interventions, some deliverable in their entirety by midwives and some requiring input from a wider range of health professionals. No formal literature search was done, as it was not necessary for the aims of our study.

**Added value of this study**

Our study highlights the substantial potential of midwives as a single occupation group to contribute to reducing mortality, while recognising that midwives can only be fully effective as part of a multidisciplinary team operating within an enabling environment. Since the publication of the *Lancet* Series on Midwifery, the evidence base on which LiST is built has been updated and improved, and thus the estimates presented in this study are almost certainly more accurate than those published in 2014. We aggregated the results of 88 individual country projections rather than generating averages across country groupings. Our study also presents important additional analyses relating to the contribution of different types of interventions to mortality reduction, and some limited analyses of outcomes other than maternal and neonatal mortality and stillbirths (eg, number of abortions and exclusive breastfeeding).

**Implications of all the available evidence**

Greater use of midwives by LMICs could substantially improve maternal and newborn survival because interventions that can be delivered in their entirety by midwives are projected to be able to save more lives than many other interventions. However, substantial barriers prevent midwives in these contexts from achieving their full life-saving potential. Investment in midwives needs to include investing not only in their numbers, but also in their education, training, regulation, and working environment.

Strengthening the capacity of midwives to deliver high-quality maternal and newborn health services is a priority for the UN Population Fund (UNFPA)^6^ and WHO.^7^ The International Confederation of Midwives (ICM) also provides leadership in this area, for example by publishing essential competencies for midwifery practice^8^ and global standards for midwifery education.^9^

To support country-level decision making about health system investments, we aimed to estimate the potential impact of midwives on reducing maternal and neonatal mortality and stillbirths, while recognising that midwives are most effective when working within a multidisciplinary team. We estimated the number of lives that could be saved under various scenarios for scaling up coverage of interventions that can be delivered by midwives who are educated, trained, regulated to international standards, and working in an enabling environment in the countries that account for most of the world's maternal and newborn mortality and stillbirths.

## Methods

### Overview

For this modelling study, we used the Lives Saved Tool (LiST), part of the Spectrum software suite, to model the country-specific effect of changes in health intervention coverage on mortality. This approach uses the best available estimates of baseline health status, population size, and linear assumptions of intervention effectiveness on specific causes of death. We used LiST to model the effects on mortality and nutrition that could be attained by scaling up the interventions that can be provided specifically by midwives. We used Spectrum, version 5.8, for all analyses. All LiST default assumptions were used unless otherwise stated, including 2017 maternal mortality ratio estimates,^10^ 2018 neonatal mortality rate estimates,^11^ and 2015 stillbirth rate estimates.^12^

### Health interventions

LiST only includes health interventions that directly affect mortality (maternal, neonatal, child, or stillbirth) or nutritional status. LiST excludes interventions without proven effect on mortality and those that improve other outcomes, such as routine monitoring with a partograph, counselling on birth preparedness, and screening for post-partum depression.^1^ For an intervention to be included in our modelling study, it had to be available within LiST or Spectrum, deliverable in its entirety by a midwife according to ICM global standards (hereafter referred to as midwife-delivered interventions), and listed as an essential intervention within the ICM essential midwifery competencies^8^ or the Global Strategy for Women's, Children's, and Adolescents' Health.^1^ This selection was done by listing the LiST interventions and then mapping the ICM competencies to them. Any areas of uncertainty were resolved by discussion among the study team. The modelled interventions and their baseline coverage values are listed in the appendix (p 1). We should note that the full scope of practice of a midwife is broader than this: midwives play important roles as part of teams doing other life-saving interventions, such as caesarean sections, assisted deliveries, and blood transfusions.

Changes to LiST defaults were made for one intervention: antenatal corticosteroids. The same default coverage was assumed as for uterotonics and the previous default effectiveness^13^ was used. Although antenatal corticosteroids for preterm labour is a standard LiST intervention, coverage and effectiveness currently default to 0, due to updates to WHO guidelines regarding this intervention. However, antenatal corticosteroids are a midwife-delivered intervention, and this analysis assumed that midwives are practising in a strong and supportive health system.

### Scenarios

Our analysis used four scenarios to show the effects of altering the coverage of midwife-delivered interventions by a modest amount, a substantial amount, and by the amount needed to reach universal coverage of these interventions (table 1). The fourth scenario used was an attrition scenario, which indicates the effect of either a small decline in the training, education, and deployment of midwives, or no increase in these to match population growth. These are the same scenarios used in the 2014 *Lancet* Series on Midwifery.

**Table 1:** Scenarios used to model the impact of midwives on maternal and neonatal deaths and stillbirths, 2020–35

	**Description**	**Percentage change in midwife-delivered intervention coverage rates**
0	No scale-up	No change from baseline (2020) coverage rates (constant contraceptive prevalence rate*)
1	Modest scale-up in coverage	10% increase on baseline coverage rates every 5 years up to a maximum of 95%† (coverage of modern contraceptive methods increases by 0·5% per year*)
2	Substantial scale-up in coverage	25% increase on baseline coverage rates every 5 years up to a maximum of 95%† (coverage of modern contraceptive methods increases by 1% per year*)
3	Universal coverage	95% coverage of all interventions by 2035† (coverage of modern contraceptive methods increases by 2% per year*)
4	Attrition	2% decrease every 5 years (coverage of modern contraceptive methods decreases by 0·2% per year)

Baseline coverage rates are presented in the appendix (pp 3–7) and coverage rates achieved under each scenario are also presented in the appendix (pp 8–48).

* The maximum reasonable annual increase for family planning interventions was considered to be 2 percentage points, and therefore different rules were applied to these interventions; all family planning interventions were limited to a level at which the total fertility rate did not fall below 2·1 (the replacement level), except when the default UN trends within Spectrum suggest that a lower rate has been or will be achieved by 2028.

† Coverage rates for all interventions were capped at 95%, except for interventions that had a coverage rate higher than this level at baseline, in which case the model assumed no additional increase.

The analysis included the 81 Countdown to 2030 countries plus the seven Countdown to 2015 countries that are not Countdown to 2030 countries (Brazil, China, Egypt, Mexico, Peru, São Tomé and Príncipe, and Vietnam). Collectively, these 88 countries accounted for 98% of the world's maternal deaths in 2017,^10^ 96% of the world's neonatal deaths in 2018,^11^ and 95% of the world's stillbirths in 2015.^12^ We used the 2018 Human Development Index (HDI)^14^ to classify the countries in three equal-sized groups (appendix p 2). Group A included the 30 countries with the lowest HDI, group B included 29 low-to-medium HDI countries, and group C included 29 medium-to-high HDI countries.

We created individual LiST baseline projections for each of the 88 countries from 2020 to 2035 (appendix pp 3–7). On the basis of these individual country projections, we calculated results for each group of countries by aggregating the individual country estimates. These baseline results were compared with the various scenarios of how coverage of midwife-delivered interventions might change between 2020 and 2035 (table 1).

### Role of the funding source

The funder of the study had no role in study design, data analysis, data interpretation, or writing of this paper. The corresponding author had full access to all the data in the study and had final responsibility for the decision to submit for publication.

## Results

**Figure 1:**
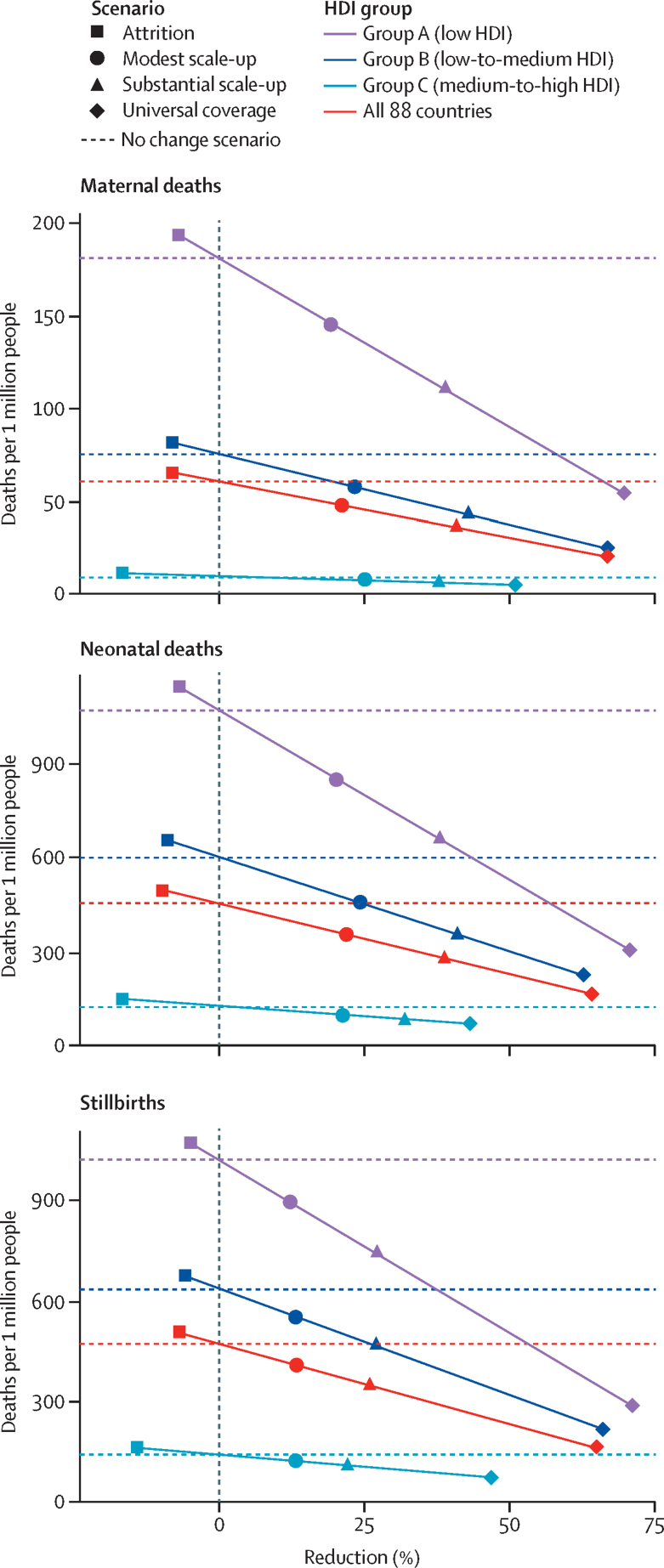
Projected relative reductions in maternal and neonatal deaths and stillbirths per 1 million people in 2035, by country HDI group HDI=Human Development Index. Modest scale-up assumes a 10% increase in coverage every 5 years; substantial scale-up assumes a 25% increase in coverage every 5 years; universal coverage assumes 95% coverage of all interventions by 2035; and attrition assumes a 2% decrease in coverage every 5 years.

A substantial scale-up of midwife-delivered interventions (scenario 2: a 25% increase in coverage every 5 years from 2020 to 2035) in the 88 current and former Countdown countries would result in 41% fewer maternal deaths (20 fewer per million people), 26% fewer stillbirths (100 fewer per million people), and 39% fewer neonatal deaths (150 fewer per million people), relative to a scenario of no change in coverage (table 2, figure 1). Under this scenario, the number of stillbirths and neonatal deaths averted would be proportionally greater in groups A and B (low and low-to-medium HDI) than in group C (medium to high HDI).

**Table 2:** Projected relative reductions in maternal and neonatal deaths and stillbirths per 1 million people in 2035, by country HDI group

	**Scenario 0: no change; deaths (per million)**	**Scenario 1: modest scale-up**		**Scenario 2: substantial scale-up**		**Scenario 3: universal coverage**		**Scenario 4: attrition**	
		Deaths (per million)	Reduction (%)	Deaths (per million)	Reduction (%)	Deaths (per million)	Reduction (%)	Deaths (per million)	Reduction (%)
**Group A: low HDI**									
Maternal deaths	200	150	20%	100	39%	50	70%	200	−7%
Stillbirths	1000	900	13%	750	27%	300	71%	1050	−5%
Neonatal deaths	1050	850	21%	650	38%	300	71%	1150	−7%
**Group B: low-to-medium HDI**									
Maternal deaths	80	60	24%	40	43%	20	67%	80	−8%
Stillbirths	650	550	14%	450	27%	200	66%	700	−6%
Neonatal deaths	600	450	25%	350	41%	200	63%	650	−9%
**Group C: medium-to-high HDI**									
Maternal deaths	10	5	26%	5	38%	5	51%	10	−17%
Stillbirths	150	100	14%	100	22%	50	47%	150	−14%
Neonatal deaths	100	100	22%	80	32%	50	44%	150	−17%
**All 88 countries (weighted average)**									
Maternal deaths	60	50	22%	40	41%	20	67%	70	−8%
Stillbirths	450	400	14%	350	26%	150	65%	500	−7%
Neonatal deaths	450	350	23%	300	39%	150	64%	500	−10%

Numbers of deaths larger than 100 were rounded to the nearest 50, numbers between 11 and 100 were rounded to the nearest ten, and numbers smaller than 10 were rounded to the nearest 5, to reflect the uncertainty due to these being modelled estimates rather than actual data. The percentage reduction calculations were done on the unrounded estimates. Modest scale-up assumes a 10% increase in coverage every 5 years; substantial scale-up assumes a 25% increase in coverage every 5 years; universal coverage assumes 95% coverage of all interventions by 2035; and attrition assumes a 2% decrease in coverage every 5 years. HDI=Human Development Index.

Even a modest scale-up of coverage of midwife-delivered interventions (scenario 1: 10% increase in coverage every 5 years from 2020 to 2035) would result in 22% fewer maternal deaths by 2035 (10 fewer per million), 14% fewer stillbirths (50 fewer per million), and 23% fewer neonatal deaths (100 fewer per million). Proportionally, little difference was observed between groups A, B, and C under this modest scale-up scenario.

Scaling up to universal coverage by 2035 (scenario 3) could result in 67% fewer maternal deaths (40 fewer per million), 65% fewer stillbirths (300 fewer per million), and 64% fewer neonatal deaths (300 fewer per million). The reductions would be proportionally greatest in group A (low HDI, and the furthest from universal coverage), followed by group B and group C.

If universal coverage was achieved, the relative (ie, percentage) reductions would be similar for maternal and neonatal deaths and stillbirths. However, the modest and substantial scale-up scenarios would result in greater relative reductions of maternal and neonatal deaths than of stillbirths, mostly because the baseline coverage of interventions affecting stillbirths is poorer than coverage of interventions affecting maternal and neonatal mortality (appendix pp 3–7).

These proportional mortality reductions can also be considered in terms of absolute numbers of deaths averted (table 3). Achieving substantial scale-up of coverage of midwife-delivered interventions (scenario 2) would avert 2·2 million deaths per year by 2035, whereas universal coverage by 2035 (scenario 3) would avert 4·3 million deaths per year. The absolute number of deaths averted would be greatest in group B partly because this group comprises a larger population than group A (populations in 2035 are projected to be 0·9 billion for group A, 2·9 billion for group B, and 3 billion for group C).^15^ Although group C is projected to have a similarly large population as that of group B, fewer deaths would be averted in this group than in group A or B, because countries in group C already have lower baseline mortality rates (accounting for 9% of the world's maternal deaths, 14% of neonatal deaths, and 17% of stillbirths) than those in groups A and B.

**Table 3:** Projected absolute numbers of maternal and neonatal deaths and stillbirths, and of deaths averted, in 2035, by country HDI group

	**Scenario 0: no change; deaths (thousands)**	**Scenario 1: modestscale-up**		**Scenario 2: substantial scale-up**		**Scenario 3: universal coverage**		**Scenario 4: attrition**	
		Deaths (thousands)	Deaths averted (thousands)	Deaths (thousands)	Deaths averted (thousands)	Deaths (thousands)	Deaths averted (thousands)	Deaths (thousands)	Deaths averted (thousands)
**Group A: low HDI**									
Maternal deaths	168	134	34	103	65	50	118	178	−11
Stillbirths	943	817	126	684	259	269	674	988	−45
Neonatal deaths	982	778	204	604	378	280	702	1050	−68
**Group B: low-to-medium HDI**									
Maternal deaths	220	168	52	126	95	72	148	239	−19
Stillbirths	1863	1597	266	1363	501	638	1225	1981	−118
Neonatal deaths	1757	1324	433	1030	727	647	1110	1920	−164
**Group C: medium-to-high HDI**									
Maternal deaths	28	21	7	17	10	14	14	33	−5
Stillbirths	413	357	56	321	92	219	194	472	−60
Neonatal deaths	364	283	81	247	116	203	161	427	−63
**All 88 countries**									
Maternal deaths	416	323	93	246	170	136	280	450	−34
Stillbirths	3219	2771	448	2368	852	1126	2093	3441	−222
Neonatal deaths	3102	2385	718	1881	1221	1130	1972	3397	−295

HDI=Human Development Index. Modest scale-up assumes a 10% increase in coverage every 5 years; substantial scale-up assumes a 25% increase in coverage every 5 years; universal coverage assumes 95% coverage of all interventions by 2035; and attrition assumes a 2% decrease in coverage every 5 years.

The attrition scenario showed that even a small decrease in coverage rates (such as one that could result from not investing in additional midwives to keep pace with population growth) would result in 551 000 more deaths than if the 88 countries maintained current coverage of midwife-delivered interventions (table 3).

**Figure 2:**
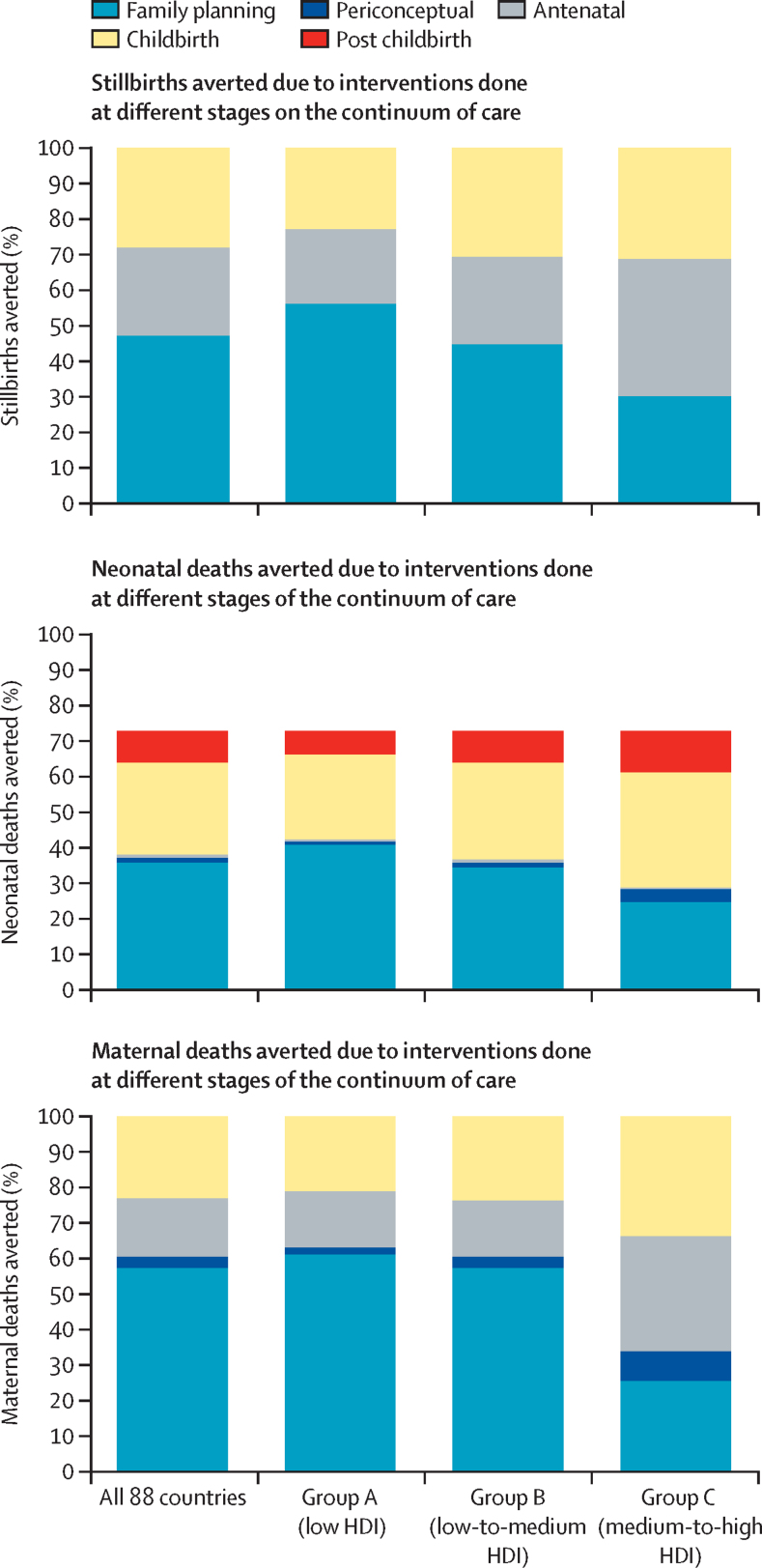
Proportion of deaths averted in the universal coverage scenario in 2035, due to different types of midwife-delivered intervention, by HDI group HDI=Human Development Index.

We assessed the types of interventions projected to make the largest contribution to the lives saved in 2035 under scenario 3 (universal coverage), relative to no change in coverage (scenario 0), and how this varied by HDI group (figure 2). Family planning was projected to account for just under half of the stillbirths and neonatal deaths averted (980 000 [47%] of 2·1 million stillbirths and 960 000 [49%] of 2 million neonatal deaths) and this intervention would avert more than half of the stillbirths and neonatal deaths in group A (low HDI). In group C (medium-to-high HDI), antenatal interventions (especially hypertensive disorder case management) were projected to make the greatest contribution to the reduction in stillbirths, and childbirth and post-childbirth interventions (especially antenatal corticosteroids for preterm labour, assisted vaginal birth, management of preterm babies, and management of neonatal sepsis and pneumonia) were projected to make the greatest contribution to reducing neonatal deaths. None of the stillbirths averted were attributable to periconceptual interventions; current evidence on periconceptual folic acid supplementation does not prove that it reduces stillbirths.^16^

Regarding maternal deaths, midwife-delivered family planning interventions were projected to account for over half of the deaths averted, with the highest proportions in groups A and B (low and low-to-medium HDI; figure 2). In group C (medium-to-high HDI), antenatal and childbirth interventions were projected to account for most of the deaths averted. Almost a quarter of averted deaths would be due to increased coverage of midwife-delivered childbirth interventions (most notably parenteral administration of uterotonics and assisted vaginal delivery). In group C (medium-to-high HDI), 4600 (32%) of 14 000 deaths would be averted by antenatal interventions (most notably screening and management of hypertension and pre-eclampsia and iron folate supplementation).

The LiST model enabled examination of some of the other benefits of increased coverage of midwife-delivered interventions, specifically those related to HIV, abortion, and nutrition. For example, the increase in coverage of midwife-delivered family planning interventions could reduce the number of abortions by approximately a third (down from 40 million to 25 million). Supporting women who are HIV positive to receive prevention of mother-to-child transmission interventions before the index pregnancy could avoid 44% of infant deaths due to HIV (down from 27 000 to 15 000). Similarly, the promotion of breastfeeding could increase the proportion of children aged 1–5 months who are exclusively breastfed from 37% to 55% in the lowest HDI countries, with the attendant benefits to the family of improved bonding and health and cost savings.

## Discussion

Achieving a substantial scale-up of coverage of essential interventions that can be delivered by midwives who are educated, regulated to global standards, and working within an enabling environment by 2035 could avert 40% of maternal and neonatal deaths and 26% of stillbirths, relative to those projected to occur under current coverage. Achieving universal coverage could avert 65% of all these deaths.

Our analysis was designed to replicate and refine that done for the 2014 *Lancet* Series on Midwifery^2^ to update the 2014 projections and to estimate the impact of midwife-delivered interventions (as opposed to all interventions provided by the sexual, reproductive, maternal, newborn, and adolescent health [SRMNAH] workforce). Four key differences exist between the two analyses (appendix p 49). First, the 2014 estimates were based on 78 countries, whereas the 2020 estimates are based on 88 countries. Second, the 2014 estimates were produced by generating average mortality rates, intervention coverage values, HIV prevalence rates, and total fertility rates for country groups and analysing these rates on a standard starting population, whereas the 2020 estimates were produced by making separate projections for each country and aggregating the results. Third, the 2020 analysis excluded interventions included in 2014 that require specialist care: fetal growth restriction detection and management, comprehensive emergency obstetric care (blood transfusion and surgery), and hospital-based care for neonates. Fourth, the 2020 analysis excluded interventions that the authors felt were not core interventions provided by a midwife, on the basis of their interpretation of the ICM guidelines, specifically calcium supplementation in pregnancy and balanced energy supplementation in pregnancy (ie, food supplementation). However, safe abortion, ectopic pregnancy management, management of diabetes in pregnancy, antenatal corticosteroids, and induction of labour for pregnancies lasting 41 weeks or longer were all considered to be interventions that can and should be provided by midwives.

The 2014 *Lancet* Series on Midwifery concluded that universal coverage of SRMNAH (not just midwife-delivered) interventions would avert about 80% of maternal and neonatal deaths and stillbirths, and that midwives would avert about 70% of these deaths. Two main reasons exist for the slightly lower estimate of 65% in our analysis. First, the baseline coverage rates for several interventions were higher (ie, most countries were closer to universal coverage at baseline) than those in the *Lancet* Series, due to both improved coverage and changes in proxy estimates based on better data. Second, several of the effectiveness estimates in LiST (eg, folic acid supplementation) are lower than those in 2014 because of an updated evidence base. This difference had the effect of reducing the modelled estimates of deaths averted by specific interventions. Additionally, the baseline mortality and fertility rates for 2020 were lower than those in the earlier analysis, resulting in fewer deaths to avert in the present day (appendix p 2).

The 2014 *Lancet* Series concluded that the impact of universal coverage on stillbirths would be lower than that on maternal and neonatal mortality, whereas our analysis found that universal coverage would result in similar reductions for all three types of mortality. This different conclusion is due mainly to one intervention: assisted vaginal delivery, which makes a major contribution to reducing stillbirth. The baseline coverage rates of this intervention in LiST are much lower than in the 2014 dataset: most countries are further from universal coverage than was previously thought, so there is greater scope to reduce mortality.

As in any modelled estimates, our analysis has some limitations. Midwives provide a much wider range of interventions than that included in the model, and the model includes assumptions that do not hold true in every setting. Therefore, the results are indicative and directional rather than exact estimates. The relevance of these estimates in countries where midwifery is not an established profession might be questionable because these interventions will be delivered by other health professionals. However, the results remain relevant to all LMICs because the interventions should be delivered by other SRMNAH workers, and the many lives that could be saved indicates that investing in midwives is a cost-effective approach to improving maternal and newborn health outcomes.

In addition to saving lives, reviews have shown that continuity of midwifery care is associated with improved outcomes, but the reasons for this are not fully understood.^7^ The geographical and social proximity of midwives to the communities they serve is a strength^17^ and facilitates integrated, people-centred care. Another possible contributory factor is that many midwife-delivered interventions focus on prevention of pathology (eg, antenatal care, birth preparedness, promotion of breastfeeding).^7^ Much of the evidence for the effect of midwives on outcomes other than mortality comes from high-income countries, but some LMICs (eg, Afghanistan, Bangladesh, Burkina Faso, Cambodia, Indonesia, and Morocco) are deploying midwives as a core element of their SRMNAH strategy,^18^ thus more evidence from LMICs should become available in the near future.^19^

Barriers to enabling and supporting midwives in LMICs are numerous: insufficient numbers of qualified midwives and inequitable distribution, poor transport links, the cost of accessing care, scarcity of supplies and equipment, inadequate education and regulation, and, in some countries, lack of trust among the public due to previous experiences of disrespectful care.^20^ For midwives, barriers to providing high-quality care include social factors (eg, gender inequality and exposure to violence),^21^, ^22^ professional factors (eg, gender issues,^23^ absence of midwives in policy dialogue, low recognition by other professions of midwifery skills, restrictions on practice, poor education, and scarcity of supplies and equipment), and economic factors (eg, low or irregular salaries and poor housing and transport infrastructure).^24^

Insufficient human and other resources and the inequitable distribution of resources reduce the effect of midwives on health outcomes.^20^ Countries need to accurately estimate their health workforce needs. Some use workforce-to-population ratios for this purpose (eg, a number of doctors, nurses, and midwives per 10 000 population). Although simple to calculate and easy to communicate, this method is not sensitive to geographical variations, is usually based on simple headcounts that do not always accurately reflect the health system context and variability in health services delivery, and does not specify the type and skills of health workers required nor options for configuring teams of health workers with the appropriate skill mix. In the past few years, efforts to develop methods for estimating SRMNAH workforce requirements have shown that such needs are strongly influenced by the demography and epidemiology of the population being served.^23^ The capacity to apply such methods remains low in many LMICs. SoWMy 2021 and previous reports in the SoWMy series aim to support countries in this regard.

In many countries, no clear professional distinction exists between midwives and nurses.^4^ This can lead to the unique contribution of midwives being overlooked by their colleagues and the public, constraining their impact. Establishing a positive reputation and respectful relationships with other occupation groups will take time.^25^ The gender dimension should be considered: most midwives are women, and most senior physicians and health service managers are men.^21^ The struggle of midwives for recognition as skilled, autonomous professionals is not only a barrier to career progression, but also a disincentive for people to consider a career as a midwife.^4^, ^24^

SoWMy 2014 highlighted gaps in the quality of midwife education in LMICs due to insufficient teaching staff, infrastructure, and opportunities for clinical practice during pre-service education.^4^ A 2019 review of education and training for maternal and newborn health workers in LMICs found very little high-quality evidence about the effectiveness of education and training in these settings and noted that evaluations tended to focus on individual clinical skills rather than the full scope of midwifery services.^26^ WHO has published a global framework for action that takes a strategic and comprehensive approach to improving midwifery education,^7^ and international standards and resources for midwifery education have been developed by ICM and WHO.^8^, ^9^, ^27^

In addition to education, midwives need an enabling work environment to maximise their impact. For example, poor water and sanitation at health facilities and education institutions affects midwives' ability to provide a clean, safe birthing environment.^4^ Poor working conditions or a negative organisational culture might foster disrespect and abuse towards service users.^28^ Such issues might be more common among midwives working in remote areas. The first step towards improvement is to gain local recognition that things need to change. Strong and responsive leadership is needed at both facility and country level^22^ to promote the concepts underpinning the decent work agenda, which include social protection and rights at work.

In humanitarian and fragile settings, these issues might be amplified, with additional challenges such as threats to personal security. Such settings merit special attention, not least because they account for 60% of preventable maternal deaths and 45% of neonatal deaths globally.^29^ There is alignment between a midwife's scope of practice and the objectives of the international response to SRMNAH in humanitarian settings.^30^ Midwives are more likely than other SRMNAH workers to remain in post throughout a humanitarian crisis.^31^ Resilience during crises could be improved by ensuring that midwives are enabled and empowered to operate to their full scope of practice and by ensuring public recognition of the interventions they are qualified to provide.^31^ Gaps exist between global guidance on midwives' scope of practice and global guidelines on the role of the SRMNAH workforce in crises, especially regarding the role of midwives in health education, preventive care, and mobilising communities to monitor hazards, especially during the recovery phase.^30^ These gaps might reduce the impact that midwives can have in humanitarian settings.

Even when the positive impact of midwives on SRMNAH outcomes is recognised, countries can be reluctant to invest in them, seeing health workers as a cost rather than an investment. Discussion of the financing and infrastructure implications of scaling up midwifery is important but beyond the scope of our study. There is, however, increasing evidence that investment in health workers not only improves health, but also has multiplier effects on the broader economy.^32^

Midwives can help to achieve substantial reductions in maternal and neonatal mortality and stillbirths in LMICs. Family planning interventions that can be delivered by midwives have the largest impact, but periconceptual, antenatal, childbirth, and postnatal midwife-delivered interventions also make a substantial contribution. To realise this potential, midwives need to have sufficient skills and competencies, be part of a team of sufficient size and skill, and work in an enabling environment. Our analysis highlights the potential of midwives but, in addition to this type of modelling exercise, it is essential to review systematically evidence on midwife-delivered interventions to strengthen the evidence base and promote appropriate investment. If increased coverage of midwife-delivered interventions can be achieved, health systems will be better able to provide effective coverage of essential SRMNAH interventions.
