# Supplementary material for: Potential impact of midwives in preventing and reducing maternal and neonatal mortality and stillbirths: a Lives Saved Tool modelling study
Source: Lancet Glob Health. 2020 Dec 1;9(1):e24–32. doi: 10.1016/S2214-109X(20)30397-1 (PMC7758876; doi:10.1016/S2214-109X(20)30397-1)
Supplement: Supplementary appendix [file mmc1.pdf]

THE LANCET
Global Health

Supplementary appendix

This appendix formed part of the original submission and has been peer reviewed. We post it as supplied by the authors.

Supplement to: Nove A, Friberg IK, de Bernis L, et al. Potential impact of midwives in preventing and reducing maternal and neonatal mortality and stillbirths: a Lives Saved Tool modelling study. *Lancet Glob Health* 2020; published online Dec 1. http://dx.doi.org/10.1016/S2214-109X(20)30397-1.

WebAppendix

A Methodology

Table A1. Midwife-delivered interventions modelled, and proxies used for estimating baseline coverage

	Indicator or proxy indicator and translation formula if no standard indicator is available
**Before conception (family planning)**	
Modern methods of contraception	Percentage of women at risk of getting pregnant currently using any modern method of contraception
**Around the time of conception**	
Folic acid supplementation	Proxy formula: 5% of women who have ANC4+ receive folic acid
Ectopic pregnancy care management	Proxy formula: if facility delivery is >50%, 0.75 × facility delivery; if facility delivery is 30–50%, 0.50 × facility delivery; if facility delivery is <30%, 0.10 × facility delivery
Safe abortion services	Percentage of women receiving an abortion who have a safe abortion (i.e. medical, surgical)
Post abortion care	Proxy formula: if facility delivery is >50%, 0.75 × facility delivery; if facility delivery is 30–50%, 0.50 × facility delivery; if facility delivery is <30%, 0.10 × facility delivery
**After conception (antenatal care)**	
Tetanus toxoid	Protected by tetanus toxoid at birth
Intermittent preventive treatment in pregnancy	Percentage of pregnant women protected against malaria with two or more doses of sulfadoxine/pyrimethamine or other options if appropriate
Syphilis detection and treatment, if needed	Proxy formula: -1.22 + 3.36 (blood sample) + -5.23 (early ANC)* [14]
Iron folate supplementation	Percentage of pregnant women receiving ≥90 days of iron folate
Hypertension screening and management	Proxy formula: -1.62 + 2.5 (urine sample)*
Diabetes case management	Proxy formula: -3.21 + 2.61 (blood sample)*
Malaria case management	Proxy formula: -1.64 + 2.92 (blood sample)*
Screening for and management of pre-eclampsia with magnesium sulphate	Proxy formula: -6.44 + 4.91 (blood sample) + 2.56 (ANC4+)*
Prevention of mother-to-child transmission of HIV	Percentage of HIV positive pregnant women starting on option B+ prior to conception
**During labour and birth**	
Clean birth environment	Automated LiST formula**
Immediate drying and additional stimulation	Automated LiST formula**
Thermal protection	Automated LiST formula**
Clean cord care	Automated LiST formula**
Manual removal of placenta	Automated LiST formula**
Parenteral administration of anti-convulsants	Automated LiST formula**
Antibiotics for preterm or prolonged PRoM	Automated LiST formula**
Parenteral administration of antibiotics	Automated LiST formula**
Assisted vaginal delivery	Automated LiST formula**
Neonatal resuscitation	Automated LiST formula**
Parenteral administration of uterotonics	Automated LiST formula**
Removal of retained products of conception	Automated LiST formula**
Induction of labour for pregnancies lasting 41+ weeks	Automated LiST formula**
Antenatal corticosteroids for preterm labour	Used LiST formula values** for parenteral administration of anti-convulsants
**Post-partum and newborn care**	
Breastfeeding promotion	Percentage of children 1-5 months of age exclusively breastfed
Kangaroo mother care	Proxy: 5% of health facility deliveries
Injectable antibiotics for neonatal sepsis	Proxy: 100% of health facility deliveries

See the LiST manual and help menus for additional information on default sources.[a] ANC4+=attended 4 or more antenatal care visits. PRoM=premature rupture of membranes. *These formulae are automatically calculated within LiST, utilising information on the proportion of women giving a urine sample or blood sample during pregnancy, starting ANC before 4 months (early ANC), or attending 4+ ANC visits (ANC4+). **Automatically calculated within LiST and based on Kanyangarara et al.[b]

Table A2. Countries included in the modelling

Group	Countries
Group A: low HDI	Afghanistan, Benin, Burkina Faso, Burundi, Central African Republic, Chad, Côte d'Ivoire, Democratic Republic of the Congo, Djibouti, Eritrea, Ethiopia, Gambia, Guinea, Guinea-Bissau, Haiti, Lesotho, Liberia, Madagascar, Malawi, Mali, Mauritania, Mozambique, Niger, Senegal, Sierra Leone, Somalia*, South Sudan, Sudan, Togo, Yemen (30 countries)
Group B: low-to-medium HDI	Angola, Bangladesh, Bhutan, Cambodia, Cameroon, Comoros, Congo, Equatorial Guinea, Eswatini, Ghana, Guatemala, Honduras, India, Kenya, Lao People's Democratic Republic, Myanmar, Namibia, Nepal, Nigeria, Pakistan, Papua New Guinea, Rwanda, São Tomé and Principe, Solomon Islands, Timor-Leste, Uganda, United Republic of Tanzania, Zambia, Zimbabwe (29 countries).
Group C: medium-to-high HDI	Algeria, Azerbaijan, Bolivia, Botswana, Brazil, China, Democratic People's Republic of Korea**, Dominican Republic, Egypt, Gabon, Guyana, Indonesia, Iraq, Jamaica, Kyrgyzstan, Mexico, Morocco, Nicaragua, Panama, Paraguay, Peru, Philippines, South Africa, Suriname, Tajikistan, Turkmenistan, Uzbekistan, Venezuela, Viet Nam (29 countries).

HDI = Human Development Index. *Somalia was moved from its 'other country' HDI category into Group A.
**DPR Korea was moved from its 'other country' HDI category into Group C.
Countries coloured brown were not included in Homer et al (2014).[c]
Countries coloured blue were included in Homer et al (2014) but were in a different HDI group.
Countries underlined are former Countdown countries.

Table A3: Comparison of baseline values for key measures of mortality and fertility, by HDI classification group*

	HDI group	2014 baseline	2020 baseline	% change
Maternal mortality ratio	A	573	556	-3%
	B	312	270	-13%
	C	154	90	-42%
Neonatal mortality rate	A	36	29	-20%
	B	29	25	-13%
	C	19	8	-60%
Total fertility rate	A	5.3	4.7	-11%
	B	4.3	2.8	-34%
	C	3.0	2.0	-33%

HDI=Human Development Index. * The countries within each HDI grouping were not exactly the same in 2014 as in 2020 (see Table A2).

[a] Johns Hopkins Bloomberg School of Public Health. The Lives Saved Tool Manuals. 2020. https://www.livessavedtool.org/manuals (accessed Feb 26, 2020)
[b] Kanyangarara M, Chou VB, Creanga AA, Walker N. Linking household and health facility surveys to assess obstetric service availability, readiness and coverage: Evidence from 17 low- and middle-income countries. *J Glob Health* 2018; **8**: 010603
[c] Homer CSE, Friberg IK, Dias MAB, *et al.* The projected effect of scaling up midwifery. *Lancet* 2014; **384**: 1146–57

B Baseline coverage values

The three tables below show all of the baseline coverage data from the Lives Saved Tool used for the analysis.

Table B1. Baseline coverage of periconceptual and antenatal interventions, 88 countries (%)

	Periconceptual Interventions				Antenatal Interventions							
Country	Folic acid suppl./ fortification	Safe abortion services	Post abortion case mgmt	Ectopic pregnancy case mgmt	Tetanus toxoid	Syphilis detection/ treatment	IPTp	Iron suppl.	Diabetes case mgmt	Hyper-tensive disorder case mgmt	Malaria case mgmt	MgSO4
Afghanistan	0.9	35.1	24.2	24.2	70	14.6	0	6.8	3.3	4.3	13.8	8.5
Algeria	3.4	2.2	72.5	72.5	98	22.9	0	0	12.6	16.2	52.1	32
Angola	3.0	0	22.8	22.8	78	20	38.4	32.1	11.3	14.5	46.7	28.6
Azerbaijan	2.5	40	58.3	58.3	0	19.6	0	1.6	9.2	11.8	38.1	23.4
Bangladesh	1.6	35.1	18.8	18.8	98	6.2	0	0	10.6	7.5	24.2	6.8
Benin	2.6	0	62.9	62.9	85	20.9	33.4	28.6	7.1	12.1	42.7	28.5
Bhutan	3.9	35.1	47.3	47.3	89	24	0	0	14.5	18.6	59.9	36.8
Bolivia	3.6	0.2	50.6	50.6	87	22.3	0	25.1	13.4	17.2	55.4	34
Botswana	3.7	42.4	70.4	70.4	93	18.1	0	0	13.7	17.6	56.8	34.8
Brazil	4.6	0.2	73.6	73.6	94	22.5	0	0	17	21.8	70.5	43.2
Burkina Faso	1.7	0	49.7	49.7	92	8.4	47.6	50.2	0.7	6.4	22.6	6.9
Burundi	2.5	3.3	62.9	62.9	87	24.5	20.7	1.4	9.5	12.2	39.3	24.1
Cambodia	3.8	39	62.4	62.4	93	23.7	0	75.5	14.2	18.2	58.7	36
Cameroon	2.9	0	46.0	46.0	85	20.5	44.3	54.1	11	14.1	45.6	28
Central African Republic	1.9	0	39.1	39.1	60	16.9	26.4	0	7.1	9.2	29.6	18.1
Chad	1.6	0	2.2	2.2	78	13.7	18.1	11	5.9	7.5	24.3	14.9
China	0.0	100	74.4	74.4	0	0	0	0	0	0	0	0
Comoros	2.5	3.3	57.1	57.1	85	22.8	30.8	12.6	9.2	11.8	38.2	23.4
Congo	4.0	0	68.6	68.6	85	23	23.2	42.9	14.8	19	61.2	37.5
Côte d'Ivoire	2.6	0	52.4	52.4	85	23	43.8	25	9.6	12.3	39.7	24.4
DPR Korea	4.7	100	69.2	69.2	98	24.4	0	0	17.6	22.5	72.6	44.5
DR Congo	2.4	0	59.9	59.9	85	34.9	15	4.7	4.7	4.6	31.9	6.6
Djibouti	1.1	3.3	65.6	65.6	98	5.6	0	0	4.2	5.4	17.5	10.7
Dominican Republic	4.6	53.7	73.4	73.4	99	24.2	0	81.5	17.4	22.3	72	44.2
Egypt	4.2	2.2	65.0	65.0	86	22.6	0	36.1	15.7	20.1	65	39.8
Equatorial Guinea	3.3	0	50.5	50.5	70	21.9	27.6	8.7	12.5	16.1	51.9	31.8
Eritrea	2.9	3.3	16.8	16.8	99	17.5	0	0	10.8	13.8	44.5	27.3
Eswatini	3.8	42.5	65.8	65.8	88	24.4	0.1	33.6	14.3	18.3	59	36.2
Ethiopia	1.6	3.3	2.6	2.6	93	15.7	1.2	5.1	6.1	7.9	25.4	15.6
Gabon	3.9	0	67.7	67.7	85	23.4	13.1	56.8	14.5	18.5	59.8	36.7
Gambia	3.8	0	43.4	43.4	92	24.4	63	44.6	14.4	18.4	59.4	36.5
Ghana	4.5	0	59.0	59.0	89	24.1	78	59.4	16.7	21.5	69.2	42.4
Guatemala	4.3	0.6	48.8	48.8	90	22.6	0	29.3	16.1	20.6	66.6	40.8
Guinea	2.5	0	43.1	43.1	80	17.2	42.6	41.5	9.4	12.1	38.9	23.9
Guinea-Bissau	3.2	0	22.0	22.0	83	22.9	45.5	0	12.2	15.6	50.3	30.8
Guyana	4.3	0.2	69.3	69.3	99	22.4	0.1	34.2	16.2	20.8	67.2	41.2
Haiti	3.2	53.7	19.7	19.7	81	19.4	0.2	43.2	15	15.5	32.8	17.1
Honduras	4.4	0.6	62.0	62.0	99	23.9	0	37.4	16.6	21.2	68.5	42
India	2.5	35.1	59.2	59.2	90	19.7	0	38.8	9.5	12.2	39.3	24.1
Indonesia	4.4	39	47.7	47.7	90	23.7	0	32.9	16.4	21	67.7	41.5
Iraq	3.4	40	65.0	65.0	75	21.7	0	0	12.7	16.3	52.6	32.3
Jamaica	4.3	53.7	74.0	74.0	90	24.1	0	0	16	20.6	66.3	40.7
Kenya	2.8	3.3	46.1	46.1	88	52.9	34.7	7.5	12.5	14	32.7	24.6
Kyrgyzstan	4.7	35.1	74.7	74.7	0	24.7	0	2.3	17.7	22.7	73.1	44.8
Lao PDR	3.1	39	48.3	48.3	90	20.1	3.1	25.2	11.7	14.9	48.2	29.6
Lesotho	3.7	42.5	57.4	57.4	85	23.5	0	51.4	13.7	17.6	56.8	34.8
Liberia	3.9	0	41.9	41.9	89	23.7	54.5	21.2	14.5	18.6	60.1	36.9
Madagascar	2.4	3.3	19.0	19.0	78	12.6	22.3	7.6	8.8	11.3	36.6	22.4
Malawi	2.5	3.3	68.0	68.0	89	23.5	76.1	33.4	6.4	5	41	40.6
Mali	1.9	0	21.9	21.9	85	10.4	31.1	18.3	7.1	9.1	29.5	18.1
Mauritania	3.2	0	52.0	52.0	80	21.5	25.2	6.1	11.8	15.1	48.8	29.9
Mexico	4.7	0.6	72.7	72.7	96	24.3	0	0	17.6	22.6	72.9	44.7

Morocco	1.5	2.2	54.5	54.5	88	16.7	0	5.2	5.6	7.2	23.3	14.3
Mozambique	2.7	3.3	48.6	48.6	86	22.4	35.8	25.9	10.1	12.9	41.7	25.6
Myanmar	3.0	39	18.6	18.6	90	20.5	0	59.3	11.2	14.3	46.1	28.3
Namibia	3.1	42.5	65.8	65.8	88	92.6	5.1	38.6	32.2	42.1	45.5	31.8
Nepal	3.5	35.1	43.1	43.1	89	8.2	0	70.9	8.5	15.2	21.8	41.6
Nicaragua	4.4	0.6	56.4	56.4	90	21.7	0	61.6	16.4	21.1	68.1	41.7
Niger	1.7	0	3.0	3.0	81	20.8	36.9	28.6	6.2	8	25.8	15.8
Nigeria	2.5	0	18.8	18.8	55	16.3	21.3	20.5	9.2	11.8	38.1	23.3
Pakistan	2.6	35.1	49.7	49.7	85	21.6	0	21.4	9.6	12.3	39.8	24.4
Panama	4.4	0.6	68.4	68.4	0	23.1	0	0	16.5	21.1	68.1	41.8
Papua New Guinea	2.7	85	38.9	38.9	70	18.3	0	0	10.3	13.2	42.6	26.1
Paraguay	4.7	0.2	69.9	69.9	95	24.4	0	0	17.5	22.5	72.5	44.5
Peru	4.8	0.2	68.0	68.0	95	24.1	0	59.7	17.9	23	74.2	45.5
Philippines	4.3	39	58.3	58.3	90	23.1	0	50.6	16.2	20.7	66.8	41
Rwanda	2.2	3.3	68.0	68.0	90	52	17.7	3.4	8.3	1.9	34.4	7.7
São Tomé and Príncipe	4.2	0	68.3	68.3	99	24.1	56.9	53.5	15.7	20.1	64.8	39.8
Senegal	2.8	0	52.4	52.4	95	24	62.6	63.1	10.5	13.4	43.4	26.6
Sierra Leone	3.9	0	57.5	57.5	90	18.2	68.7	30	11.6	41.4	71	68.1
Solomon Islands	3.2	85	63.4	63.4	85	19.8	1.2	0	12.1	15.5	50.1	30.7
Somalia	0.1	3.3	0.9	0.9	67	6.5	0.9	0	0.4	0.5	1.5	0.9
South Africa	3.8	42.5	71.9	71.9	90	22.5	0	50.5	14.1	18.1	58.5	35.9
South Sudan	0.9	2.2	1.2	1.2	68	10	10.2	0	3.2	4.2	13.4	8.2
Sudan	2.5	2.2	2.8	2.8	80	19.6	1.9	0	9.5	12.2	39.3	24.1
Suriname	3.3	0.2	69.2	69.2	93	23.5	0	0	12.5	16	51.8	31.7
Tajikistan	3.2	35.1	66.2	66.2	0	22.7	0	0.9	12	15.4	49.8	30.5
Timor-Leste	3.8	39	24.3	24.3	83	20.9	0	12.9	14.4	18.5	59.6	36.5
Togo	2.8	0	46.1	46.1	83	18.4	68.2	37.1	18.3	16.7	49.1	9.5
Turkmen-istan	4.8	35.1	74.6	74.6	0	24.7	0	0	18	23.1	74.6	45.7
Uganda	3.0	3.3	55.1	55.1	85	28.1	45.9	22.6	10.1	17.7	40.6	23.1
Tanzania	2.5	3.3	47.0	47.0	90	56.2	56.1	21.4	9.2	11.8	42.3	34.8
Uzbekistan	3.9	35.1	73.0	73.0	0	24.5	0	0	14.7	18.8	60.7	37.2
Venezuela	0.0	0.2	71.3	71.3	70	0	0	0	0	0	0	0
Viet Nam	3.7	39	70.2	70.2	94	23.7	0.5	0	13.8	17.7	57.1	35
Yemen	1.2	40	15.4	15.4	70	14.7	0	5.5	4.6	5.9	19.1	11.7
Zambia	2.7	3.3	50.3	50.3	85	23.6	81.3	59.1	10.1	13	42	25.7
Zimbabwe	3.7	3.3	57.8	57.8	87	77.8	12.1	39.7	28.8	29.7	70.8	71.9

TT: Tetanus toxoid; MgSO4: MgSO4 for management of pre-eclampsia; IPTP: intermittent preventive treatment of malaria in pregnancy

Table B2. Baseline coverage of childbirth care interventions, 88 countries (%)

Country	Clean birth env.	Immed-iate drying & stimul-ation	Therm-al protect-ion	Clean cord care	MROP	Anti-biotics for pPROM	Assist-ed vaginal delivery	Neo-natal resus	Parent-eral admin. of anti-biotics	Parenteral admin. of anti-convul-sants	Parent-eral admin. of utero-tonics	Rem-oval of RPCs	Ante-natal cortico-steroids	Labour induction for preg-nancies 41+ weeks
Afghanistan	39.6	44.2	47.7	46.1	18.1	36.1	12.2	26.6	36.1	34.5	43.2	16.0	34.5	0.9
Algeria	79.2	88.5	95.5	92.2	36.1	72.3	24.4	53.1	72.3	69.1	86.3	32.0	69.1	1.7
Angola	37.4	41.8	45.1	43.5	17.1	34.1	11.5	25.1	34.1	32.6	40.8	15.1	32.6	0.8
Azerbaijan	63.7	71.1	76.8	74.1	29.0	58.1	19.6	42.7	58.1	55.5	69.4	25.7	55.5	1.4
Bangladesh	26.6	27.7	36.2	35.9	8.6	16.8	18.6	28.2	16.8	26.7	29.6	21.3	26.7	7.2
Benin	81.4	83.3	82.9	81.4	69.2	74.0	25.8	46.1	74.0	47.4	81.4	53.6	47.4	1.7
Bhutan	51.8	57.8	62.4	60.2	23.6	47.2	16.0	34.7	47.2	45.1	56.4	20.9	45.1	1.1
Bolivia	55.4	61.8	66.7	64.4	25.2	50.5	17.1	37.1	50.5	48.3	60.3	22.4	48.3	1.2
Botswana	76.9	85.9	92.7	89.5	35.1	70.2	23.7	51.6	70.2	67.1	83.8	31.1	67.1	1.7
Brazil	80.5	89.8	97.0	93.6	36.7	73.4	24.8	53.9	73.4	70.1	87.7	32.5	70.1	1.7
Burkina Faso	64.0	63.9	65.6	65.5	49.1	52.6	7.0	13.0	52.6	15.9	64.1	11.1	15.9	0.9
Burundi	68.8	76.9	83.0	80.1	31.4	62.8	21.2	46.2	62.8	60.0	75.0	27.8	60.0	1.5
Cambodia	68.3	76.2	82.3	79.4	31.1	62.3	21.0	45.8	62.3	59.5	74.4	27.6	59.5	1.5
Cameroon	50.2	56.1	60.5	58.5	22.9	45.8	15.5	33.7	45.8	43.8	54.7	20.3	43.8	1.1
Central African Republic	42.7	47.7	51.5	49.7	19.5	39.0	13.2	28.6	39.0	37.2	46.5	17.3	37.2	0.9
Chad	17.9	20.0	21.6	20.9	8.2	16.4	5.5	12.0	16.4	15.6	19.5	7.2	15.6	0.4
China	81.4	90.8	98.0	94.7	37.1	74.2	25.1	54.5	74.2	70.9	88.6	32.9	70.9	1.7
Comoros	62.4	69.7	75.3	72.7	28.5	57.0	19.2	41.9	57.0	54.4	68.0	25.2	54.4	1.3

Country	Clean birth env.	Immediate drying & stimulation	Thermal protection	Clean cord care	MROP	Antibiotics for pPROM	Assisted vaginal delivery	Neonatal resus	Parenteral admin. of antibiotics	Parenteral admin. of anticonvulsants	Parenteral admin. of uterotonics	Removal of RPCs	Antenatal corticosteroids	Labour induction for pregnancies 41+ weeks
Congo	75.0	83.8	90.4	87.3	34.2	68.5	23.1	50.3	68.5	65.4	81.8	30.3	65.4	1.6
Côte d'Ivoire	57.2	63.9	69.0	66.6	26.1	52.2	17.6	38.4	52.2	49.9	62.4	23.1	49.9	1.2
DPR Korea	75.6	84.4	91.1	88.0	34.5	69.0	23.3	50.7	69.0	65.9	82.4	30.6	65.9	1.6
DR Congo	40.9	63.2	78.9	66.6	21.2	12.2	8.9	4.4	12.2	14.2	61.3	19.9	14.2	1.2
Djibouti	71.7	80.0	86.4	83.4	32.7	65.4	22.1	48.1	65.4	62.5	78.1	29.0	62.5	1.5
Dominican Republic	80.3	89.6	96.7	93.4	36.6	73.2	24.7	53.8	73.2	70.0	87.5	32.4	70.0	1.7
Egypt	71.1	79.4	85.7	82.8	32.4	64.9	21.9	47.7	64.9	62.0	77.5	28.7	62.0	1.5
Equatorial Guinea	55.2	61.6	66.5	64.2	25.2	50.4	17.0	37.0	50.4	48.1	60.1	22.3	48.1	1.2
Eritrea	27.6	30.8	33.2	32.1	12.6	25.1	8.5	18.5	25.1	24.0	30.0	11.1	24.0	0.6
Eswatini	71.9	80.3	86.7	83.7	32.8	65.6	22.2	48.2	65.6	62.7	78.4	29.1	62.7	1.5
Ethiopia	21.5	24.0	25.9	25.0	9.8	19.6	6.6	14.4	19.6	18.8	23.4	8.7	18.8	0.5
Gabon	74.0	82.6	89.2	86.1	33.7	67.5	22.8	49.6	67.5	64.5	80.6	29.9	64.5	1.6
Gambia	47.4	52.9	57.2	55.2	21.6	43.3	14.6	31.8	43.3	41.4	51.7	19.2	41.4	1.0
Ghana	64.5	72.1	77.8	75.1	29.4	58.9	19.9	43.3	58.9	56.3	70.3	26.1	56.3	1.4
Guatemala	53.3	59.5	64.2	62.0	24.3	48.6	16.4	35.7	48.6	46.5	58.1	21.5	46.5	1.1
Guinea	47.1	52.6	56.8	54.8	21.5	43.0	14.5	31.6	43.0	41.1	51.4	19.0	41.1	1.0
Guinea-Bissau	36.1	40.3	43.5	42.0	16.4	32.9	11.1	24.2	32.9	31.4	39.3	14.6	31.4	0.8
Guyana	75.8	84.6	91.4	88.2	34.6	69.2	23.4	50.8	69.2	66.1	82.6	30.6	66.1	1.6
Haiti	33.8	32.6	37.8	38.2	14.8	21.4	7.7	14.9	21.4	28.2	25.5	11.0	28.2	0.6
Honduras	67.9	75.8	81.8	79.0	30.9	61.9	20.9	45.5	61.9	59.2	73.9	27.4	59.2	1.5
India	64.7	72.3	78.0	75.3	29.5	59.1	20.0	43.4	59.1	56.4	70.5	26.2	56.4	1.4
Indonesia	52.2	58.2	62.9	60.7	23.8	47.6	16.1	35.0	47.6	45.5	56.8	21.1	45.5	1.1
Iraq	71.0	79.3	85.6	82.6	32.4	64.8	21.9	47.6	64.8	61.9	77.4	28.7	61.9	1.5
Jamaica	80.8	90.2	97.4	94.0	36.9	73.8	24.9	54.2	73.8	70.5	88.1	32.7	70.5	1.7
Kenya	50.4	53.6	60.7	58.7	42.8	47.0	5.2	50.9	47.0	60.0	46.6	31.3	60.0	1.1
Kyrgyzstan	81.7	91.2	98.4	95.0	37.3	74.5	25.2	54.8	74.5	71.2	89.0	33.0	71.2	1.8
Lao PDR	52.8	59.0	63.7	61.5	24.1	48.2	16.3	35.4	48.2	46.0	57.5	21.3	46.0	1.1
Lesotho	62.7	70.0	75.6	73.0	28.6	57.2	19.3	42.1	57.2	54.7	68.4	25.4	54.7	1.3
Liberia	45.8	51.1	55.2	53.3	20.9	41.8	14.1	30.7	41.8	39.9	49.9	18.5	39.9	1.0
Madagascar	31.1	34.7	37.5	36.2	14.2	28.4	9.6	20.8	28.4	27.1	33.9	12.6	27.1	0.7
Malawi	64.6	83.0	90.5	84.1	4.5	80.3	46.1	83.7	80.3	88.0	89.5	30.0	88.0	6.1
Mali	35.8	40.0	43.1	41.7	16.3	32.7	11.0	24.0	32.7	31.2	39.0	14.5	31.2	0.8
Mauritania	44.0	63.7	68.5	55.2	46.2	47.5	23.3	32.4	47.5	25.5	54.8	28.1	25.5	6.7
Mexico	79.4	88.7	95.7	92.4	36.2	72.5	24.5	53.3	72.5	69.3	86.6	32.1	69.3	1.7
Morocco	59.6	66.6	71.9	69.4	27.2	54.4	18.4	40.0	54.4	52.0	65.0	24.1	52.0	1.3
Mozambique	53.2	59.4	64.1	61.9	24.3	48.5	16.4	35.7	48.5	46.4	57.9	21.5	46.4	1.1
Myanmar	30.4	33.9	36.6	35.4	13.9	27.7	9.4	20.4	27.7	26.5	33.1	12.3	26.5	0.7
Namibia	69.1	86.5	86.7	87.2	65.3	81.9	39.4	78.2	81.9	86.0	76.9	23.0	86.0	1.5
Nepal	54.1	55.5	56.7	56.6	15.3	44.3	39.9	53.3	44.3	52.8	55.8	37.5	52.8	18.9
Nicaragua	61.7	68.9	74.4	71.8	28.1	56.3	19.0	41.4	56.3	53.8	67.2	24.9	53.8	1.3
Niger	24.4	27.2	29.4	28.4	11.1	22.3	7.5	16.4	22.3	21.3	26.6	9.9	21.3	0.5
Nigeria	30.8	34.4	37.1	35.8	14.0	28.1	9.5	20.6	28.1	26.8	33.5	12.4	26.8	0.7
Pakistan	54.3	60.6	65.4	63.2	24.8	49.5	16.7	36.4	49.5	47.3	59.2	21.9	47.3	1.2
Panama	74.8	83.5	90.2	87.0	34.1	68.3	23.1	50.2	68.3	65.2	81.5	30.2	65.2	1.6
Papua New Guinea	42.5	47.4	51.2	49.4	19.4	38.8	13.1	28.5	38.8	37.0	46.3	17.2	37.0	0.9
Paraguay	76.4	85.3	92.1	88.9	34.9	69.8	23.6	51.3	69.8	66.7	83.3	30.9	66.7	1.6
Peru	74.4	83.1	89.7	86.6	33.9	67.9	22.9	49.9	67.9	64.9	81.1	30.1	64.9	1.6
Philippines	63.8	71.2	76.8	74.2	29.1	58.2	19.7	42.8	58.2	55.6	69.5	25.8	55.6	1.4
Rwanda	78.6	73.4	89.6	81.4	25.7	36.7	20.3	36.2	36.7	60.1	33.5	30.0	60.1	1.6
São Tomé and Príncipe	74.6	83.3	89.9	86.8	34.0	68.1	23.0	50.0	68.1	65.1	81.3	30.1	65.1	1.6
Senegal	68.5	68.9	69.1	68.7	36.0	52.3	7.4	50.5	52.3	59.2	61.1	35.1	59.2	8.1
Sierra Leone	65.4	71.4	75.8	69.9	16.2	66.3	43.6	32.5	66.3	67.8	69.6	34.5	67.8	0.8
Solomon Islands	69.3	77.4	83.5	80.6	31.6	63.2	21.4	46.5	63.2	60.4	75.5	28.0	60.4	1.5
Somalia	7.7	8.6	9.3	8.9	3.5	7.0	2.4	5.1	7.0	6.7	8.4	3.1	6.7	0.2
South Africa	78.7	87.8	94.8	91.5	35.9	71.8	24.2	52.7	71.8	68.6	85.7	31.8	68.6	1.7
South Sudan	9.6	10.7	11.5	11.1	4.4	8.7	2.9	6.4	8.7	8.3	10.4	3.9	8.3	0.2
Sudan	22.7	25.4	27.4	26.5	10.4	20.7	7.0	15.2	20.7	19.8	24.8	9.2	19.8	0.5
Suriname	75.7	84.5	91.2	88.1	34.5	69.1	23.3	50.7	69.1	66.0	82.5	30.6	66.0	1.6

Country	Clean birth env.	Immed-iate drying & stimul-ation	Therm-al protect-ion	Clean cord care	MROP	Anti-biotics for pPROM	Assist-ed vaginal delivery	Neo-natal resus	Parent-eral admin. of anti-biotics	Parenteral admin. of anti-convul-sants	Parent-eral admin. of utero-tonics	Rem-oval of RPCs	Ante-natal cortico-steroids	Labour induction for preg-nancies 41+ weeks
Tajikistan	72.3	80.8	87.2	84.2	33.0	66.0	22.3	48.5	66.0	63.1	78.8	29.2	63.1	1.6
Timor-Leste	39.8	44.4	48.0	46.3	18.1	36.3	12.3	26.7	36.3	34.7	43.4	16.1	34.7	0.9
Togo	42.8	55.4	60.7	46.4	36.1	48.5	8.1	12.8	48.5	10.9	55.4	16.9	10.9	1.1
Turkmen-istan	81.5	91.0	98.3	94.9	37.2	74.4	25.1	54.7	74.4	71.1	88.8	32.9	71.1	1.7
Uganda	57.7	56.8	72.5	63.6	24.2	50.0	25.6	36.2	50.0	40.9	65.6	28.4	40.9	11.5
Tanzania	51.6	57.4	61.7	60.8	24.5	32.2	15.8	53.6	32.2	49.9	57.1	15.3	49.9	6.5
Uzbekistan	79.8	89.1	96.2	92.9	36.4	72.8	24.6	53.5	72.8	69.6	87.0	32.3	69.6	1.7
Venezuela	77.9	87.0	93.9	90.7	35.5	71.1	24.0	52.2	71.1	67.9	84.9	31.5	67.9	1.7
Viet Nam	76.8	85.7	92.5	89.4	35.0	70.1	23.7	51.5	70.1	67.0	83.7	31.0	67.0	1.6
Yemen	25.2	28.1	30.3	29.3	11.5	22.9	7.8	16.9	22.9	21.9	27.4	10.2	21.9	0.5
Zambia	55.1	61.5	66.4	64.1	25.1	50.2	17.0	36.9	50.2	48.0	60.0	22.2	48.0	1.2
Zimbabwe	60.9	68.7	77.0	68.0	7.6	41.3	16.2	63.4	41.3	76.8	74.0	15.1	76.8	1.4

MROP: Manual removal of placenta. pPROM: premature prelabour rupture of membranes. RPCs: retained products of conception

Table B3. Baseline coverage of post-childbirth care interventions, 88 countries (%)

Country	Kangaroo mother care	Oral antibiotics for neonatal sepsis	Injectable antibiotics for neonatal sepsis
Afghanistan	0.0	0.0	48.3
Algeria	0.0	0.0	96.6
Angola	0.0	0.0	45.6
Azerbaijan	0.0	0.0	77.7
Bangladesh	0.0	0.0	37.6
Benin	0.0	0.0	83.9
Bhutan	0.0	0.0	63.1
Bolivia	0.0	0.0	67.5
Botswana	0.0	0.0	93.8
Brazil	0.0	0.0	98.1
Burkina Faso	0.0	0.0	66.3
Burundi	0.0	0.0	83.9
Cambodia	0.0	0.0	83.2
Cameroon	0.0	0.0	61.3
Central African Republic	0.0	0.0	52.1
Chad	0.0	0.0	21.9
China	0.0	0.0	99.2
Comoros	0.0	0.0	76.1
Congo	0.0	0.0	91.5
Côte d'Ivoire	0.0	0.0	69.8
DPR Korea	0.0	0.0	92.2
DR Congo	0.0	0.0	79.9
Djibouti	0.0	0.0	87.4
Dominican Republic	0.0	0.0	97.9
Egypt	0.0	0.0	86.7
Equatorial Guinea	0.0	0.0	67.3
Eritrea	0.0	0.0	33.6
Eswatini	0.0	0.0	87.7
Ethiopia	0.0	0.0	26.2
Gabon	0.0	0.0	90.2
Gambia	0.0	0.0	57.8
Ghana	0.0	0.0	78.7
Guatemala	0.0	0.0	65.0
Guinea	0.0	0.0	57.5
Guinea-Bissau	0.0	0.0	44.0
Guyana	0.0	0.0	92.4
Haiti	0.0	0.0	39.4
Honduras	0.0	0.0	82.7
India	0.0	0.0	78.9
Indonesia	0.0	0.0	63.6
Iraq	0.0	0.0	86.6
Jamaica	0.0	0.0	98.6
Kenya	0.0	0.0	61.5

Country	Kangaroo mother care	Oral antibiotics for neonatal sepsis	Injectable antibiotics for neonatal sepsis
Kyrgyzstan	0.0	0.0	99.6
Lao PDR	0.0	0.0	64.4
Lesotho	0.0	0.0	76.5
Liberia	0.0	0.0	55.8
Madagascar	0.0	0.0	37.9
Malawi	0.0	0.0	90.7
Mali	0.0	0.0	43.7
Mauritania	0.0	0.0	69.3
Mexico	0.0	0.0	96.9
Morocco	0.0	0.0	72.7
Mozambique	0.0	0.0	64.8
Myanmar	0.0	0.0	37.1
Namibia	0.0	0.0	87.7
Nepal	0.0	0.0	57.4
Nicaragua	0.0	0.0	75.2
Niger	0.0	0.0	29.8
Nigeria	0.0	0.0	37.5
Pakistan	0.0	0.0	66.2
Panama	0.0	0.0	91.2
Papua New Guinea	0.0	0.0	51.8
Paraguay	0.0	0.0	93.2
Peru	0.0	0.0	90.7
Philippines	0.0	0.0	77.7
Rwanda	0.0	0.0	90.7
São Tomé and Príncipe	0.0	0.0	91.0
Senegal	0.0	0.0	69.9
Sierra Leone	0.0	0.0	76.7
Solomon Islands	0.0	0.0	84.5
Somalia	0.0	0.0	9.4
South Africa	0.0	0.0	95.9
South Sudan	0.0	0.0	11.7
Sudan	0.0	0.0	27.7
Suriname	0.0	0.0	92.3
Tajikistan	0.0	0.0	88.2
Timor-Leste	0.0	0.0	48.5
Togo	0.0	0.0	61.4
Turkmenistan	0.0	0.0	99.4
Uganda	0.0	0.0	73.4
Tanzania	0.0	0.0	62.6
Uzbekistan	0.0	0.0	97.3
Venezuela	0.0	0.0	95.0
Viet Nam	0.0	0.0	93.6
Yemen	0.0	0.0	30.7
Zambia	0.0	0.0	67.1
Zimbabwe	0.0	0.0	77.0

C Target coverage values

The table below includes all of the target coverage values for all of the interventions modelled in each of the four scenarios discussed in detail in the paper.

Table C1. Target coverage values for all interventions, by modelling scenario and country, 88 countries (%)

Country	Intervention	10% Increase	25% increase	95% target	2% Attrition
Afghanistan	Folic acid supplementation/fortification	1.2	1.7	95.0	0.8
Afghanistan	Safe abortion services	46.7	68.6	95.0	33.0
Afghanistan	Post abortion case management	32.1	47.2	95.0	22.7
Afghanistan	Ectopic pregnancy case management	32.1	47.2	95.0	22.7
Afghanistan	TT - Tetanus toxoid vaccination	93.2	95.0	95.0	65.9
Afghanistan	IPTp	0.0	0.0	95.0	0.0
Afghanistan	Syphilis detection and treatment	19.4	28.5	95.0	13.7
Afghanistan	Iron supplementation in pregnancy	9.1	13.3	95.0	6.4
Afghanistan	Hypertensive disorder case management	5.7	8.4	95.0	4.0
Afghanistan	Diabetes case management	4.4	6.4	95.0	3.1
Afghanistan	Malaria case management	18.4	27.0	95.0	13.0
Afghanistan	MgSO4 management of pre-eclampsia	11.3	16.6	95.0	8.0
Afghanistan	Clean birth environment	52.7	77.3	95.0	37.3
Afghanistan	Immediate drying and additional stimulation	58.8	86.3	95.0	41.6
Afghanistan	Thermal protection	63.5	93.2	95.0	44.9
Afghanistan	Clean cord care	61.4	90.0	95.0	43.4
Afghanistan	Manual removal of placenta	24.1	35.4	95.0	17.0
Afghanistan	Parenteral administration of anti-convulsants	45.9	67.4	95.0	32.5
Afghanistan	Antibiotics for preterm or prolonged PROM	48.0	70.5	95.0	34.0
Afghanistan	Parenteral administration of antibiotics	48.0	70.5	95.0	34.0
Afghanistan	Assisted vaginal delivery	16.2	23.8	95.0	11.5
Afghanistan	Neonatal resuscitation	35.4	52.0	95.0	25.0
Afghanistan	Parenteral administration of uterotonics	57.5	84.4	95.0	40.7
Afghanistan	Removal of retained products of conception	21.3	31.3	95.0	15.1
Afghanistan	Induction of labour for post-term pregnancies	1.2	1.8	95.0	0.8
Afghanistan	Antenatal corticosteroids for preterm labour	45.9	67.4	95.0	32.5
Afghanistan	Breastfeeding promotion	55.8	81.8	95.0	39.4
Afghanistan	KMC - Kangaroo mother care	3.2	4.7	95.0	2.3
Afghanistan	Injectable antibiotics for neonatal sepsis	64.3	94.3	95.0	45.5
Algeria	Folic acid supplementation/fortification	4.5	6.6	95.0	3.2
Algeria	Safe abortion services	2.9	4.3	95.0	2.1
Algeria	Post abortion case management	95.0	95.0	95.0	68.2
Algeria	Ectopic pregnancy case management	95.0	95.0	95.0	68.2
Algeria	TT - Tetanus toxoid vaccination	98.0	98.0	98.0	92.2
Algeria	IPTp	0.0	0.0	95.0	0.0
Algeria	Syphilis detection and treatment	30.5	44.7	95.0	21.6
Algeria	Iron supplementation in pregnancy	0.0	0.0	95.0	0.0
Algeria	Hypertensive disorder case management	21.6	31.6	95.0	15.2
Algeria	Diabetes case management	16.8	24.6	95.0	11.9
Algeria	Malaria case management	69.3	95.0	95.0	49.0
Algeria	MgSO4 management of pre-eclampsia	42.6	62.5	95.0	30.1
Algeria	Clean birth environment	95.0	95.0	95.0	74.5
Algeria	Immediate drying and additional stimulation	95.0	95.0	95.0	83.3
Algeria	Thermal protection	95.5	95.5	95.5	89.9
Algeria	Clean cord care	95.0	95.0	95.0	86.8
Algeria	Manual removal of placenta	48.0	70.5	95.0	34.0
Algeria	Parenteral administration of anti-convulsants	92.0	95.0	95.0	65.0
Algeria	Antibiotics for preterm or prolonged PROM	95.0	95.0	95.0	68.0
Algeria	Parenteral administration of antibiotics	95.0	95.0	95.0	68.0
Algeria	Assisted vaginal delivery	32.5	47.7	95.0	23.0
Algeria	Neonatal resuscitation	70.7	95.0	95.0	50.0
Algeria	Parenteral administration of uterotonics	95.0	95.0	95.0	81.2
Algeria	Removal of retained products of conception	42.6	62.5	95.0	30.1
Algeria	Induction of labour for post-term pregnancies	2.3	3.3	95.0	1.6
Algeria	Antenatal corticosteroids for preterm labour	92.0	95.0	95.0	65.0
Algeria	Breastfeeding promotion	28.1	41.2	95.0	19.9
Algeria	KMC - Kangaroo mother care	6.4	9.4	95.0	4.5

Country	Intervention	10% Increase	25% increase	95% target	2% Attrition
Algeria	Oral antibiotics for neonatal sepsis	0.0	0.0	0.0	0.0
Algeria	Injectable antibiotics for neonatal sepsis	96.6	96.6	96.6	90.9
Angola	Folic acid supplementation/fortification	4.0	5.9	95.0	2.8
Angola	Safe abortion services	0.0	0.0	95.0	0.0
Angola	Post abortion case management	30.3	44.5	95.0	21.5
Angola	Ectopic pregnancy case management	30.3	44.5	95.0	21.5
Angola	TT - Tetanus toxoid vaccination	95.0	95.0	95.0	73.4
Angola	IPTp	51.1	75.0	95.0	36.1
Angola	Syphilis detection and treatment	26.6	39.1	95.0	18.8
Angola	Iron supplementation in pregnancy	42.7	62.7	95.0	30.2
Angola	Hypertensive disorder case management	19.3	28.3	95.0	13.6
Angola	Diabetes case management	15.0	22.1	95.0	10.6
Angola	Malaria case management	62.2	91.2	95.0	44.0
Angola	MgSO4 management of pre-eclampsia	38.1	55.9	95.0	26.9
Angola	Clean birth environment	49.8	73.0	95.0	35.2
Angola	Immediate drying and additional stimulation	55.6	81.6	95.0	39.3
Angola	Thermal protection	60.0	88.1	95.0	42.4
Angola	Clean cord care	57.9	85.0	95.0	40.9
Angola	Manual removal of placenta	22.8	33.4	95.0	16.1
Angola	Parenteral administration of anti-convulsants	43.4	63.7	95.0	30.7
Angola	Antibiotics for preterm or prolonged PROM	45.4	66.6	95.0	32.1
Angola	Parenteral administration of antibiotics	45.4	66.6	95.0	32.1
Angola	Assisted vaginal delivery	15.3	22.5	95.0	10.8
Angola	Neonatal resuscitation	33.4	49.0	95.0	23.6
Angola	Parenteral administration of uterotonics	54.3	79.7	95.0	38.4
Angola	Removal of retained products of conception	20.1	29.5	95.0	14.2
Angola	Induction of labour for post-term pregnancies	1.1	1.6	95.0	0.8
Angola	Antenatal corticosteroids for preterm labour	43.4	63.7	95.0	30.7
Angola	Breastfeeding promotion	42.3	62.1	95.0	29.9
Angola	KMC - Kangaroo mother care	3.0	4.5	95.0	2.1
Angola	Oral antibiotics for neonatal sepsis	0.0	0.0	0.0	0.0
Angola	Injectable antibiotics for neonatal sepsis	60.7	89.1	95.0	42.9
Azerbaijan	Folic acid supplementation/fortification	3.3	4.8	95.0	2.3
Azerbaijan	Safe abortion services	53.2	78.1	95.0	37.6
Azerbaijan	Post abortion case management	77.6	95.0	95.0	54.8
Azerbaijan	Ectopic pregnancy case management	77.6	95.0	95.0	54.8
Azerbaijan	TT - Tetanus toxoid vaccination	0.0	0.0	95.0	0.0
Azerbaijan	IPTp	0.0	0.0	95.0	0.0
Azerbaijan	Syphilis detection and treatment	26.1	38.3	95.0	18.4
Azerbaijan	Iron supplementation in pregnancy	2.1	3.1	95.0	1.5
Azerbaijan	Hypertensive disorder case management	15.7	23.0	95.0	11.1
Azerbaijan	Diabetes case management	12.2	18.0	95.0	8.7
Azerbaijan	Malaria case management	50.7	74.4	95.0	35.9
Azerbaijan	MgSO4 management of pre-eclampsia	31.1	45.7	95.0	22.0
Azerbaijan	Clean birth environment	84.8	95.0	95.0	60.0
Azerbaijan	Immediate drying and additional stimulation	94.6	95.0	95.0	66.9
Azerbaijan	Thermal protection	95.0	95.0	95.0	72.3
Azerbaijan	Clean cord care	95.0	95.0	95.0	69.7
Azerbaijan	Manual removal of placenta	38.6	56.6	95.0	27.3
Azerbaijan	Parenteral administration of anti-convulsants	73.9	95.0	95.0	52.2
Azerbaijan	Antibiotics for preterm or prolonged PROM	77.3	95.0	95.0	54.7
Azerbaijan	Parenteral administration of antibiotics	77.3	95.0	95.0	54.7
Azerbaijan	Assisted vaginal delivery	26.1	38.3	95.0	18.4
Azerbaijan	Neonatal resuscitation	56.8	83.4	95.0	40.2
Azerbaijan	Parenteral administration of uterotonics	92.4	95.0	95.0	65.3
Azerbaijan	Removal of retained products of conception	34.2	50.2	95.0	24.2
Azerbaijan	Induction of labour for post-term pregnancies	1.9	2.7	95.0	1.3
Azerbaijan	Antenatal corticosteroids for preterm labour	73.9	95.0	95.0	52.2
Azerbaijan	Breastfeeding promotion	11.4	16.8	95.0	8.1
Azerbaijan	KMC - Kangaroo mother care	5.2	7.6	95.0	3.7
Azerbaijan	Oral antibiotics for neonatal sepsis	0.0	0.0	0.0	0.0
Azerbaijan	Injectable antibiotics for neonatal sepsis	95.0	95.0	95.0	73.1
Bangladesh	Folic acid supplementation/fortification	2.1	3.0	95.0	1.5
Bangladesh	Safe abortion services	46.7	68.6	95.0	33.0
Bangladesh	Post abortion case management	25.0	36.7	95.0	17.7

Country	Intervention	10% Increase	25% increase	95% target	2% Attrition
Bangladesh	Ectopic pregnancy case management	25.0	36.7	95.0	17.7
Bangladesh	TT - Tetanus toxoid vaccination	98.0	98.0	98.0	92.2
Bangladesh	IPTp	0.0	0.0	95.0	0.0
Bangladesh	Syphilis detection and treatment	8.3	12.1	95.0	5.8
Bangladesh	Iron supplementation in pregnancy	0.0	0.0	95.0	0.0
Bangladesh	Hypertensive disorder case management	10.0	14.6	95.0	7.1
Bangladesh	Diabetes case management	14.1	20.7	95.0	10.0
Bangladesh	Malaria case management	32.2	47.3	95.0	22.8
Bangladesh	MgSO4 management of pre-eclampsia	9.1	13.3	95.0	6.4
Bangladesh	Clean birth environment	35.4	52.0	95.0	25.0
Bangladesh	Immediate drying and additional stimulation	36.9	54.1	95.0	26.1
Bangladesh	Thermal protection	48.2	70.7	95.0	34.1
Bangladesh	Clean cord care	47.8	70.1	95.0	33.8
Bangladesh	Manual removal of placenta	11.4	16.8	95.0	8.1
Bangladesh	Parenteral administration of anti-convulsants	35.5	52.1	95.0	25.1
Bangladesh	Antibiotics for preterm or prolonged PROM	22.4	32.8	95.0	15.8
Bangladesh	Parenteral administration of antibiotics	22.4	32.8	95.0	15.8
Bangladesh	Assisted vaginal delivery	24.8	36.3	95.0	17.5
Bangladesh	Neonatal resuscitation	37.5	55.1	95.0	26.5
Bangladesh	Parenteral administration of uterotonics	39.4	57.8	95.0	27.9
Bangladesh	Removal of retained products of conception	28.4	41.6	95.0	20.0
Bangladesh	Induction of labour for post-term pregnancies	9.6	14.1	95.0	6.8
Bangladesh	Antenatal corticosteroids for preterm labour	35.5	52.1	95.0	25.1
Bangladesh	Breastfeeding promotion	69.5	95.0	95.0	49.1
Bangladesh	KMC - Kangaroo mother care	2.5	3.7	95.0	1.8
Bangladesh	Oral antibiotics for neonatal sepsis	0.0	0.0	0.0	0.0
Bangladesh	Injectable antibiotics for neonatal sepsis	50.0	73.4	95.0	35.4
Benin	Folic acid supplementation/fortification	3.5	5.1	95.0	2.5
Benin	Safe abortion services	0.0	0.0	95.0	0.0
Benin	Post abortion case management	83.8	95.0	95.0	59.2
Benin	Ectopic pregnancy case management	83.8	95.0	95.0	59.2
Benin	TT - Tetanus toxoid vaccination	95.0	95.0	95.0	80.0
Benin	IPTp	44.5	65.2	95.0	31.4
Benin	Syphilis detection and treatment	27.8	40.8	95.0	19.7
Benin	Iron supplementation in pregnancy	38.1	55.9	95.0	26.9
Benin	Hypertensive disorder case management	16.1	23.6	95.0	11.4
Benin	Diabetes case management	9.5	13.9	95.0	6.7
Benin	Malaria case management	56.8	83.4	95.0	40.2
Benin	MgSO4 management of pre-eclampsia	37.9	55.7	95.0	26.8
Benin	Clean birth environment	95.0	95.0	95.0	76.6
Benin	Immediate drying and additional stimulation	95.0	95.0	95.0	78.4
Benin	Thermal protection	95.0	95.0	95.0	78.0
Benin	Clean cord care	95.0	95.0	95.0	76.6
Benin	Manual removal of placenta	92.1	95.0	95.0	65.1
Benin	Parenteral administration of anti-convulsants	63.1	92.6	95.0	44.6
Benin	Antibiotics for preterm or prolonged PROM	95.0	95.0	95.0	69.6
Benin	Parenteral administration of antibiotics	95.0	95.0	95.0	69.6
Benin	Assisted vaginal delivery	34.3	50.4	95.0	24.3
Benin	Neonatal resuscitation	61.4	90.0	95.0	43.4
Benin	Parenteral administration of uterotonics	95.0	95.0	95.0	76.6
Benin	Removal of retained products of conception	71.3	95.0	95.0	50.4
Benin	Induction of labour for post-term pregnancies	2.3	3.3	95.0	1.6
Benin	Antenatal corticosteroids for preterm labour	63.1	92.6	95.0	44.6
Benin	Breastfeeding promotion	0.0	0.0	95.0	0.0
Benin	KMC - Kangaroo mother care	5.6	8.2	95.0	3.9
Benin	Oral antibiotics for neonatal sepsis	0.0	0.0	0.0	0.0
Benin	Injectable antibiotics for neonatal sepsis	95.0	95.0	95.0	79.0
Bhutan	Folic acid supplementation/fortification	5.1	7.6	95.0	3.6
Bhutan	Safe abortion services	46.7	68.6	95.0	33.0
Bhutan	Post abortion case management	63.0	92.4	95.0	44.5
Bhutan	Ectopic pregnancy case management	63.0	92.4	95.0	44.5
Bhutan	TT - Tetanus toxoid vaccination	95.0	95.0	95.0	83.8
Bhutan	IPTp	0.0	0.0	95.0	0.0
Bhutan	Syphilis detection and treatment	31.9	46.9	95.0	22.6
Bhutan	Iron supplementation in pregnancy	0.0	0.0	95.0	0.0

Country	Intervention	10% Increase	25% increase	95% target	2% Attrition
Bhutan	Hypertensive disorder case management	24.8	36.3	95.0	17.5
Bhutan	Diabetes case management	19.3	28.3	95.0	13.6
Bhutan	Malaria case management	79.7	95.0	95.0	56.4
Bhutan	MgSO4 management of pre-eclampsia	49.0	71.9	95.0	34.6
Bhutan	Clean birth environment	68.9	95.0	95.0	48.8
Bhutan	Immediate drying and additional stimulation	76.9	95.0	95.0	54.4
Bhutan	Thermal protection	83.1	95.0	95.0	58.7
Bhutan	Clean cord care	80.1	95.0	95.0	56.7
Bhutan	Manual removal of placenta	31.4	46.1	95.0	22.2
Bhutan	Parenteral administration of anti-convulsants	60.0	88.1	95.0	42.4
Bhutan	Antibiotics for preterm or prolonged PROM	62.8	92.2	95.0	44.4
Bhutan	Parenteral administration of antibiotics	62.8	92.2	95.0	44.4
Bhutan	Assisted vaginal delivery	21.3	31.3	95.0	15.1
Bhutan	Neonatal resuscitation	46.2	67.8	95.0	32.7
Bhutan	Parenteral administration of uterotonics	75.1	95.0	95.0	53.1
Bhutan	Removal of retained products of conception	27.8	40.8	95.0	19.7
Bhutan	Induction of labour for post-term pregnancies	1.5	2.1	95.0	1.0
Bhutan	Antenatal corticosteroids for preterm labour	60.0	88.1	95.0	42.4
Bhutan	Breastfeeding promotion	58.4	85.7	95.0	41.3
Bhutan	KMC - Kangaroo mother care	4.2	6.2	95.0	3.0
Bhutan	Oral antibiotics for neonatal sepsis	0.0	0.0	0.0	0.0
Bhutan	Injectable antibiotics for neonatal sepsis	84.0	95.0	95.0	59.4
Bolivia	Folic acid supplementation/fortification	4.8	7.0	95.0	3.4
Bolivia	Safe abortion services	0.3	0.4	95.0	0.2
Bolivia	Post abortion case management	67.4	95.0	95.0	47.6
Bolivia	Ectopic pregnancy case management	67.4	95.0	95.0	47.6
Bolivia	TT - Tetanus toxoid vaccination	95.0	95.0	95.0	81.9
Bolivia	IPTp	0.0	0.0	95.0	0.0
Bolivia	Syphilis detection and treatment	29.7	43.6	95.0	21.0
Bolivia	Iron supplementation in pregnancy	33.4	49.0	95.0	23.6
Bolivia	Hypertensive disorder case management	22.9	33.6	95.0	16.2
Bolivia	Diabetes case management	17.8	26.2	95.0	12.6
Bolivia	Malaria case management	73.7	95.0	95.0	52.1
Bolivia	MgSO4 management of pre-eclampsia	45.3	66.4	95.0	32.0
Bolivia	Clean birth environment	73.7	95.0	95.0	52.1
Bolivia	Immediate drying and additional stimulation	82.3	95.0	95.0	58.2
Bolivia	Thermal protection	88.8	95.0	95.0	62.8
Bolivia	Clean cord care	85.7	95.0	95.0	60.6
Bolivia	Manual removal of placenta	33.5	49.2	95.0	23.7
Bolivia	Parenteral administration of anti-convulsants	64.3	94.3	95.0	45.5
Bolivia	Antibiotics for preterm or prolonged PROM	67.2	95.0	95.0	47.5
Bolivia	Parenteral administration of antibiotics	67.2	95.0	95.0	47.5
Bolivia	Assisted vaginal delivery	22.8	33.4	95.0	16.1
Bolivia	Neonatal resuscitation	49.4	72.5	95.0	34.9
Bolivia	Parenteral administration of uterotonics	80.3	95.0	95.0	56.8
Bolivia	Removal of retained products of conception	29.8	43.8	95.0	21.1
Bolivia	Induction of labour for post-term pregnancies	1.6	2.3	95.0	1.1
Bolivia	Antenatal corticosteroids for preterm labour	64.3	94.3	95.0	45.5
Bolivia	Breastfeeding promotion	79.5	95.0	95.0	56.2
Bolivia	KMC - Kangaroo mother care	4.5	6.6	95.0	3.2
Bolivia	Oral antibiotics for neonatal sepsis	0.0	0.0	0.0	0.0
Bolivia	Injectable antibiotics for neonatal sepsis	89.8	95.0	95.0	63.5
Botswana	Folic acid supplementation/fortification	4.9	7.2	95.0	3.4
Botswana	Safe abortion services	56.4	82.8	95.0	39.9
Botswana	Post abortion case management	93.6	95.0	95.0	66.2
Botswana	Ectopic pregnancy case management	93.6	95.0	95.0	66.2
Botswana	TT - Tetanus toxoid vaccination	95.0	95.0	95.0	87.5
Botswana	IPTp	0.0	0.0	95.0	0.0
Botswana	Syphilis detection and treatment	24.1	35.4	95.0	17.0
Botswana	Iron supplementation in pregnancy	0.0	0.0	95.0	0.0
Botswana	Hypertensive disorder case management	23.4	34.4	95.0	16.6
Botswana	Diabetes case management	18.2	26.8	95.0	12.9
Botswana	Malaria case management	75.6	95.0	95.0	53.5
Botswana	MgSO4 management of pre-eclampsia	46.3	68.0	95.0	32.8
Botswana	Clean birth environment	95.0	95.0	95.0	72.4

Country	Intervention	10% Increase	25% increase	95% target	2% Attrition
Botswana	Immediate drying and additional stimulation	95.0	95.0	95.0	80.8
Botswana	Thermal protection	95.0	95.0	95.0	87.2
Botswana	Clean cord care	95.0	95.0	95.0	84.2
Botswana	Manual removal of placenta	46.7	68.6	95.0	33.0
Botswana	Parenteral administration of anti-convulsants	89.3	95.0	95.0	63.2
Botswana	Antibiotics for preterm or prolonged PROM	93.4	95.0	95.0	66.1
Botswana	Parenteral administration of antibiotics	93.4	95.0	95.0	66.1
Botswana	Assisted vaginal delivery	31.5	46.3	95.0	22.3
Botswana	Neonatal resuscitation	68.7	95.0	95.0	48.6
Botswana	Parenteral administration of uterotonics	95.0	95.0	95.0	78.9
Botswana	Removal of retained products of conception	41.4	60.7	95.0	29.3
Botswana	Induction of labour for post-term pregnancies	2.3	3.3	95.0	1.6
Botswana	Antenatal corticosteroids for preterm labour	89.3	95.0	95.0	63.2
Botswana	Breastfeeding promotion	0.0	0.0	95.0	0.0
Botswana	KMC - Kangaroo mother care	6.2	9.2	95.0	4.4
Botswana	Oral antibiotics for neonatal sepsis	0.0	0.0	0.0	0.0
Botswana	Injectable antibiotics for neonatal sepsis	95.0	95.0	95.0	88.3
Brazil	Folic acid supplementation/fortification	6.1	8.9	95.0	4.3
Brazil	Safe abortion services	0.3	0.4	95.0	0.2
Brazil	Post abortion case management	95.0	95.0	95.0	69.2
Brazil	Ectopic pregnancy case management	95.0	95.0	95.0	69.2
Brazil	TT - Tetanus toxoid vaccination	95.0	95.0	95.0	88.5
Brazil	IPTp	0.0	0.0	95.0	0.0
Brazil	Syphilis detection and treatment	29.9	43.9	95.0	21.2
Brazil	Iron supplementation in pregnancy	0.0	0.0	95.0	0.0
Brazil	Hypertensive disorder case management	29.0	42.6	95.0	20.5
Brazil	Diabetes case management	22.6	33.2	95.0	16.0
Brazil	Malaria case management	93.8	95.0	95.0	66.4
Brazil	MgSO4 management of pre-eclampsia	57.5	84.4	95.0	40.7
Brazil	Clean birth environment	95.0	95.0	95.0	75.8
Brazil	Immediate drying and additional stimulation	95.0	95.0	95.0	84.5
Brazil	Thermal protection	97.0	97.0	97.0	91.3
Brazil	Clean cord care	95.0	95.0	95.0	88.1
Brazil	Manual removal of placenta	48.8	71.7	95.0	34.5
Brazil	Parenteral administration of anti-convulsants	93.3	95.0	95.0	66.0
Brazil	Antibiotics for preterm or prolonged PROM	95.0	95.0	95.0	69.1
Brazil	Parenteral administration of antibiotics	95.0	95.0	95.0	69.1
Brazil	Assisted vaginal delivery	33.0	48.4	95.0	23.3
Brazil	Neonatal resuscitation	71.7	95.0	95.0	50.7
Brazil	Parenteral administration of uterotonics	95.0	95.0	95.0	82.5
Brazil	Removal of retained products of conception	43.3	63.5	95.0	30.6
Brazil	Induction of labour for post-term pregnancies	2.3	3.3	95.0	1.6
Brazil	Antenatal corticosteroids for preterm labour	93.3	95.0	95.0	66.0
Brazil	Breastfeeding promotion	36.5	53.5	95.0	25.8
Brazil	KMC - Kangaroo mother care	6.5	9.6	95.0	4.6
Brazil	Oral antibiotics for neonatal sepsis	0.0	0.0	0.0	0.0
Brazil	Injectable antibiotics for neonatal sepsis	98.1	98.1	98.1	92.3
Burkina Faso	Folic acid supplementation/fortification	2.2	3.2	95.0	1.6
Burkina Faso	Safe abortion services	0.0	0.0	95.0	0.0
Burkina Faso	Post abortion case management	66.2	95.0	95.0	46.8
Burkina Faso	Ectopic pregnancy case management	66.2	95.0	95.0	46.8
Burkina Faso	TT - Tetanus toxoid vaccination	95.0	95.0	95.0	86.6
Burkina Faso	IPTp	63.4	93.0	95.0	44.8
Burkina Faso	Syphilis detection and treatment	11.2	16.4	95.0	7.9
Burkina Faso	Iron supplementation in pregnancy	66.8	95.0	95.0	47.2
Burkina Faso	Hypertensive disorder case management	8.5	12.5	95.0	6.0
Burkina Faso	Diabetes case management	0.9	1.4	95.0	0.7
Burkina Faso	Malaria case management	30.1	44.1	95.0	21.3
Burkina Faso	MgSO4 management of pre-eclampsia	9.2	13.5	95.0	6.5
Burkina Faso	Clean birth environment	85.2	95.0	95.0	60.2
Burkina Faso	Immediate drying and additional stimulation	85.1	95.0	95.0	60.1
Burkina Faso	Thermal protection	87.3	95.0	95.0	61.7
Burkina Faso	Clean cord care	87.2	95.0	95.0	61.6
Burkina Faso	Manual removal of placenta	65.4	95.0	95.0	46.2
Burkina Faso	Parenteral administration of anti-convulsants	21.2	31.1	95.0	15.0

Country	Intervention	10% Increase	25% increase	95% target	2% Attrition
Burkina Faso	Antibiotics for preterm or prolonged PROM	70.0	95.0	95.0	49.5
Burkina Faso	Parenteral administration of antibiotics	70.0	95.0	95.0	49.5
Burkina Faso	Assisted vaginal delivery	9.3	13.7	95.0	6.6
Burkina Faso	Neonatal resuscitation	17.3	25.4	95.0	12.2
Burkina Faso	Parenteral administration of uterotonics	85.3	95.0	95.0	60.3
Burkina Faso	Removal of retained products of conception	14.8	21.7	95.0	10.4
Burkina Faso	Induction of labour for post-term pregnancies	1.2	1.8	95.0	0.8
Burkina Faso	Antenatal corticosteroids for preterm labour	21.2	31.1	95.0	15.0
Burkina Faso	Breastfeeding promotion	29.4	43.2	95.0	20.8
Burkina Faso	KMC - Kangaroo mother care	4.4	6.5	95.0	3.1
Burkina Faso	Oral antibiotics for neonatal sepsis	0.0	0.0	0.0	0.0
Burkina Faso	Injectable antibiotics for neonatal sepsis	88.2	95.0	95.0	62.4
Burundi	Folic acid supplementation/fortification	3.4	5.0	95.0	2.4
Burundi	Safe abortion services	4.4	6.4	95.0	3.1
Burundi	Post abortion case management	83.8	95.0	95.0	59.2
Burundi	Ectopic pregnancy case management	83.8	95.0	95.0	59.2
Burundi	TT - Tetanus toxoid vaccination	95.0	95.0	95.0	81.9
Burundi	IPTp	0.0	0.0	95.0	0.0
Burundi	Syphilis detection and treatment	32.6	47.9	95.0	23.1
Burundi	Iron supplementation in pregnancy	1.9	2.7	95.0	1.3
Burundi	Hypertensive disorder case management	16.2	23.8	95.0	11.5
Burundi	Diabetes case management	12.6	18.6	95.0	8.9
Burundi	Malaria case management	52.3	76.8	95.0	37.0
Burundi	MgSO4 management of pre-eclampsia	32.1	47.1	95.0	22.7
Burundi	Clean birth environment	91.6	95.0	95.0	64.8
Burundi	Immediate drying and additional stimulation	95.0	95.0	95.0	72.4
Burundi	Thermal protection	95.0	95.0	95.0	78.1
Burundi	Clean cord care	95.0	95.0	95.0	75.4
Burundi	Manual removal of placenta	41.8	61.3	95.0	29.6
Burundi	Parenteral administration of anti-convulsants	79.9	95.0	95.0	56.5
Burundi	Antibiotics for preterm or prolonged PROM	83.6	95.0	95.0	59.1
Burundi	Parenteral administration of antibiotics	83.6	95.0	95.0	59.1
Burundi	Assisted vaginal delivery	28.2	41.4	95.0	20.0
Burundi	Neonatal resuscitation	61.5	90.2	95.0	43.5
Burundi	Parenteral administration of uterotonics	95.0	95.0	95.0	70.6
Burundi	Removal of retained products of conception	37.0	54.3	95.0	26.2
Burundi	Induction of labour for post-term pregnancies	2.0	2.9	95.0	1.4
Burundi	Antenatal corticosteroids for preterm labour	79.9	95.0	95.0	56.5
Burundi	Breastfeeding promotion	95.0	95.0	95.0	76.0
Burundi	KMC - Kangaroo mother care	5.6	8.2	95.0	3.9
Burundi	Oral antibiotics for neonatal sepsis	0.0	0.0	0.0	0.0
Burundi	Injectable antibiotics for neonatal sepsis	95.0	95.0	95.0	79.0
Cambodia	Folic acid supplementation/fortification	5.0	7.4	95.0	3.6
Cambodia	Safe abortion services	51.9	76.2	95.0	36.7
Cambodia	Post abortion case management	83.1	95.0	95.0	58.7
Cambodia	Ectopic pregnancy case management	83.1	95.0	95.0	58.7
Cambodia	TT - Tetanus toxoid vaccination	95.0	95.0	95.0	87.5
Cambodia	IPTp	0.0	0.0	95.0	0.0
Cambodia	Syphilis detection and treatment	31.5	46.3	95.0	22.3
Cambodia	Iron supplementation in pregnancy	95.0	95.0	95.0	71.1
Cambodia	Hypertensive disorder case management	24.2	35.5	95.0	17.1
Cambodia	Diabetes case management	18.9	27.7	95.0	13.4
Cambodia	Malaria case management	78.1	95.0	95.0	55.2
Cambodia	MgSO4 management of pre-eclampsia	47.9	70.3	95.0	33.9
Cambodia	Clean birth environment	90.9	95.0	95.0	64.3
Cambodia	Immediate drying and additional stimulation	95.0	95.0	95.0	71.7
Cambodia	Thermal protection	95.0	95.0	95.0	77.5
Cambodia	Clean cord care	95.0	95.0	95.0	74.7
Cambodia	Manual removal of placenta	41.4	60.7	95.0	29.3
Cambodia	Parenteral administration of anti-convulsants	79.2	95.0	95.0	56.0
Cambodia	Antibiotics for preterm or prolonged PROM	82.9	95.0	95.0	58.6
Cambodia	Parenteral administration of antibiotics	82.9	95.0	95.0	58.6
Cambodia	Assisted vaginal delivery	28.0	41.0	95.0	19.8
Cambodia	Neonatal resuscitation	61.0	89.5	95.0	43.1
Cambodia	Parenteral administration of uterotonics	95.0	95.0	95.0	70.0

Country	Intervention	10% Increase	25% increase	95% target	2% Attrition
Cambodia	Removal of retained products of conception	36.7	53.9	95.0	26.0
Cambodia	Induction of labour for post-term pregnancies	2.0	2.9	95.0	1.4
Cambodia	Antenatal corticosteroids for preterm labour	79.2	95.0	95.0	56.0
Cambodia	Breastfeeding promotion	84.7	95.0	95.0	59.9
Cambodia	KMC - Kangaroo mother care	5.5	8.1	95.0	3.9
Cambodia	Oral antibiotics for neonatal sepsis	0.0	0.0	0.0	0.0
Cambodia	Injectable antibiotics for neonatal sepsis	95.0	95.0	95.0	78.3
Cameroon	Folic acid supplementation/fortification	3.9	5.7	95.0	2.8
Cameroon	Safe abortion services	0.0	0.0	95.0	0.0
Cameroon	Post abortion case management	61.2	89.8	95.0	43.3
Cameroon	Ectopic pregnancy case management	61.2	89.8	95.0	43.3
Cameroon	TT - Tetanus toxoid vaccination	95.0	95.0	95.0	80.0
Cameroon	IPTp	59.0	86.5	95.0	41.7
Cameroon	Syphilis detection and treatment	27.3	40.0	95.0	19.3
Cameroon	Iron supplementation in pregnancy	72.0	95.0	95.0	50.9
Cameroon	Hypertensive disorder case management	18.8	27.5	95.0	13.3
Cameroon	Diabetes case management	14.6	21.5	95.0	10.4
Cameroon	Malaria case management	60.7	89.1	95.0	42.9
Cameroon	MgSO4 management of pre-eclampsia	37.3	54.7	95.0	26.4
Cameroon	Clean birth environment	66.8	95.0	95.0	47.2
Cameroon	Immediate drying and additional stimulation	74.7	95.0	95.0	52.8
Cameroon	Thermal protection	80.5	95.0	95.0	56.9
Cameroon	Clean cord care	77.9	95.0	95.0	55.1
Cameroon	Manual removal of placenta	30.5	44.7	95.0	21.6
Cameroon	Parenteral administration of anti-convulsants	58.3	85.5	95.0	41.2
Cameroon	Antibiotics for preterm or prolonged PROM	61.0	89.5	95.0	43.1
Cameroon	Parenteral administration of antibiotics	61.0	89.5	95.0	43.1
Cameroon	Assisted vaginal delivery	20.6	30.3	95.0	14.6
Cameroon	Neonatal resuscitation	44.9	65.8	95.0	31.7
Cameroon	Parenteral administration of uterotonics	72.8	95.0	95.0	51.5
Cameroon	Removal of retained products of conception	27.0	39.6	95.0	19.1
Cameroon	Induction of labour for post-term pregnancies	1.5	2.1	95.0	1.0
Cameroon	Antenatal corticosteroids for preterm labour	58.3	85.5	95.0	41.2
Cameroon	Breastfeeding promotion	35.1	51.6	95.0	24.8
Cameroon	KMC - Kangaroo mother care	4.1	6.0	95.0	2.9
Cameroon	Oral antibiotics for neonatal sepsis	0.0	0.0	0.0	0.0
Cameroon	Injectable antibiotics for neonatal sepsis	81.6	95.0	95.0	57.7
Central African Republic	Folic acid supplementation/fortification	2.5	3.7	95.0	1.8
Central African Republic	Safe abortion services	0.0	0.0	95.0	0.0
Central African Republic	Post abortion case management	52.0	76.3	95.0	36.8
Central African Republic	Ectopic pregnancy case management	52.0	76.3	95.0	36.8
Central African Republic	TT - Tetanus toxoid vaccination	79.9	95.0	95.0	56.5
Central African Republic	IPTp	35.1	51.6	95.0	24.8
Central African Republic	Syphilis detection and treatment	22.5	33.0	95.0	15.9
Central African Republic	Iron supplementation in pregnancy	0.0	0.0	95.0	0.0
Central African Republic	Hypertensive disorder case management	12.2	18.0	95.0	8.7
Central African Republic	Diabetes case management	9.5	13.9	95.0	6.7
Central African Republic	Malaria case management	39.4	57.8	95.0	27.9
Central African Republic	MgSO4 management of pre-eclampsia	24.1	35.4	95.0	17.0
Central African Republic	Clean birth environment	56.8	83.4	95.0	40.2
Central African Republic	Immediate drying and additional stimulation	63.5	93.2	95.0	44.9
Central African Republic	Thermal protection	68.5	95.0	95.0	48.5
Central African Republic	Clean cord care	66.2	95.0	95.0	46.8
Central African Republic	Manual removal of placenta	26.0	38.1	95.0	18.4
Central African Republic	Parenteral administration of anti-convulsants	49.5	72.7	95.0	35.0
Central African Republic	Antibiotics for preterm or prolonged PROM	51.9	76.2	95.0	36.7
Central African Republic	Parenteral administration of antibiotics	51.9	76.2	95.0	36.7
Central African Republic	Assisted vaginal delivery	17.6	25.8	95.0	12.4
Central African Republic	Neonatal resuscitation	38.1	55.9	95.0	26.9
Central African Republic	Parenteral administration of uterotonics	61.9	90.8	95.0	43.8
Central African Republic	Removal of retained products of conception	23.0	33.8	95.0	16.3
Central African Republic	Induction of labour for post-term pregnancies	1.2	1.8	95.0	0.8
Central African Republic	Antenatal corticosteroids for preterm labour	49.5	72.7	95.0	35.0
Central African Republic	Breastfeeding promotion	34.3	50.4	95.0	24.3
Central African Republic	KMC - Kangaroo mother care	3.5	5.1	95.0	2.5

Country	Intervention	10% Increase	25% increase	95% target	2% Attrition
Central African Republic	Oral antibiotics for neonatal sepsis	0.0	0.0	0.0	0.0
Central African Republic	Injectable antibiotics for neonatal sepsis	69.3	95.0	95.0	49.0
Chad	Folic acid supplementation/fortification	2.1	3.1	95.0	1.5
Chad	Safe abortion services	0.0	0.0	95.0	0.0
Chad	Post abortion case management	2.9	4.3	95.0	2.1
Chad	Ectopic pregnancy case management	2.9	4.3	95.0	2.1
Chad	TT - Tetanus toxoid vaccination	95.0	95.0	95.0	73.4
Chad	IPTp	24.1	35.4	95.0	17.0
Chad	Syphilis detection and treatment	18.2	26.8	95.0	12.9
Chad	Iron supplementation in pregnancy	14.6	21.5	95.0	10.4
Chad	Hypertensive disorder case management	10.0	14.6	95.0	7.1
Chad	Diabetes case management	7.9	11.5	95.0	5.6
Chad	Malaria case management	32.3	47.5	95.0	22.9
Chad	MgSO4 management of pre-eclampsia	19.8	29.1	95.0	14.0
Chad	Clean birth environment	23.8	35.0	95.0	16.8
Chad	Immediate drying and additional stimulation	26.6	39.1	95.0	18.8
Chad	Thermal protection	28.7	42.2	95.0	20.3
Chad	Clean cord care	27.8	40.8	95.0	19.7
Chad	Manual removal of placenta	10.9	16.0	95.0	7.7
Chad	Parenteral administration of anti-convulsants	20.8	30.5	95.0	14.7
Chad	Antibiotics for preterm or prolonged PROM	21.8	32.0	95.0	15.4
Chad	Parenteral administration of antibiotics	21.8	32.0	95.0	15.4
Chad	Assisted vaginal delivery	7.3	10.7	95.0	5.2
Chad	Neonatal resuscitation	16.0	23.4	95.0	11.3
Chad	Parenteral administration of uterotonics	26.0	38.1	95.0	18.4
Chad	Removal of retained products of conception	9.6	14.1	95.0	6.8
Chad	Induction of labour for post-term pregnancies	0.5	0.8	95.0	0.4
Chad	Antenatal corticosteroids for preterm labour	20.8	30.5	95.0	14.7
Chad	Breastfeeding promotion	0.4	0.6	95.0	0.3
Chad	KMC - Kangaroo mother care	1.5	2.1	95.0	1.0
Chad	Oral antibiotics for neonatal sepsis	0.0	0.0	0.0	0.0
Chad	Injectable antibiotics for neonatal sepsis	29.1	42.8	95.0	20.6
China	Folic acid supplementation/fortification	0.0	0.0	95.0	0.0
China	Safe abortion services	100.0	100.0	100.0	94.1
China	Post abortion case management	95.0	95.0	95.0	70.0
China	Ectopic pregnancy case management	95.0	95.0	95.0	70.0
China	TT - Tetanus toxoid vaccination	0.0	0.0	95.0	0.0
China	IPTp	0.0	0.0	95.0	0.0
China	Syphilis detection and treatment	0.0	0.0	95.0	0.0
China	Iron supplementation in pregnancy	0.0	0.0	95.0	0.0
China	Hypertensive disorder case management	0.0	0.0	95.0	0.0
China	Diabetes case management	0.0	0.0	95.0	0.0
China	Malaria case management	0.0	0.0	95.0	0.0
China	MgSO4 management of pre-eclampsia	0.0	0.0	95.0	0.0
China	Clean birth environment	95.0	95.0	95.0	76.6
China	Immediate drying and additional stimulation	95.0	95.0	95.0	85.5
China	Thermal protection	98.0	98.0	98.0	92.2
China	Clean cord care	95.0	95.0	95.0	89.1
China	Manual removal of placenta	49.4	72.5	95.0	34.9
China	Parenteral administration of anti-convulsants	94.4	95.0	95.0	66.7
China	Antibiotics for preterm or prolonged PROM	95.0	95.0	95.0	69.8
China	Parenteral administration of antibiotics	95.0	95.0	95.0	69.8
China	Assisted vaginal delivery	33.4	49.0	95.0	23.6
China	Neonatal resuscitation	72.5	95.0	95.0	51.3
China	Parenteral administration of uterotonics	95.0	95.0	95.0	83.4
China	Removal of retained products of conception	43.8	64.3	95.0	31.0
China	Induction of labour for post-term pregnancies	2.3	3.3	95.0	1.6
China	Antenatal corticosteroids for preterm labour	94.4	95.0	95.0	66.7
China	Breastfeeding promotion	0.0	0.0	95.0	0.0
China	KMC - Kangaroo mother care	6.6	9.7	95.0	4.7
China	Oral antibiotics for neonatal sepsis	0.0	0.0	0.0	0.0
China	Injectable antibiotics for neonatal sepsis	99.2	99.2	99.2	93.4
Comoros	Folic acid supplementation/fortification	3.3	4.8	95.0	2.3
Comoros	Safe abortion services	4.4	6.4	95.0	3.1
Comoros	Post abortion case management	76.0	95.0	95.0	53.7

Country	Intervention	10% Increase	25% increase	95% target	2% Attrition
Comoros	Ectopic pregnancy case management	76.0	95.0	95.0	53.7
Comoros	TT - Tetanus toxoid vaccination	95.0	95.0	95.0	80.0
Comoros	IPTp	41.0	60.2	95.0	29.0
Comoros	Syphilis detection and treatment	30.3	44.5	95.0	21.5
Comoros	Iron supplementation in pregnancy	16.8	24.6	95.0	11.9
Comoros	Hypertensive disorder case management	15.7	23.0	95.0	11.1
Comoros	Diabetes case management	12.2	18.0	95.0	8.7
Comoros	Malaria case management	50.8	74.6	95.0	36.0
Comoros	MgSO4 management of pre-eclampsia	31.1	45.7	95.0	22.0
Comoros	Clean birth environment	83.1	95.0	95.0	58.7
Comoros	Immediate drying and additional stimulation	92.8	95.0	95.0	65.6
Comoros	Thermal protection	95.0	95.0	95.0	70.9
Comoros	Clean cord care	95.0	95.0	95.0	68.4
Comoros	Manual removal of placenta	37.9	55.7	95.0	26.8
Comoros	Parenteral administration of anti-convulsants	72.4	95.0	95.0	51.2
Comoros	Antibiotics for preterm or prolonged PROM	75.9	95.0	95.0	53.6
Comoros	Parenteral administration of antibiotics	75.9	95.0	95.0	53.6
Comoros	Assisted vaginal delivery	25.6	37.5	95.0	18.1
Comoros	Neonatal resuscitation	55.8	81.8	95.0	39.4
Comoros	Parenteral administration of uterotonics	90.5	95.0	95.0	64.0
Comoros	Removal of retained products of conception	33.5	49.2	95.0	23.7
Comoros	Induction of labour for post-term pregnancies	1.7	2.5	95.0	1.2
Comoros	Antenatal corticosteroids for preterm labour	72.4	95.0	95.0	51.2
Comoros	Breastfeeding promotion	14.2	20.9	95.0	10.1
Comoros	KMC - Kangaroo mother care	5.1	7.4	95.0	3.6
Comoros	Oral antibiotics for neonatal sepsis	0.0	0.0	0.0	0.0
Comoros	Injectable antibiotics for neonatal sepsis	95.0	95.0	95.0	71.6
Congo	Folic acid supplementation/fortification	5.3	7.7	95.0	3.7
Congo	Safe abortion services	0.0	0.0	95.0	0.0
Congo	Post abortion case management	91.3	95.0	95.0	64.6
Congo	Ectopic pregnancy case management	91.3	95.0	95.0	64.6
Congo	TT - Tetanus toxoid vaccination	95.0	95.0	95.0	80.0
Congo	IPTp	30.9	45.3	95.0	21.8
Congo	Syphilis detection and treatment	30.6	44.9	95.0	21.6
Congo	Iron supplementation in pregnancy	57.1	83.8	95.0	40.4
Congo	Hypertensive disorder case management	25.3	37.1	95.0	17.9
Congo	Diabetes case management	19.7	28.9	95.0	13.9
Congo	Malaria case management	81.5	95.0	95.0	57.6
Congo	MgSO4 management of pre-eclampsia	49.9	73.2	95.0	35.3
Congo	Clean birth environment	95.0	95.0	95.0	70.6
Congo	Immediate drying and additional stimulation	95.0	95.0	95.0	78.9
Congo	Thermal protection	95.0	95.0	95.0	85.1
Congo	Clean cord care	95.0	95.0	95.0	82.2
Congo	Manual removal of placenta	45.5	66.8	95.0	32.2
Congo	Parenteral administration of anti-convulsants	87.0	95.0	95.0	61.6
Congo	Antibiotics for preterm or prolonged PROM	91.2	95.0	95.0	64.5
Congo	Parenteral administration of antibiotics	91.2	95.0	95.0	64.5
Congo	Assisted vaginal delivery	30.7	45.1	95.0	21.7
Congo	Neonatal resuscitation	66.9	95.0	95.0	47.3
Congo	Parenteral administration of uterotonics	95.0	95.0	95.0	77.0
Congo	Removal of retained products of conception	40.3	59.2	95.0	28.5
Congo	Induction of labour for post-term pregnancies	2.1	3.1	95.0	1.5
Congo	Antenatal corticosteroids for preterm labour	87.0	95.0	95.0	61.6
Congo	Breastfeeding promotion	42.2	61.9	95.0	29.8
Congo	KMC - Kangaroo mother care	6.1	8.9	95.0	4.3
Congo	Oral antibiotics for neonatal sepsis	0.0	0.0	0.0	0.0
Congo	Injectable antibiotics for neonatal sepsis	95.0	95.0	95.0	86.1
Côte d'Ivoire	Folic acid supplementation/fortification	3.4	5.0	95.0	2.4
Côte d'Ivoire	Safe abortion services	0.0	0.0	95.0	0.0
Côte d'Ivoire	Post abortion case management	69.7	95.0	95.0	49.3
Côte d'Ivoire	Ectopic pregnancy case management	69.7	95.0	95.0	49.3
Côte d'Ivoire	TT - Tetanus toxoid vaccination	95.0	95.0	95.0	80.0
Côte d'Ivoire	IPTp	58.3	85.5	95.0	41.2
Côte d'Ivoire	Syphilis detection and treatment	30.6	44.9	95.0	21.6
Côte d'Ivoire	Iron supplementation in pregnancy	33.3	48.8	95.0	23.5

Country	Intervention	10% Increase	25% increase	95% target	2% Attrition
Côte d'Ivoire	Hypertensive disorder case management	16.4	24.0	95.0	11.6
Côte d'Ivoire	Diabetes case management	12.8	18.8	95.0	9.0
Côte d'Ivoire	Malaria case management	52.8	77.5	95.0	37.4
Côte d'Ivoire	MgSO4 management of pre-eclampsia	32.5	47.7	95.0	23.0
Côte d'Ivoire	Clean birth environment	76.1	95.0	95.0	53.8
Côte d'Ivoire	Immediate drying and additional stimulation	85.1	95.0	95.0	60.1
Côte d'Ivoire	Thermal protection	91.8	95.0	95.0	64.9
Côte d'Ivoire	Clean cord care	88.6	95.0	95.0	62.7
Côte d'Ivoire	Manual removal of placenta	34.7	51.0	95.0	24.6
Côte d'Ivoire	Parenteral administration of anti-convulsants	66.4	95.0	95.0	47.0
Côte d'Ivoire	Antibiotics for preterm or prolonged PROM	69.5	95.0	95.0	49.1
Côte d'Ivoire	Parenteral administration of antibiotics	69.5	95.0	95.0	49.1
Côte d'Ivoire	Assisted vaginal delivery	23.4	34.4	95.0	16.6
Côte d'Ivoire	Neonatal resuscitation	51.1	75.0	95.0	36.1
Côte d'Ivoire	Parenteral administration of uterotonics	83.1	95.0	95.0	58.7
Côte d'Ivoire	Removal of retained products of conception	30.7	45.1	95.0	21.7
Côte d'Ivoire	Induction of labour for post-term pregnancies	1.6	2.3	95.0	1.1
Côte d'Ivoire	Antenatal corticosteroids for preterm labour	66.4	95.0	95.0	47.0
Côte d'Ivoire	Breastfeeding promotion	28.9	42.4	95.0	20.4
Côte d'Ivoire	KMC - Kangaroo mother care	4.6	6.8	95.0	3.3
Côte d'Ivoire	Oral antibiotics for neonatal sepsis	0.0	0.0	0.0	0.0
Côte d'Ivoire	Injectable antibiotics for neonatal sepsis	92.9	95.0	95.0	65.7
DPR Korea	Folic acid supplementation/fortification	3.1	4.6	95.0	2.2
DPR Korea	Safe abortion services	0.0	0.0	95.0	0.0
DPR Korea	Post abortion case management	79.8	95.0	95.0	56.4
DPR Korea	Ectopic pregnancy case management	79.8	95.0	95.0	56.4
DPR Korea	TT - Tetanus toxoid vaccination	95.0	95.0	95.0	80.0
DPR Korea	IPTp	20.0	29.3	95.0	14.1
DPR Korea	Syphilis detection and treatment	46.5	68.2	95.0	32.8
DPR Korea	Iron supplementation in pregnancy	6.3	9.2	95.0	4.4
DPR Korea	Hypertensive disorder case management	6.1	9.0	95.0	4.3
DPR Korea	Diabetes case management	6.3	9.2	95.0	4.4
DPR Korea	Malaria case management	42.5	62.3	95.0	30.0
DPR Korea	MgSO4 management of pre-eclampsia	8.8	12.9	95.0	6.2
DPR Korea	Clean birth environment	54.4	79.9	95.0	38.5
DPR Korea	Immediate drying and additional stimulation	84.1	95.0	95.0	59.5
DPR Korea	Thermal protection	95.0	95.0	95.0	74.3
DPR Korea	Clean cord care	88.6	95.0	95.0	62.7
DPR Korea	Manual removal of placenta	28.2	41.4	95.0	20.0
DPR Korea	Parenteral administration of anti-convulsants	18.9	27.7	95.0	13.4
DPR Korea	Antibiotics for preterm or prolonged PROM	16.2	23.8	95.0	11.5
DPR Korea	Parenteral administration of antibiotics	16.2	23.8	95.0	11.5
DPR Korea	Assisted vaginal delivery	11.8	17.4	95.0	8.4
DPR Korea	Neonatal resuscitation	5.9	8.6	95.0	4.1
DPR Korea	Parenteral administration of uterotonics	81.6	95.0	95.0	57.7
DPR Korea	Removal of retained products of conception	26.5	38.9	95.0	18.7
DPR Korea	Induction of labour for post-term pregnancies	1.6	2.3	95.0	1.1
DPR Korea	Antenatal corticosteroids for preterm labour	18.9	27.7	95.0	13.4
DPR Korea	Breastfeeding promotion	58.8	86.3	95.0	41.6
DPR Korea	KMC - Kangaroo mother care	5.3	7.8	95.0	3.8
DPR Korea	Oral antibiotics for neonatal sepsis	0.0	0.0	0.0	0.0
DPR Korea	Injectable antibiotics for neonatal sepsis	95.0	95.0	95.0	75.2
DR Congo	Folic acid supplementation/fortification	6.2	9.2	95.0	4.4
DR Congo	Safe abortion services	100.0	100.0	100.0	94.1
DR Congo	Post abortion case management	92.0	95.0	95.0	65.1
DR Congo	Ectopic pregnancy case management	92.0	95.0	95.0	65.1
DR Congo	TT - Tetanus toxoid vaccination	98.0	98.0	98.0	92.2
DR Congo	IPTp	0.0	0.0	95.0	0.0
DR Congo	Syphilis detection and treatment	32.5	47.7	95.0	23.0
DR Congo	Iron supplementation in pregnancy	0.0	0.0	95.0	0.0
DR Congo	Hypertensive disorder case management	29.9	43.9	95.0	21.2
DR Congo	Diabetes case management	23.4	34.4	95.0	16.6
DR Congo	Malaria case management	95.0	95.0	95.0	68.3
DR Congo	MgSO4 management of pre-eclampsia	59.2	86.9	95.0	41.9
DR Congo	Clean birth environment	95.0	95.0	95.0	71.2

Country	Intervention	10% Increase	25% increase	95% target	2% Attrition
DR Congo	Immediate drying and additional stimulation	95.0	95.0	95.0	79.4
DR Congo	Thermal protection	95.0	95.0	95.0	85.7
DR Congo	Clean cord care	95.0	95.0	95.0	82.8
DR Congo	Manual removal of placenta	45.9	67.4	95.0	32.5
DR Congo	Parenteral administration of anti-convulsants	87.7	95.0	95.0	62.0
DR Congo	Antibiotics for preterm or prolonged PROM	91.8	95.0	95.0	64.9
DR Congo	Parenteral administration of antibiotics	91.8	95.0	95.0	64.9
DR Congo	Assisted vaginal delivery	31.0	45.5	95.0	21.9
DR Congo	Neonatal resuscitation	67.5	95.0	95.0	47.7
DR Congo	Parenteral administration of uterotonics	95.0	95.0	95.0	77.6
DR Congo	Removal of retained products of conception	40.7	59.8	95.0	28.8
DR Congo	Induction of labour for post-term pregnancies	2.1	3.1	95.0	1.5
DR Congo	Antenatal corticosteroids for preterm labour	87.7	95.0	95.0	62.0
DR Congo	Breastfeeding promotion	0.0	0.0	95.0	0.0
DR Congo	KMC - Kangaroo mother care	6.1	9.0	95.0	4.3
DR Congo	Oral antibiotics for neonatal sepsis	0.0	0.0	0.0	0.0
DR Congo	Injectable antibiotics for neonatal sepsis	95.0	95.0	95.0	86.8
Djibouti	Folic acid supplementation/fortification	1.5	2.2	95.0	1.1
Djibouti	Safe abortion services	4.4	6.4	95.0	3.1
Djibouti	Post abortion case management	87.2	95.0	95.0	61.7
Djibouti	Ectopic pregnancy case management	87.2	95.0	95.0	61.7
Djibouti	TT - Tetanus toxoid vaccination	98.0	98.0	98.0	92.2
Djibouti	IPTp	0.0	0.0	95.0	0.0
Djibouti	Syphilis detection and treatment	7.5	10.9	95.0	5.3
Djibouti	Iron supplementation in pregnancy	0.0	0.0	95.0	0.0
Djibouti	Hypertensive disorder case management	7.2	10.5	95.0	5.1
Djibouti	Diabetes case management	5.6	8.2	95.0	4.0
Djibouti	Malaria case management	23.3	34.2	95.0	16.5
Djibouti	MgSO4 management of pre-eclampsia	14.2	20.9	95.0	10.1
Djibouti	Clean birth environment	95.0	95.0	95.0	67.5
Djibouti	Immediate drying and additional stimulation	95.0	95.0	95.0	75.3
Djibouti	Thermal protection	95.0	95.0	95.0	81.3
Djibouti	Clean cord care	95.0	95.0	95.0	78.5
Djibouti	Manual removal of placenta	43.5	63.9	95.0	30.8
Djibouti	Parenteral administration of anti-convulsants	83.2	95.0	95.0	58.8
Djibouti	Antibiotics for preterm or prolonged PROM	87.0	95.0	95.0	61.6
Djibouti	Parenteral administration of antibiotics	87.0	95.0	95.0	61.6
Djibouti	Assisted vaginal delivery	29.4	43.2	95.0	20.8
Djibouti	Neonatal resuscitation	64.0	93.9	95.0	45.3
Djibouti	Parenteral administration of uterotonics	95.0	95.0	95.0	73.5
Djibouti	Removal of retained products of conception	38.6	56.6	95.0	27.3
Djibouti	Induction of labour for post-term pregnancies	2.0	2.9	95.0	1.4
Djibouti	Antenatal corticosteroids for preterm labour	83.2	95.0	95.0	58.8
Djibouti	Breastfeeding promotion	1.9	2.7	95.0	1.3
Djibouti	KMC - Kangaroo mother care	5.8	8.5	95.0	4.1
Djibouti	Oral antibiotics for neonatal sepsis	0.0	0.0	0.0	0.0
Djibouti	Injectable antibiotics for neonatal sepsis	95.0	95.0	95.0	82.3
Dominican Republic	Folic acid supplementation/fortification	6.2	9.1	95.0	4.4
Dominican Republic	Safe abortion services	71.5	95.0	95.0	50.5
Dominican Republic	Post abortion case management	95.0	95.0	95.0	69.1
Dominican Republic	Ectopic pregnancy case management	95.0	95.0	95.0	69.1
Dominican Republic	TT - Tetanus toxoid vaccination	95.0	95.0	99.0	93.2
Dominican Republic	IPTp	0.0	0.0	95.0	0.0
Dominican Republic	Syphilis detection and treatment	32.2	47.3	95.0	22.8
Dominican Republic	Iron supplementation in pregnancy	95.0	95.0	95.0	76.7
Dominican Republic	Hypertensive disorder case management	29.7	43.6	95.0	21.0
Dominican Republic	Diabetes case management	23.2	34.0	95.0	16.4
Dominican Republic	Malaria case management	95.0	95.0	95.0	67.8
Dominican Republic	MgSO4 management of pre-eclampsia	58.8	86.3	95.0	41.6
Dominican Republic	Clean birth environment	95.0	95.0	95.0	75.6
Dominican Republic	Immediate drying and additional stimulation	95.0	95.0	95.0	84.3
Dominican Republic	Thermal protection	96.7	96.7	96.7	91.0
Dominican Republic	Clean cord care	95.0	95.0	95.0	87.9
Dominican Republic	Manual removal of placenta	48.7	71.5	95.0	34.4
Dominican Republic	Parenteral administration of anti-convulsants	93.2	95.0	95.0	65.9

Country	Intervention	10% Increase	25% increase	95% target	2% Attrition
Dominican Republic	Antibiotics for preterm or prolonged PROM	95.0	95.0	95.0	68.9
Dominican Republic	Parenteral administration of antibiotics	95.0	95.0	95.0	68.9
Dominican Republic	Assisted vaginal delivery	32.9	48.2	95.0	23.2
Dominican Republic	Neonatal resuscitation	71.6	95.0	95.0	50.6
Dominican Republic	Parenteral administration of uterotonics	95.0	95.0	95.0	82.4
Dominican Republic	Removal of retained products of conception	43.1	63.3	95.0	30.5
Dominican Republic	Induction of labour for post-term pregnancies	2.3	3.3	95.0	1.6
Dominican Republic	Antenatal corticosteroids for preterm labour	93.2	95.0	95.0	65.9
Dominican Republic	Breastfeeding promotion	6.1	9.0	95.0	4.3
Dominican Republic	KMC - Kangaroo mother care	6.5	9.6	95.0	4.6
Dominican Republic	Oral antibiotics for neonatal sepsis	0.0	0.0	0.0	0.0
Dominican Republic	Injectable antibiotics for neonatal sepsis	97.9	97.9	97.9	92.1
Egypt	Folic acid supplementation/fortification	5.6	8.2	95.0	3.9
Egypt	Safe abortion services	2.9	4.3	95.0	2.1
Egypt	Post abortion case management	86.5	95.0	95.0	61.2
Egypt	Ectopic pregnancy case management	86.5	95.0	95.0	61.2
Egypt	TT - Tetanus toxoid vaccination	30.1	44.1	95.0	21.3
Egypt	IPTp	0.0	0.0	95.0	0.0
Egypt	Syphilis detection and treatment	48.0	70.5	95.0	34.0
Egypt	Iron supplementation in pregnancy	0.0	0.0	95.0	0.0
Egypt	Hypertensive disorder case management	86.5	95.0	95.0	61.2
Egypt	Diabetes case management	53.0	77.7	95.0	37.5
Egypt	Malaria case management	0.0	0.0	95.0	0.0
Egypt	MgSO4 management of pre-eclampsia	0.0	0.0	95.0	0.0
Egypt	Clean birth environment	94.6	95.0	95.0	66.9
Egypt	Immediate drying and additional stimulation	95.0	95.0	95.0	74.7
Egypt	Thermal protection	95.0	95.0	95.0	80.7
Egypt	Clean cord care	95.0	95.0	95.0	77.9
Egypt	Manual removal of placenta	43.1	63.3	95.0	30.5
Egypt	Parenteral administration of anti-convulsants	82.5	95.0	95.0	58.4
Egypt	Antibiotics for preterm or prolonged PROM	86.4	95.0	95.0	61.1
Egypt	Parenteral administration of antibiotics	86.4	95.0	95.0	61.1
Egypt	Assisted vaginal delivery	29.1	42.8	95.0	20.6
Egypt	Neonatal resuscitation	63.5	93.2	95.0	44.9
Egypt	Parenteral administration of uterotonics	95.0	95.0	95.0	72.9
Egypt	Removal of retained products of conception	38.2	56.1	95.0	27.0
Egypt	Induction of labour for post-term pregnancies	2.0	2.9	95.0	1.4
Egypt	Antenatal corticosteroids for preterm labour	82.5	95.0	95.0	58.4
Egypt	Breastfeeding promotion	47.0	68.9	95.0	33.2
Egypt	KMC - Kangaroo mother care	5.8	8.5	95.0	4.1
Egypt	Oral antibiotics for neonatal sepsis	0.0	0.0	0.0	0.0
Egypt	Injectable antibiotics for neonatal sepsis	95.0	95.0	95.0	81.6
Equatorial Guinea	Folic acid supplementation/fortification	4.5	6.5	95.0	3.1
Equatorial Guinea	Safe abortion services	0.0	0.0	95.0	0.0
Equatorial Guinea	Post abortion case management	67.2	95.0	95.0	47.5
Equatorial Guinea	Ectopic pregnancy case management	67.2	95.0	95.0	47.5
Equatorial Guinea	TT - Tetanus toxoid vaccination	93.2	95.0	95.0	65.9
Equatorial Guinea	IPTp	0.0	0.0	95.0	0.0
Equatorial Guinea	Syphilis detection and treatment	29.1	42.8	95.0	20.6
Equatorial Guinea	Iron supplementation in pregnancy	11.6	17.0	95.0	8.2
Equatorial Guinea	Hypertensive disorder case management	21.4	31.4	95.0	15.2
Equatorial Guinea	Diabetes case management	16.6	24.4	95.0	11.8
Equatorial Guinea	Malaria case management	69.1	95.0	95.0	48.8
Equatorial Guinea	MgSO4 management of pre-eclampsia	42.3	62.1	95.0	29.9
Equatorial Guinea	Clean birth environment	73.5	95.0	95.0	52.0
Equatorial Guinea	Immediate drying and additional stimulation	82.0	95.0	95.0	58.0
Equatorial Guinea	Thermal protection	88.5	95.0	95.0	62.6
Equatorial Guinea	Clean cord care	85.5	95.0	95.0	60.4
Equatorial Guinea	Manual removal of placenta	33.5	49.2	95.0	23.7
Equatorial Guinea	Parenteral administration of anti-convulsants	64.0	93.9	95.0	45.3
Equatorial Guinea	Antibiotics for preterm or prolonged PROM	67.1	95.0	95.0	47.4
Equatorial Guinea	Parenteral administration of antibiotics	67.1	95.0	95.0	47.4
Equatorial Guinea	Assisted vaginal delivery	22.6	33.2	95.0	16.0
Equatorial Guinea	Neonatal resuscitation	49.2	72.3	95.0	34.8
Equatorial Guinea	Parenteral administration of uterotonics	80.0	95.0	95.0	56.6

Country	Intervention	10% Increase	25% increase	95% target	2% Attrition
Equatorial Guinea	Removal of retained products of conception	29.7	43.6	95.0	21.0
Equatorial Guinea	Induction of labour for post-term pregnancies	1.6	2.3	95.0	1.1
Equatorial Guinea	Antenatal corticosteroids for preterm labour	64.0	93.9	95.0	45.3
Equatorial Guinea	Breastfeeding promotion	0.0	0.0	95.0	0.0
Equatorial Guinea	KMC - Kangaroo mother care	4.5	6.6	95.0	3.2
Equatorial Guinea	Oral antibiotics for neonatal sepsis	0.0	0.0	0.0	0.0
Equatorial Guinea	Injectable antibiotics for neonatal sepsis	89.6	95.0	95.0	63.3
Eritrea	Folic acid supplementation/fortification	3.8	5.6	95.0	2.7
Eritrea	Safe abortion services	4.4	6.4	95.0	3.1
Eritrea	Post abortion case management	22.4	32.8	95.0	15.8
Eritrea	Ectopic pregnancy case management	22.4	32.8	95.0	15.8
Eritrea	TT - Tetanus toxoid vaccination	99.0	99.0	99.0	93.2
Eritrea	IPTp	0.0	0.0	95.0	0.0
Eritrea	Syphilis detection and treatment	23.3	34.2	95.0	16.5
Eritrea	Iron supplementation in pregnancy	0.0	0.0	95.0	0.0
Eritrea	Hypertensive disorder case management	18.4	27.0	95.0	13.0
Eritrea	Diabetes case management	14.4	21.1	95.0	10.2
Eritrea	Malaria case management	59.2	86.9	95.0	41.9
Eritrea	MgSO4 management of pre-eclampsia	36.3	53.3	95.0	25.7
Eritrea	Clean birth environment	36.7	53.9	95.0	26.0
Eritrea	Immediate drying and additional stimulation	41.0	60.2	95.0	29.0
Eritrea	Thermal protection	44.2	64.8	95.0	31.2
Eritrea	Clean cord care	42.7	62.7	95.0	30.2
Eritrea	Manual removal of placenta	16.8	24.6	95.0	11.9
Eritrea	Parenteral administration of anti-convulsants	31.9	46.9	95.0	22.6
Eritrea	Antibiotics for preterm or prolonged PROM	33.4	49.0	95.0	23.6
Eritrea	Parenteral administration of antibiotics	33.4	49.0	95.0	23.6
Eritrea	Assisted vaginal delivery	11.3	16.6	95.0	8.0
Eritrea	Neonatal resuscitation	24.6	36.1	95.0	17.4
Eritrea	Parenteral administration of uterotonics	39.9	58.6	95.0	28.2
Eritrea	Removal of retained products of conception	14.8	21.7	95.0	10.4
Eritrea	Induction of labour for post-term pregnancies	0.8	1.2	95.0	0.6
Eritrea	Antenatal corticosteroids for preterm labour	31.9	46.9	95.0	22.6
Eritrea	Breastfeeding promotion	34.3	50.4	95.0	24.3
Eritrea	KMC - Kangaroo mother care	2.2	3.3	95.0	1.6
Eritrea	Oral antibiotics for neonatal sepsis	0.0	0.0	0.0	0.0
Eritrea	Injectable antibiotics for neonatal sepsis	44.7	65.6	95.0	31.6
Eswatini	Folic acid supplementation/fortification	5.1	7.4	95.0	3.6
Eswatini	Safe abortion services	56.6	83.0	95.0	40.0
Eswatini	Post abortion case management	87.5	95.0	95.0	61.9
Eswatini	Ectopic pregnancy case management	87.5	95.0	95.0	61.9
Eswatini	TT - Tetanus toxoid vaccination	95.0	95.0	95.0	82.8
Eswatini	IPTp	0.0	0.0	95.0	0.0
Eswatini	Syphilis detection and treatment	32.5	47.7	95.0	23.0
Eswatini	Iron supplementation in pregnancy	44.7	65.6	95.0	31.6
Eswatini	Hypertensive disorder case management	24.4	35.7	95.0	17.2
Eswatini	Diabetes case management	19.0	27.9	95.0	13.5
Eswatini	Malaria case management	78.5	95.0	95.0	55.5
Eswatini	MgSO4 management of pre-eclampsia	48.2	70.7	95.0	34.1
Eswatini	Clean birth environment	95.0	95.0	95.0	67.7
Eswatini	Immediate drying and additional stimulation	95.0	95.0	95.0	75.6
Eswatini	Thermal protection	95.0	95.0	95.0	81.6
Eswatini	Clean cord care	95.0	95.0	95.0	78.8
Eswatini	Manual removal of placenta	43.7	64.1	95.0	30.9
Eswatini	Parenteral administration of anti-convulsants	83.5	95.0	95.0	59.0
Eswatini	Antibiotics for preterm or prolonged PROM	87.3	95.0	95.0	61.7
Eswatini	Parenteral administration of antibiotics	87.3	95.0	95.0	61.7
Eswatini	Assisted vaginal delivery	29.5	43.4	95.0	20.9
Eswatini	Neonatal resuscitation	64.2	94.1	95.0	45.4
Eswatini	Parenteral administration of uterotonics	95.0	95.0	95.0	73.8
Eswatini	Removal of retained products of conception	38.7	56.8	95.0	27.4
Eswatini	Induction of labour for post-term pregnancies	2.0	2.9	95.0	1.4
Eswatini	Antenatal corticosteroids for preterm labour	83.5	95.0	95.0	59.0
Eswatini	Breastfeeding promotion	82.0	95.0	95.0	58.0
Eswatini	KMC - Kangaroo mother care	5.8	8.6	95.0	4.1

Country	Intervention	10% Increase	25% increase	95% target	2% Attrition
Eswatini	Oral antibiotics for neonatal sepsis	0.0	0.0	0.0	0.0
Eswatini	Injectable antibiotics for neonatal sepsis	95.0	95.0	95.0	82.5
Ethiopia	Folic acid supplementation/fortification	2.2	3.2	95.0	1.5
Ethiopia	Safe abortion services	4.4	6.4	95.0	3.1
Ethiopia	Post abortion case management	3.5	5.1	95.0	2.5
Ethiopia	Ectopic pregnancy case management	3.5	5.1	95.0	2.5
Ethiopia	TT - Tetanus toxoid vaccination	95.0	95.0	95.0	87.5
Ethiopia	IPTp	0.0	0.0	95.0	0.0
Ethiopia	Syphilis detection and treatment	20.9	30.7	95.0	14.8
Ethiopia	Iron supplementation in pregnancy	6.8	10.0	95.0	4.8
Ethiopia	Hypertensive disorder case management	10.5	15.4	95.0	7.4
Ethiopia	Diabetes case management	8.1	11.9	95.0	5.7
Ethiopia	Malaria case management	33.8	49.6	95.0	23.9
Ethiopia	MgSO4 management of pre-eclampsia	20.8	30.5	95.0	14.7
Ethiopia	Clean birth environment	28.6	42.0	95.0	20.2
Ethiopia	Immediate drying and additional stimulation	31.9	46.9	95.0	22.6
Ethiopia	Thermal protection	34.5	50.6	95.0	24.4
Ethiopia	Clean cord care	33.3	48.8	95.0	23.5
Ethiopia	Manual removal of placenta	13.0	19.1	95.0	9.2
Ethiopia	Parenteral administration of anti-convulsants	25.0	36.7	95.0	17.7
Ethiopia	Antibiotics for preterm or prolonged PROM	26.1	38.3	95.0	18.4
Ethiopia	Parenteral administration of antibiotics	26.1	38.3	95.0	18.4
Ethiopia	Assisted vaginal delivery	8.8	12.9	95.0	6.2
Ethiopia	Neonatal resuscitation	19.2	28.1	95.0	13.6
Ethiopia	Parenteral administration of uterotonics	31.1	45.7	95.0	22.0
Ethiopia	Removal of retained products of conception	11.6	17.0	95.0	8.2
Ethiopia	Induction of labour for post-term pregnancies	0.7	1.0	95.0	0.5
Ethiopia	Antenatal corticosteroids for preterm labour	25.0	36.7	95.0	17.7
Ethiopia	Breastfeeding promotion	0.0	0.0	95.0	0.0
Ethiopia	KMC - Kangaroo mother care	1.7	2.6	95.0	1.2
Ethiopia	Oral antibiotics for neonatal sepsis	0.0	0.0	0.0	0.0
Ethiopia	Injectable antibiotics for neonatal sepsis	34.9	51.2	95.0	24.7
Gabon	Folic acid supplementation/fortification	5.1	7.5	95.0	3.6
Gabon	Safe abortion services	0.0	0.0	95.0	0.0
Gabon	Post abortion case management	90.0	95.0	95.0	63.7
Gabon	Ectopic pregnancy case management	90.0	95.0	95.0	63.7
Gabon	TT - Tetanus toxoid vaccination	95.0	95.0	95.0	80.0
Gabon	IPTp	17.4	25.6	95.0	12.3
Gabon	Syphilis detection and treatment	31.1	45.7	95.0	22.0
Gabon	Iron supplementation in pregnancy	75.6	95.0	95.0	53.5
Gabon	Hypertensive disorder case management	24.6	36.1	95.0	17.4
Gabon	Diabetes case management	19.3	28.3	95.0	13.6
Gabon	Malaria case management	79.6	95.0	95.0	56.3
Gabon	MgSO4 management of pre-eclampsia	48.8	71.7	95.0	34.5
Gabon	Clean birth environment	95.0	95.0	95.0	69.6
Gabon	Immediate drying and additional stimulation	95.0	95.0	95.0	77.7
Gabon	Thermal protection	95.0	95.0	95.0	84.0
Gabon	Clean cord care	95.0	95.0	95.0	81.0
Gabon	Manual removal of placenta	44.9	65.8	95.0	31.7
Gabon	Parenteral administration of anti-convulsants	85.8	95.0	95.0	60.7
Gabon	Antibiotics for preterm or prolonged PROM	89.8	95.0	95.0	63.5
Gabon	Parenteral administration of antibiotics	89.8	95.0	95.0	63.5
Gabon	Assisted vaginal delivery	30.3	44.5	95.0	21.5
Gabon	Neonatal resuscitation	66.0	95.0	95.0	46.7
Gabon	Parenteral administration of uterotonics	95.0	95.0	95.0	75.9
Gabon	Removal of retained products of conception	39.8	58.4	95.0	28.1
Gabon	Induction of labour for post-term pregnancies	2.1	3.1	95.0	1.5
Gabon	Antenatal corticosteroids for preterm labour	85.8	95.0	95.0	60.7
Gabon	Breastfeeding promotion	6.0	8.8	95.0	4.2
Gabon	KMC - Kangaroo mother care	6.0	8.8	95.0	4.2
Gabon	Oral antibiotics for neonatal sepsis	0.0	0.0	0.0	0.0
Gabon	Injectable antibiotics for neonatal sepsis	95.0	95.0	95.0	84.9
Gambia	Folic acid supplementation/fortification	5.1	7.5	95.0	3.6
Gambia	Safe abortion services	0.0	0.0	95.0	0.0
Gambia	Post abortion case management	57.7	84.7	95.0	40.8

Country	Intervention	10% Increase	25% increase	95% target	2% Attrition
Gambia	Ectopic pregnancy case management	57.7	84.7	95.0	40.8
Gambia	TT - Tetanus toxoid vaccination	95.0	95.0	95.0	86.6
Gambia	IPTp	83.9	95.0	95.0	59.3
Gambia	Syphilis detection and treatment	32.5	47.7	95.0	23.0
Gambia	Iron supplementation in pregnancy	59.4	87.1	95.0	42.0
Gambia	Hypertensive disorder case management	24.5	35.9	95.0	17.3
Gambia	Diabetes case management	19.2	28.1	95.0	13.6
Gambia	Malaria case management	79.1	95.0	95.0	55.9
Gambia	MgSO4 management of pre-eclampsia	48.6	71.3	95.0	34.4
Gambia	Clean birth environment	63.1	92.6	95.0	44.6
Gambia	Immediate drying and additional stimulation	70.4	95.0	95.0	49.8
Gambia	Thermal protection	76.1	95.0	95.0	53.8
Gambia	Clean cord care	73.5	95.0	95.0	52.0
Gambia	Manual removal of placenta	28.7	42.2	95.0	20.3
Gambia	Parenteral administration of anti-convulsants	55.1	80.9	95.0	39.0
Gambia	Antibiotics for preterm or prolonged PROM	57.6	84.6	95.0	40.8
Gambia	Parenteral administration of antibiotics	57.6	84.6	95.0	40.8
Gambia	Assisted vaginal delivery	19.4	28.5	95.0	13.7
Gambia	Neonatal resuscitation	42.3	62.1	95.0	29.9
Gambia	Parenteral administration of uterotonics	68.8	95.0	95.0	48.7
Gambia	Removal of retained products of conception	25.6	37.5	95.0	18.1
Gambia	Induction of labour for post-term pregnancies	1.3	2.0	95.0	0.9
Gambia	Antenatal corticosteroids for preterm labour	55.1	80.9	95.0	39.0
Gambia	Breastfeeding promotion	59.6	87.5	95.0	42.2
Gambia	KMC - Kangaroo mother care	3.8	5.6	95.0	2.7
Gambia	Oral antibiotics for neonatal sepsis	0.0	0.0	0.0	0.0
Gambia	Injectable antibiotics for neonatal sepsis	76.9	95.0	95.0	54.4
Ghana	Folic acid supplementation/fortification	5.9	8.7	95.0	4.2
Ghana	Safe abortion services	0.0	0.0	95.0	0.0
Ghana	Post abortion case management	78.6	95.0	95.0	55.6
Ghana	Ectopic pregnancy case management	78.6	95.0	95.0	55.6
Ghana	TT - Tetanus toxoid vaccination	95.0	95.0	95.0	83.8
Ghana	IPTp	95.0	95.0	95.0	73.4
Ghana	Syphilis detection and treatment	32.1	47.1	95.0	22.7
Ghana	Iron supplementation in pregnancy	79.1	95.0	95.0	55.9
Ghana	Hypertensive disorder case management	28.6	42.0	95.0	20.2
Ghana	Diabetes case management	22.2	32.6	95.0	15.7
Ghana	Malaria case management	92.1	95.0	95.0	65.1
Ghana	MgSO4 management of pre-eclampsia	56.4	82.8	95.0	39.9
Ghana	Clean birth environment	85.8	95.0	95.0	60.7
Ghana	Immediate drying and additional stimulation	95.0	95.0	95.0	67.9
Ghana	Thermal protection	95.0	95.0	95.0	73.2
Ghana	Clean cord care	95.0	95.0	95.0	70.7
Ghana	Manual removal of placenta	39.1	57.4	95.0	27.7
Ghana	Parenteral administration of anti-convulsants	74.9	95.0	95.0	53.0
Ghana	Antibiotics for preterm or prolonged PROM	78.4	95.0	95.0	55.4
Ghana	Parenteral administration of antibiotics	78.4	95.0	95.0	55.4
Ghana	Assisted vaginal delivery	26.5	38.9	95.0	18.7
Ghana	Neonatal resuscitation	57.6	84.6	95.0	40.8
Ghana	Parenteral administration of uterotonics	93.6	95.0	95.0	66.2
Ghana	Removal of retained products of conception	34.7	51.0	95.0	24.6
Ghana	Induction of labour for post-term pregnancies	1.9	2.7	95.0	1.3
Ghana	Antenatal corticosteroids for preterm labour	74.9	95.0	95.0	53.0
Ghana	Breastfeeding promotion	66.6	95.0	95.0	47.1
Ghana	KMC - Kangaroo mother care	5.2	7.7	95.0	3.7
Ghana	Oral antibiotics for neonatal sepsis	0.0	0.0	0.0	0.0
Ghana	Injectable antibiotics for neonatal sepsis	95.0	95.0	95.0	74.1
Guatemala	Folic acid supplementation/fortification	5.7	8.4	95.0	4.0
Guatemala	Safe abortion services	0.8	1.2	95.0	0.6
Guatemala	Post abortion case management	64.9	95.0	95.0	45.9
Guatemala	Ectopic pregnancy case management	64.9	95.0	95.0	45.9
Guatemala	TT - Tetanus toxoid vaccination	95.0	95.0	95.0	84.7
Guatemala	IPTp	0.0	0.0	95.0	0.0
Guatemala	Syphilis detection and treatment	30.1	44.1	95.0	21.3
Guatemala	Iron supplementation in pregnancy	39.0	57.2	95.0	27.6

Country	Intervention	10% Increase	25% increase	95% target	2% Attrition
Guatemala	Hypertensive disorder case management	27.4	40.2	95.0	19.4
Guatemala	Diabetes case management	21.4	31.4	95.0	15.2
Guatemala	Malaria case management	88.6	95.0	95.0	62.7
Guatemala	MgSO4 management of pre-eclampsia	54.3	79.7	95.0	38.4
Guatemala	Clean birth environment	70.9	95.0	95.0	50.2
Guatemala	Immediate drying and additional stimulation	79.2	95.0	95.0	56.0
Guatemala	Thermal protection	85.5	95.0	95.0	60.4
Guatemala	Clean cord care	82.5	95.0	95.0	58.4
Guatemala	Manual removal of placenta	32.3	47.5	95.0	22.9
Guatemala	Parenteral administration of anti-convulsants	61.9	90.8	95.0	43.8
Guatemala	Antibiotics for preterm or prolonged PROM	64.7	94.9	95.0	45.7
Guatemala	Parenteral administration of antibiotics	64.7	94.9	95.0	45.7
Guatemala	Assisted vaginal delivery	21.8	32.0	95.0	15.4
Guatemala	Neonatal resuscitation	47.5	69.7	95.0	33.6
Guatemala	Parenteral administration of uterotonics	77.3	95.0	95.0	54.7
Guatemala	Removal of retained products of conception	28.6	42.0	95.0	20.2
Guatemala	Induction of labour for post-term pregnancies	1.5	2.1	95.0	1.0
Guatemala	Antenatal corticosteroids for preterm labour	61.9	90.8	95.0	43.8
Guatemala	Breastfeeding promotion	69.2	95.0	95.0	48.9
Guatemala	KMC - Kangaroo mother care	4.3	6.3	95.0	3.1
Guatemala	Oral antibiotics for neonatal sepsis	0.0	0.0	0.0	0.0
Guatemala	Injectable antibiotics for neonatal sepsis	86.5	95.0	95.0	61.2
Guinea	Folic acid supplementation/fortification	3.3	4.9	95.0	2.4
Guinea	Safe abortion services	0.0	0.0	95.0	0.0
Guinea	Post abortion case management	57.4	84.2	95.0	40.6
Guinea	Ectopic pregnancy case management	57.4	84.2	95.0	40.6
Guinea	TT - Tetanus toxoid vaccination	95.0	95.0	95.0	75.3
Guinea	IPTp	56.7	83.2	95.0	40.1
Guinea	Syphilis detection and treatment	22.9	33.6	95.0	16.2
Guinea	Iron supplementation in pregnancy	55.2	81.1	95.0	39.1
Guinea	Hypertensive disorder case management	16.1	23.6	95.0	11.4
Guinea	Diabetes case management	12.5	18.4	95.0	8.8
Guinea	Malaria case management	51.8	76.0	95.0	36.6
Guinea	MgSO4 management of pre-eclampsia	31.8	46.7	95.0	22.5
Guinea	Clean birth environment	62.7	92.0	95.0	44.3
Guinea	Immediate drying and additional stimulation	70.0	95.0	95.0	49.5
Guinea	Thermal protection	75.6	95.0	95.0	53.5
Guinea	Clean cord care	72.9	95.0	95.0	51.6
Guinea	Manual removal of placenta	28.6	42.0	95.0	20.2
Guinea	Parenteral administration of anti-convulsants	54.7	80.3	95.0	38.7
Guinea	Antibiotics for preterm or prolonged PROM	57.2	84.0	95.0	40.5
Guinea	Parenteral administration of antibiotics	57.2	84.0	95.0	40.5
Guinea	Assisted vaginal delivery	19.3	28.3	95.0	13.6
Guinea	Neonatal resuscitation	42.1	61.7	95.0	29.7
Guinea	Parenteral administration of uterotonics	68.4	95.0	95.0	48.4
Guinea	Removal of retained products of conception	25.3	37.1	95.0	17.9
Guinea	Induction of labour for post-term pregnancies	1.3	2.0	95.0	0.9
Guinea	Antenatal corticosteroids for preterm labour	54.7	80.3	95.0	38.7
Guinea	Breastfeeding promotion	0.0	0.0	95.0	0.0
Guinea	KMC - Kangaroo mother care	3.8	5.6	95.0	2.7
Guinea	Oral antibiotics for neonatal sepsis	0.0	0.0	0.0	0.0
Guinea	Injectable antibiotics for neonatal sepsis	76.5	95.0	95.0	54.1
Guinea-Bissau	Folic acid supplementation/fortification	4.3	6.3	95.0	3.1
Guinea-Bissau	Safe abortion services	0.0	0.0	95.0	0.0
Guinea-Bissau	Post abortion case management	29.3	43.0	95.0	20.7
Guinea-Bissau	Ectopic pregnancy case management	29.3	43.0	95.0	20.7
Guinea-Bissau	TT - Tetanus toxoid vaccination	95.0	95.0	95.0	78.1
Guinea-Bissau	IPTp	60.6	88.9	95.0	42.8
Guinea-Bissau	Syphilis detection and treatment	30.5	44.7	95.0	21.6
Guinea-Bissau	Iron supplementation in pregnancy	0.0	0.0	95.0	0.0
Guinea-Bissau	Hypertensive disorder case management	20.8	30.5	95.0	14.7
Guinea-Bissau	Diabetes case management	16.2	23.8	95.0	11.5
Guinea-Bissau	Malaria case management	66.9	95.0	95.0	47.3
Guinea-Bissau	MgSO4 management of pre-eclampsia	41.0	60.2	95.0	29.0
Guinea-Bissau	Clean birth environment	48.0	70.5	95.0	34.0

Country	Intervention	10% Increase	25% increase	95% target	2% Attrition
Guinea-Bissau	Immediate drying and additional stimulation	53.6	78.7	95.0	37.9
Guinea-Bissau	Thermal protection	57.9	85.0	95.0	40.9
Guinea-Bissau	Clean cord care	55.9	82.0	95.0	39.5
Guinea-Bissau	Manual removal of placenta	21.8	32.0	95.0	15.4
Guinea-Bissau	Parenteral administration of anti-convulsants	41.8	61.3	95.0	29.6
Guinea-Bissau	Antibiotics for preterm or prolonged PROM	43.8	64.3	95.0	31.0
Guinea-Bissau	Parenteral administration of antibiotics	43.8	64.3	95.0	31.0
Guinea-Bissau	Assisted vaginal delivery	14.8	21.7	95.0	10.4
Guinea-Bissau	Neonatal resuscitation	32.2	47.3	95.0	22.8
Guinea-Bissau	Parenteral administration of uterotonics	52.3	76.8	95.0	37.0
Guinea-Bissau	Removal of retained products of conception	19.4	28.5	95.0	13.7
Guinea-Bissau	Induction of labour for post-term pregnancies	1.1	1.6	95.0	0.8
Guinea-Bissau	Antenatal corticosteroids for preterm labour	41.8	61.3	95.0	29.6
Guinea-Bissau	Breastfeeding promotion	65.9	95.0	95.0	46.6
Guinea-Bissau	KMC - Kangaroo mother care	2.9	4.3	95.0	2.1
Guinea-Bissau	Oral antibiotics for neonatal sepsis	0.0	0.0	0.0	0.0
Guinea-Bissau	Injectable antibiotics for neonatal sepsis	58.6	85.9	95.0	41.4
Guyana	Folic acid supplementation/fortification	5.8	8.5	95.0	4.1
Guyana	Safe abortion services	0.3	0.4	95.0	0.2
Guyana	Post abortion case management	92.2	95.0	95.0	65.2
Guyana	Ectopic pregnancy case management	92.2	95.0	95.0	65.2
Guyana	TT - Tetanus toxoid vaccination	99.0	99.0	99.0	93.2
Guyana	IPTp	0.0	0.0	95.0	0.0
Guyana	Syphilis detection and treatment	29.8	43.8	95.0	21.1
Guyana	Iron supplementation in pregnancy	45.5	66.8	95.0	32.2
Guyana	Hypertensive disorder case management	27.7	40.6	95.0	19.6
Guyana	Diabetes case management	21.6	31.6	95.0	15.2
Guyana	Malaria case management	89.4	95.0	95.0	63.2
Guyana	MgSO4 management of pre-eclampsia	54.8	80.5	95.0	38.8
Guyana	Clean birth environment	95.0	95.0	95.0	71.3
Guyana	Immediate drying and additional stimulation	95.0	95.0	95.0	79.6
Guyana	Thermal protection	95.0	95.0	95.0	86.0
Guyana	Clean cord care	95.0	95.0	95.0	83.0
Guyana	Manual removal of placenta	46.1	67.6	95.0	32.6
Guyana	Parenteral administration of anti-convulsants	88.0	95.0	95.0	62.2
Guyana	Antibiotics for preterm or prolonged PROM	92.1	95.0	95.0	65.1
Guyana	Parenteral administration of antibiotics	92.1	95.0	95.0	65.1
Guyana	Assisted vaginal delivery	31.1	45.7	95.0	22.0
Guyana	Neonatal resuscitation	67.6	95.0	95.0	47.8
Guyana	Parenteral administration of uterotonics	95.0	95.0	95.0	77.7
Guyana	Removal of retained products of conception	40.7	59.8	95.0	28.8
Guyana	Induction of labour for post-term pregnancies	2.1	3.1	95.0	1.5
Guyana	Antenatal corticosteroids for preterm labour	88.0	95.0	95.0	62.2
Guyana	Breastfeeding promotion	29.8	43.8	95.0	21.1
Guyana	KMC - Kangaroo mother care	6.1	9.0	95.0	4.3
Guyana	Oral antibiotics for neonatal sepsis	0.0	0.0	0.0	0.0
Guyana	Injectable antibiotics for neonatal sepsis	95.0	95.0	95.0	87.0
Haiti	Folic acid supplementation/fortification	4.3	6.3	95.0	3.0
Haiti	Safe abortion services	71.5	95.0	95.0	50.5
Haiti	Post abortion case management	26.2	38.5	95.0	18.5
Haiti	Ectopic pregnancy case management	26.2	38.5	95.0	18.5
Haiti	TT - Tetanus toxoid vaccination	95.0	95.0	95.0	76.2
Haiti	IPTp	0.0	0.0	95.0	0.0
Haiti	Syphilis detection and treatment	25.8	37.9	95.0	18.3
Haiti	Iron supplementation in pregnancy	57.5	84.4	95.0	40.7
Haiti	Hypertensive disorder case management	20.6	30.3	95.0	14.6
Haiti	Diabetes case management	20.0	29.3	95.0	14.1
Haiti	Malaria case management	43.7	64.1	95.0	30.9
Haiti	MgSO4 management of pre-eclampsia	22.8	33.4	95.0	16.1
Haiti	Clean birth environment	45.0	66.0	95.0	31.8
Haiti	Immediate drying and additional stimulation	43.4	63.7	95.0	30.7
Haiti	Thermal protection	50.3	73.8	95.0	35.6
Haiti	Clean cord care	50.8	74.6	95.0	36.0
Haiti	Manual removal of placenta	19.7	28.9	95.0	13.9
Haiti	Parenteral administration of anti-convulsants	37.5	55.1	95.0	26.5

Country	Intervention	10% Increase	25% increase	95% target	2% Attrition
Haiti	Antibiotics for preterm or prolonged PROM	28.5	41.8	95.0	20.1
Haiti	Parenteral administration of antibiotics	28.5	41.8	95.0	20.1
Haiti	Assisted vaginal delivery	10.2	15.0	95.0	7.2
Haiti	Neonatal resuscitation	19.8	29.1	95.0	14.0
Haiti	Parenteral administration of uterotonics	33.9	49.8	95.0	24.0
Haiti	Removal of retained products of conception	14.6	21.5	95.0	10.4
Haiti	Induction of labour for post-term pregnancies	0.8	1.2	95.0	0.6
Haiti	Antenatal corticosteroids for preterm labour	37.5	55.1	95.0	26.5
Haiti	Breastfeeding promotion	40.3	59.2	95.0	28.5
Haiti	KMC - Kangaroo mother care	2.6	3.8	95.0	1.9
Haiti	Oral antibiotics for neonatal sepsis	0.0	0.0	0.0	0.0
Haiti	Injectable antibiotics for neonatal sepsis	52.4	77.0	95.0	37.1
Honduras	Folic acid supplementation/fortification	5.9	8.6	95.0	4.2
Honduras	Safe abortion services	0.8	1.2	95.0	0.6
Honduras	Post abortion case management	82.6	95.0	95.0	58.4
Honduras	Ectopic pregnancy case management	82.6	95.0	95.0	58.4
Honduras	TT - Tetanus toxoid vaccination	95.0	95.0	99.0	93.2
Honduras	IPTp	0.0	0.0	95.0	0.0
Honduras	Syphilis detection and treatment	31.8	46.7	95.0	22.5
Honduras	Iron supplementation in pregnancy	49.8	73.0	95.0	35.2
Honduras	Hypertensive disorder case management	28.2	41.4	95.0	20.0
Honduras	Diabetes case management	22.1	32.4	95.0	15.6
Honduras	Malaria case management	91.2	95.0	95.0	64.5
Honduras	MgSO4 management of pre-eclampsia	55.9	82.0	95.0	39.5
Honduras	Clean birth environment	90.4	95.0	95.0	63.9
Honduras	Immediate drying and additional stimulation	95.0	95.0	95.0	71.3
Honduras	Thermal protection	95.0	95.0	95.0	77.0
Honduras	Clean cord care	95.0	95.0	95.0	74.4
Honduras	Manual removal of placenta	41.1	60.4	95.0	29.1
Honduras	Parenteral administration of anti-convulsants	78.8	95.0	95.0	55.7
Honduras	Antibiotics for preterm or prolonged PROM	82.4	95.0	95.0	58.3
Honduras	Parenteral administration of antibiotics	82.4	95.0	95.0	58.3
Honduras	Assisted vaginal delivery	27.8	40.8	95.0	19.7
Honduras	Neonatal resuscitation	60.6	88.9	95.0	42.8
Honduras	Parenteral administration of uterotonics	95.0	95.0	95.0	69.6
Honduras	Removal of retained products of conception	36.5	53.5	95.0	25.8
Honduras	Induction of labour for post-term pregnancies	2.0	2.9	95.0	1.4
Honduras	Antenatal corticosteroids for preterm labour	78.8	95.0	95.0	55.7
Honduras	Breastfeeding promotion	40.2	59.0	95.0	28.4
Honduras	KMC - Kangaroo mother care	5.5	8.1	95.0	3.9
Honduras	Oral antibiotics for neonatal sepsis	0.0	0.0	0.0	0.0
Honduras	Injectable antibiotics for neonatal sepsis	95.0	95.0	95.0	77.8
India	Folic acid supplementation/fortification	3.4	4.9	95.0	2.4
India	Safe abortion services	46.7	68.6	95.0	33.0
India	Post abortion case management	78.8	95.0	95.0	55.7
India	Ectopic pregnancy case management	78.8	95.0	95.0	55.7
India	TT - Tetanus toxoid vaccination	95.0	95.0	95.0	84.7
India	IPTp	0.0	0.0	95.0	0.0
India	Syphilis detection and treatment	26.2	38.5	95.0	18.5
India	Iron supplementation in pregnancy	51.6	75.8	95.0	36.5
India	Hypertensive disorder case management	16.2	23.8	95.0	11.5
India	Diabetes case management	12.6	18.6	95.0	8.9
India	Malaria case management	52.3	76.8	95.0	37.0
India	MgSO4 management of pre-eclampsia	32.1	47.1	95.0	22.7
India	Clean birth environment	86.1	95.0	95.0	60.9
India	Immediate drying and additional stimulation	95.0	95.0	95.0	68.0
India	Thermal protection	95.0	95.0	95.0	73.4
India	Clean cord care	95.0	95.0	95.0	70.9
India	Manual removal of placenta	39.3	57.6	95.0	27.8
India	Parenteral administration of anti-convulsants	75.1	95.0	95.0	53.1
India	Antibiotics for preterm or prolonged PROM	78.7	95.0	95.0	55.6
India	Parenteral administration of antibiotics	78.7	95.0	95.0	55.6
India	Assisted vaginal delivery	26.6	39.1	95.0	18.8
India	Neonatal resuscitation	57.8	84.8	95.0	40.8
India	Parenteral administration of uterotonics	93.8	95.0	95.0	66.4

Country	Intervention	10% Increase	25% increase	95% target	2% Attrition
India	Removal of retained products of conception	34.9	51.2	95.0	24.7
India	Induction of labour for post-term pregnancies	1.9	2.7	95.0	1.3
India	Antenatal corticosteroids for preterm labour	75.1	95.0	95.0	53.1
India	Breastfeeding promotion	71.1	95.0	95.0	50.3
India	KMC - Kangaroo mother care	5.3	7.7	95.0	3.7
India	Oral antibiotics for neonatal sepsis	0.0	0.0	0.0	0.0
India	Injectable antibiotics for neonatal sepsis	95.0	95.0	95.0	74.3
Indonesia	Folic acid supplementation/fortification	5.8	8.5	95.0	4.1
Indonesia	Safe abortion services	51.9	76.2	95.0	36.7
Indonesia	Post abortion case management	63.5	93.2	95.0	44.9
Indonesia	Ectopic pregnancy case management	63.5	93.2	95.0	44.9
Indonesia	TT - Tetanus toxoid vaccination	95.0	95.0	95.0	84.7
Indonesia	IPTp	0.0	0.0	95.0	0.0
Indonesia	Syphilis detection and treatment	31.5	46.3	95.0	22.3
Indonesia	Iron supplementation in pregnancy	43.8	64.3	95.0	31.0
Indonesia	Hypertensive disorder case management	28.0	41.0	95.0	19.8
Indonesia	Diabetes case management	21.8	32.0	95.0	15.4
Indonesia	Malaria case management	90.1	95.0	95.0	63.7
Indonesia	MgSO4 management of pre-eclampsia	55.2	81.1	95.0	39.1
Indonesia	Clean birth environment	69.5	95.0	95.0	49.1
Indonesia	Immediate drying and additional stimulation	77.5	95.0	95.0	54.8
Indonesia	Thermal protection	83.7	95.0	95.0	59.2
Indonesia	Clean cord care	80.8	95.0	95.0	57.1
Indonesia	Manual removal of placenta	31.7	46.5	95.0	22.4
Indonesia	Parenteral administration of anti-convulsants	60.6	88.9	95.0	42.8
Indonesia	Antibiotics for preterm or prolonged PROM	63.4	93.0	95.0	44.8
Indonesia	Parenteral administration of antibiotics	63.4	93.0	95.0	44.8
Indonesia	Assisted vaginal delivery	21.4	31.4	95.0	15.2
Indonesia	Neonatal resuscitation	46.6	68.4	95.0	32.9
Indonesia	Parenteral administration of uterotonics	75.6	95.0	95.0	53.5
Indonesia	Removal of retained products of conception	28.1	41.2	95.0	19.9
Indonesia	Induction of labour for post-term pregnancies	1.5	2.1	95.0	1.0
Indonesia	Antenatal corticosteroids for preterm labour	60.6	88.9	95.0	42.8
Indonesia	Breastfeeding promotion	53.4	78.3	95.0	37.7
Indonesia	KMC - Kangaroo mother care	4.2	6.2	95.0	3.0
Indonesia	Oral antibiotics for neonatal sepsis	0.0	0.0	0.0	0.0
Indonesia	Injectable antibiotics for neonatal sepsis	84.7	95.0	95.0	59.9
Iraq	Folic acid supplementation/fortification	4.5	6.6	95.0	3.2
Iraq	Safe abortion services	53.2	78.1	95.0	37.6
Iraq	Post abortion case management	86.4	95.0	95.0	61.1
Iraq	Ectopic pregnancy case management	86.4	95.0	95.0	61.1
Iraq	TT - Tetanus toxoid vaccination	95.0	95.0	95.0	70.6
Iraq	IPTp	0.0	0.0	95.0	0.0
Iraq	Syphilis detection and treatment	28.9	42.4	95.0	20.4
Iraq	Iron supplementation in pregnancy	0.0	0.0	95.0	0.0
Iraq	Hypertensive disorder case management	21.7	31.8	95.0	15.3
Iraq	Diabetes case management	16.9	24.8	95.0	12.0
Iraq	Malaria case management	70.0	95.0	95.0	49.5
Iraq	MgSO4 management of pre-eclampsia	43.0	63.1	95.0	30.4
Iraq	Clean birth environment	94.5	95.0	95.0	66.8
Iraq	Immediate drying and additional stimulation	95.0	95.0	95.0	74.6
Iraq	Thermal protection	95.0	95.0	95.0	80.6
Iraq	Clean cord care	95.0	95.0	95.0	77.7
Iraq	Manual removal of placenta	43.1	63.3	95.0	30.5
Iraq	Parenteral administration of anti-convulsants	82.4	95.0	95.0	58.3
Iraq	Antibiotics for preterm or prolonged PROM	86.2	95.0	95.0	61.0
Iraq	Parenteral administration of antibiotics	86.2	95.0	95.0	61.0
Iraq	Assisted vaginal delivery	29.1	42.8	95.0	20.6
Iraq	Neonatal resuscitation	63.4	93.0	95.0	44.8
Iraq	Parenteral administration of uterotonics	95.0	95.0	95.0	72.8
Iraq	Removal of retained products of conception	38.2	56.1	95.0	27.0
Iraq	Induction of labour for post-term pregnancies	2.0	2.9	95.0	1.4
Iraq	Antenatal corticosteroids for preterm labour	82.4	95.0	95.0	58.3
Iraq	Breastfeeding promotion	21.7	31.8	95.0	15.3
Iraq	KMC - Kangaroo mother care	5.8	8.5	95.0	4.1

Country	Intervention	10% Increase	25% increase	95% target	2% Attrition
Iraq	Oral antibiotics for neonatal sepsis	0.0	0.0	0.0	0.0
Iraq	Injectable antibiotics for neonatal sepsis	95.0	95.0	95.0	81.5
Jamaica	Folic acid supplementation/fortification	5.7	8.4	95.0	4.0
Jamaica	Safe abortion services	71.5	95.0	95.0	50.5
Jamaica	Post abortion case management	95.0	95.0	95.0	69.6
Jamaica	Ectopic pregnancy case management	95.0	95.0	95.0	69.6
Jamaica	TT - Tetanus toxoid vaccination	95.0	95.0	95.0	84.7
Jamaica	IPTp	0.0	0.0	95.0	0.0
Jamaica	Syphilis detection and treatment	32.1	47.1	95.0	22.7
Jamaica	Iron supplementation in pregnancy	0.0	0.0	95.0	0.0
Jamaica	Hypertensive disorder case management	27.4	40.2	95.0	19.4
Jamaica	Diabetes case management	21.3	31.3	95.0	15.1
Jamaica	Malaria case management	88.2	95.0	95.0	62.4
Jamaica	MgSO4 management of pre-eclampsia	54.2	79.5	95.0	38.3
Jamaica	Clean birth environment	95.0	95.0	95.0	76.0
Jamaica	Immediate drying and additional stimulation	95.0	95.0	95.0	84.9
Jamaica	Thermal protection	97.4	97.4	97.4	91.7
Jamaica	Clean cord care	95.0	95.0	95.0	88.5
Jamaica	Manual removal of placenta	49.1	72.1	95.0	34.7
Jamaica	Parenteral administration of anti-convulsants	93.8	95.0	95.0	66.4
Jamaica	Antibiotics for preterm or prolonged PROM	95.0	95.0	95.0	69.5
Jamaica	Parenteral administration of antibiotics	95.0	95.0	95.0	69.5
Jamaica	Assisted vaginal delivery	33.1	48.6	95.0	23.4
Jamaica	Neonatal resuscitation	72.1	95.0	95.0	51.0
Jamaica	Parenteral administration of uterotonics	95.0	95.0	95.0	82.9
Jamaica	Removal of retained products of conception	43.5	63.9	95.0	30.8
Jamaica	Induction of labour for post-term pregnancies	2.3	3.3	95.0	1.6
Jamaica	Antenatal corticosteroids for preterm labour	93.8	95.0	95.0	66.4
Jamaica	Breastfeeding promotion	23.8	35.0	95.0	16.8
Jamaica	KMC - Kangaroo mother care	6.6	9.6	95.0	4.6
Jamaica	Oral antibiotics for neonatal sepsis	0.0	0.0	0.0	0.0
Jamaica	Injectable antibiotics for neonatal sepsis	98.6	98.6	98.6	92.8
Kenya	Folic acid supplementation/fortification	3.7	5.4	95.0	2.6
Kenya	Safe abortion services	4.4	6.4	95.0	3.1
Kenya	Post abortion case management	61.4	90.1	95.0	43.4
Kenya	Ectopic pregnancy case management	61.4	90.1	95.0	43.4
Kenya	TT - Tetanus toxoid vaccination	95.0	95.0	95.0	82.8
Kenya	IPTp	46.2	67.8	95.0	32.7
Kenya	Syphilis detection and treatment	70.4	95.0	95.0	49.8
Kenya	Iron supplementation in pregnancy	10.0	14.6	95.0	7.1
Kenya	Hypertensive disorder case management	18.6	27.3	95.0	13.2
Kenya	Diabetes case management	16.6	24.4	95.0	11.8
Kenya	Malaria case management	43.5	63.9	95.0	30.8
Kenya	MgSO4 management of pre-eclampsia	32.7	48.0	95.0	23.2
Kenya	Clean birth environment	67.1	95.0	95.0	47.4
Kenya	Immediate drying and additional stimulation	71.3	95.0	95.0	50.4
Kenya	Thermal protection	80.8	95.0	95.0	57.1
Kenya	Clean cord care	78.1	95.0	95.0	55.2
Kenya	Manual removal of placenta	57.0	83.6	95.0	40.3
Kenya	Parenteral administration of anti-convulsants	79.9	95.0	95.0	56.5
Kenya	Antibiotics for preterm or prolonged PROM	62.6	91.8	95.0	44.2
Kenya	Parenteral administration of antibiotics	62.6	91.8	95.0	44.2
Kenya	Assisted vaginal delivery	6.9	10.2	95.0	4.9
Kenya	Neonatal resuscitation	67.7	95.0	95.0	47.9
Kenya	Parenteral administration of uterotonics	62.0	91.0	95.0	43.9
Kenya	Removal of retained products of conception	41.7	61.1	95.0	29.5
Kenya	Induction of labour for post-term pregnancies	1.5	2.1	95.0	1.0
Kenya	Antenatal corticosteroids for preterm labour	79.9	95.0	95.0	56.5
Kenya	Breastfeeding promotion	78.1	95.0	95.0	55.2
Kenya	KMC - Kangaroo mother care	4.1	6.0	95.0	2.9
Kenya	Oral antibiotics for neonatal sepsis	0.0	0.0	0.0	0.0
Kenya	Injectable antibiotics for neonatal sepsis	81.9	95.0	95.0	57.9
Kyrgyzstan	Folic acid supplementation/fortification	6.3	9.2	95.0	4.4
Kyrgyzstan	Safe abortion services	46.7	68.6	95.0	33.0
Kyrgyzstan	Post abortion case management	95.0	95.0	95.0	70.3

Country	Intervention	10% Increase	25% increase	95% target	2% Attrition
Kyrgyzstan	Ectopic pregnancy case management	95.0	95.0	95.0	70.3
Kyrgyzstan	TT - Tetanus toxoid vaccination	0.0	0.0	95.0	0.0
Kyrgyzstan	IPTp	0.0	0.0	95.0	0.0
Kyrgyzstan	Syphilis detection and treatment	32.9	48.2	95.0	23.2
Kyrgyzstan	Iron supplementation in pregnancy	3.1	4.5	95.0	2.2
Kyrgyzstan	Hypertensive disorder case management	30.2	44.3	95.0	21.4
Kyrgyzstan	Diabetes case management	23.6	34.6	95.0	16.7
Kyrgyzstan	Malaria case management	95.0	95.0	95.0	68.8
Kyrgyzstan	MgSO4 management of pre-eclampsia	59.6	87.5	95.0	42.2
Kyrgyzstan	Clean birth environment	95.0	95.0	95.0	76.9
Kyrgyzstan	Immediate drying and additional stimulation	95.0	95.0	95.0	85.8
Kyrgyzstan	Thermal protection	98.4	98.4	98.4	92.6
Kyrgyzstan	Clean cord care	95.0	95.0	95.0	89.4
Kyrgyzstan	Manual removal of placenta	49.6	72.9	95.0	35.1
Kyrgyzstan	Parenteral administration of anti-convulsants	94.8	95.0	95.0	67.0
Kyrgyzstan	Antibiotics for preterm or prolonged PROM	95.0	95.0	95.0	70.1
Kyrgyzstan	Parenteral administration of antibiotics	95.0	95.0	95.0	70.1
Kyrgyzstan	Assisted vaginal delivery	33.5	49.2	95.0	23.7
Kyrgyzstan	Neonatal resuscitation	72.9	95.0	95.0	51.6
Kyrgyzstan	Parenteral administration of uterotonics	95.0	95.0	95.0	83.8
Kyrgyzstan	Removal of retained products of conception	43.9	64.5	95.0	31.1
Kyrgyzstan	Induction of labour for post-term pregnancies	2.4	3.5	95.0	1.7
Kyrgyzstan	Antenatal corticosteroids for preterm labour	94.8	95.0	95.0	67.0
Kyrgyzstan	Breastfeeding promotion	52.2	76.6	95.0	36.9
Kyrgyzstan	KMC - Kangaroo mother care	6.6	9.7	95.0	4.7
Kyrgyzstan	Oral antibiotics for neonatal sepsis	0.0	0.0	0.0	0.0
Kyrgyzstan	Injectable antibiotics for neonatal sepsis	99.6	99.6	99.6	93.7
Lao PDR	Folic acid supplementation/fortification	4.1	6.1	95.0	2.9
Lao PDR	Safe abortion services	51.9	76.2	95.0	36.7
Lao PDR	Post abortion case management	64.3	94.3	95.0	45.5
Lao PDR	Ectopic pregnancy case management	64.3	94.3	95.0	45.5
Lao PDR	TT - Tetanus toxoid vaccination	95.0	95.0	95.0	84.7
Lao PDR	IPTp	0.0	0.0	95.0	0.0
Lao PDR	Syphilis detection and treatment	26.8	39.3	95.0	18.9
Lao PDR	Iron supplementation in pregnancy	33.5	49.2	95.0	23.7
Lao PDR	Hypertensive disorder case management	19.8	29.1	95.0	14.0
Lao PDR	Diabetes case management	15.6	22.9	95.0	11.0
Lao PDR	Malaria case management	64.2	94.1	95.0	45.4
Lao PDR	MgSO4 management of pre-eclampsia	39.4	57.8	95.0	27.9
Lao PDR	Clean birth environment	70.3	95.0	95.0	49.7
Lao PDR	Immediate drying and additional stimulation	78.5	95.0	95.0	55.5
Lao PDR	Thermal protection	84.8	95.0	95.0	60.0
Lao PDR	Clean cord care	81.9	95.0	95.0	57.9
Lao PDR	Manual removal of placenta	32.1	47.1	95.0	22.7
Lao PDR	Parenteral administration of anti-convulsants	61.2	89.8	95.0	43.3
Lao PDR	Antibiotics for preterm or prolonged PROM	64.2	94.1	95.0	45.4
Lao PDR	Parenteral administration of antibiotics	64.2	94.1	95.0	45.4
Lao PDR	Assisted vaginal delivery	21.7	31.8	95.0	15.3
Lao PDR	Neonatal resuscitation	47.1	69.1	95.0	33.3
Lao PDR	Parenteral administration of uterotonics	76.5	95.0	95.0	54.1
Lao PDR	Removal of retained products of conception	28.4	41.6	95.0	20.0
Lao PDR	Induction of labour for post-term pregnancies	1.5	2.1	95.0	1.0
Lao PDR	Antenatal corticosteroids for preterm labour	61.2	89.8	95.0	43.3
Lao PDR	Breastfeeding promotion	49.0	71.9	95.0	34.6
Lao PDR	KMC - Kangaroo mother care	4.3	6.3	95.0	3.0
Lao PDR	Oral antibiotics for neonatal sepsis	0.0	0.0	0.0	0.0
Lao PDR	Injectable antibiotics for neonatal sepsis	85.7	95.0	95.0	60.6
Lesotho	Folic acid supplementation/fortification	4.9	7.2	95.0	3.4
Lesotho	Safe abortion services	56.6	83.0	95.0	40.0
Lesotho	Post abortion case management	76.4	95.0	95.0	54.0
Lesotho	Ectopic pregnancy case management	76.4	95.0	95.0	54.0
Lesotho	TT - Tetanus toxoid vaccination	95.0	95.0	95.0	80.0
Lesotho	IPTp	0.0	0.0	95.0	0.0
Lesotho	Syphilis detection and treatment	31.3	45.9	95.0	22.1
Lesotho	Iron supplementation in pregnancy	68.4	95.0	95.0	48.4

Country	Intervention	10% Increase	25% increase	95% target	2% Attrition
Lesotho	Hypertensive disorder case management	23.4	34.4	95.0	16.6
Lesotho	Diabetes case management	18.2	26.8	95.0	12.9
Lesotho	Malaria case management	75.6	95.0	95.0	53.5
Lesotho	MgSO4 management of pre-eclampsia	46.3	68.0	95.0	32.8
Lesotho	Clean birth environment	83.5	95.0	95.0	59.0
Lesotho	Immediate drying and additional stimulation	93.2	95.0	95.0	65.9
Lesotho	Thermal protection	95.0	95.0	95.0	71.2
Lesotho	Clean cord care	95.0	95.0	95.0	68.7
Lesotho	Manual removal of placenta	38.1	55.9	95.0	26.9
Lesotho	Parenteral administration of anti-convulsants	72.8	95.0	95.0	51.5
Lesotho	Antibiotics for preterm or prolonged PROM	76.1	95.0	95.0	53.8
Lesotho	Parenteral administration of antibiotics	76.1	95.0	95.0	53.8
Lesotho	Assisted vaginal delivery	25.7	37.7	95.0	18.2
Lesotho	Neonatal resuscitation	56.0	82.2	95.0	39.6
Lesotho	Parenteral administration of uterotonics	91.0	95.0	95.0	64.4
Lesotho	Removal of retained products of conception	33.8	49.6	95.0	23.9
Lesotho	Induction of labour for post-term pregnancies	1.7	2.5	95.0	1.2
Lesotho	Antenatal corticosteroids for preterm labour	72.8	95.0	95.0	51.5
Lesotho	Breastfeeding promotion	87.0	95.0	95.0	61.6
Lesotho	KMC - Kangaroo mother care	5.1	7.5	95.0	3.6
Lesotho	Oral antibiotics for neonatal sepsis	0.0	0.0	0.0	0.0
Lesotho	Injectable antibiotics for neonatal sepsis	95.0	95.0	95.0	72.0
Liberia	Folic acid supplementation/fortification	5.2	7.6	95.0	3.7
Liberia	Safe abortion services	0.0	0.0	95.0	0.0
Liberia	Post abortion case management	55.7	81.7	95.0	39.4
Liberia	Ectopic pregnancy case management	55.7	81.7	95.0	39.4
Liberia	TT - Tetanus toxoid vaccination	95.0	95.0	95.0	83.8
Liberia	IPTp	72.5	95.0	95.0	51.3
Liberia	Syphilis detection and treatment	31.5	46.3	95.0	22.3
Liberia	Iron supplementation in pregnancy	28.2	41.4	95.0	20.0
Liberia	Hypertensive disorder case management	24.8	36.3	95.0	17.5
Liberia	Diabetes case management	19.3	28.3	95.0	13.6
Liberia	Malaria case management	80.0	95.0	95.0	56.6
Liberia	MgSO4 management of pre-eclampsia	49.1	72.1	95.0	34.7
Liberia	Clean birth environment	61.0	89.5	95.0	43.1
Liberia	Immediate drying and additional stimulation	68.0	95.0	95.0	48.1
Liberia	Thermal protection	73.5	95.0	95.0	52.0
Liberia	Clean cord care	70.9	95.0	95.0	50.2
Liberia	Manual removal of placenta	27.8	40.8	95.0	19.7
Liberia	Parenteral administration of anti-convulsants	53.1	77.9	95.0	37.6
Liberia	Antibiotics for preterm or prolonged PROM	55.6	81.6	95.0	39.3
Liberia	Parenteral administration of antibiotics	55.6	81.6	95.0	39.3
Liberia	Assisted vaginal delivery	18.8	27.5	95.0	13.3
Liberia	Neonatal resuscitation	40.9	60.0	95.0	28.9
Liberia	Parenteral administration of uterotonics	66.4	95.0	95.0	47.0
Liberia	Removal of retained products of conception	24.6	36.1	95.0	17.4
Liberia	Induction of labour for post-term pregnancies	1.3	2.0	95.0	0.9
Liberia	Antenatal corticosteroids for preterm labour	53.1	77.9	95.0	37.6
Liberia	Breastfeeding promotion	70.1	95.0	95.0	49.6
Liberia	KMC - Kangaroo mother care	3.7	5.4	95.0	2.6
Liberia	Oral antibiotics for neonatal sepsis	0.0	0.0	0.0	0.0
Liberia	Injectable antibiotics for neonatal sepsis	74.3	95.0	95.0	52.5
Madagascar	Folic acid supplementation/fortification	3.1	4.6	95.0	2.2
Madagascar	Safe abortion services	4.4	6.4	95.0	3.1
Madagascar	Post abortion case management	25.2	37.0	95.0	17.8
Madagascar	Ectopic pregnancy case management	25.2	37.0	95.0	17.8
Madagascar	TT - Tetanus toxoid vaccination	95.0	95.0	95.0	73.4
Madagascar	IPTp	29.7	43.6	95.0	21.0
Madagascar	Syphilis detection and treatment	16.8	24.6	95.0	11.9
Madagascar	Iron supplementation in pregnancy	10.1	14.8	95.0	7.2
Madagascar	Hypertensive disorder case management	15.0	22.1	95.0	10.6
Madagascar	Diabetes case management	11.7	17.2	95.0	8.3
Madagascar	Malaria case management	48.7	71.5	95.0	34.4
Madagascar	MgSO4 management of pre-eclampsia	29.8	43.8	95.0	21.1
Madagascar	Clean birth environment	41.4	60.7	95.0	29.3

Country	Intervention	10% Increase	25% increase	95% target	2% Attrition
Madagascar	Immediate drying and additional stimulation	46.2	67.8	95.0	32.7
Madagascar	Thermal protection	49.9	73.2	95.0	35.3
Madagascar	Clean cord care	48.2	70.7	95.0	34.1
Madagascar	Manual removal of placenta	18.9	27.7	95.0	13.4
Madagascar	Parenteral administration of anti-convulsants	36.1	52.9	95.0	25.5
Madagascar	Antibiotics for preterm or prolonged PROM	37.8	55.5	95.0	26.7
Madagascar	Parenteral administration of antibiotics	37.8	55.5	95.0	26.7
Madagascar	Assisted vaginal delivery	12.8	18.8	95.0	9.0
Madagascar	Neonatal resuscitation	27.7	40.6	95.0	19.6
Madagascar	Parenteral administration of uterotonics	45.1	66.2	95.0	31.9
Madagascar	Removal of retained products of conception	16.8	24.6	95.0	11.9
Madagascar	Induction of labour for post-term pregnancies	0.9	1.4	95.0	0.7
Madagascar	Antenatal corticosteroids for preterm labour	36.1	52.9	95.0	25.5
Madagascar	Breastfeeding promotion	66.2	95.0	95.0	46.8
Madagascar	KMC - Kangaroo mother care	2.5	3.7	95.0	1.8
Madagascar	Oral antibiotics for neonatal sepsis	0.0	0.0	0.0	0.0
Madagascar	Injectable antibiotics for neonatal sepsis	50.4	74.0	95.0	35.7
Malawi	Folic acid supplementation/fortification	3.3	4.8	95.0	2.3
Malawi	Safe abortion services	4.4	6.4	95.0	3.1
Malawi	Post abortion case management	90.5	95.0	95.0	64.0
Malawi	Ectopic pregnancy case management	90.5	95.0	95.0	64.0
Malawi	TT - Tetanus toxoid vaccination	95.0	95.0	95.0	83.8
Malawi	IPTp	95.0	95.0	95.0	71.6
Malawi	Syphilis detection and treatment	31.3	45.9	95.0	22.1
Malawi	Iron supplementation in pregnancy	44.5	65.2	95.0	31.4
Malawi	Hypertensive disorder case management	6.7	9.8	95.0	4.7
Malawi	Diabetes case management	8.5	12.5	95.0	6.0
Malawi	Malaria case management	54.6	80.1	95.0	38.6
Malawi	MgSO4 management of pre-eclampsia	54.0	79.3	95.0	38.2
Malawi	Clean birth environment	86.0	95.0	95.0	60.8
Malawi	Immediate drying and additional stimulation	95.0	95.0	95.0	78.1
Malawi	Thermal protection	95.0	95.0	95.0	85.2
Malawi	Clean cord care	95.0	95.0	95.0	79.2
Malawi	Manual removal of placenta	6.0	8.8	95.0	4.2
Malawi	Parenteral administration of anti-convulsants	95.0	95.0	95.0	82.8
Malawi	Antibiotics for preterm or prolonged PROM	95.0	95.0	95.0	75.6
Malawi	Parenteral administration of antibiotics	95.0	95.0	95.0	75.6
Malawi	Assisted vaginal delivery	61.4	90.0	95.0	43.4
Malawi	Neonatal resuscitation	95.0	95.0	95.0	78.8
Malawi	Parenteral administration of uterotonics	95.0	95.0	95.0	84.2
Malawi	Removal of retained products of conception	39.9	58.6	95.0	28.2
Malawi	Induction of labour for post-term pregnancies	8.1	11.9	95.0	5.7
Malawi	Antenatal corticosteroids for preterm labour	95.0	95.0	95.0	82.8
Malawi	Breastfeeding promotion	75.7	95.0	95.0	53.6
Malawi	KMC - Kangaroo mother care	6.0	8.9	95.0	4.3
Malawi	Oral antibiotics for neonatal sepsis	0.0	0.0	0.0	0.0
Malawi	Injectable antibiotics for neonatal sepsis	95.0	95.0	95.0	85.4
Mali	Folic acid supplementation/fortification	2.5	3.7	95.0	1.8
Mali	Safe abortion services	0.0	0.0	95.0	0.0
Mali	Post abortion case management	29.1	42.7	95.0	20.6
Mali	Ectopic pregnancy case management	29.1	42.7	95.0	20.6
Mali	TT - Tetanus toxoid vaccination	95.0	95.0	95.0	80.0
Mali	IPTp	41.4	60.7	95.0	29.3
Mali	Syphilis detection and treatment	13.8	20.3	95.0	9.8
Mali	Iron supplementation in pregnancy	24.4	35.7	95.0	17.2
Mali	Hypertensive disorder case management	12.1	17.8	95.0	8.6
Mali	Diabetes case management	9.5	13.9	95.0	6.7
Mali	Malaria case management	39.3	57.6	95.0	27.8
Mali	MgSO4 management of pre-eclampsia	24.1	35.4	95.0	17.0
Mali	Clean birth environment	47.6	69.9	95.0	33.7
Mali	Immediate drying and additional stimulation	53.2	78.1	95.0	37.6
Mali	Thermal protection	57.4	84.2	95.0	40.6
Mali	Clean cord care	55.5	81.4	95.0	39.2
Mali	Manual removal of placenta	21.7	31.8	95.0	15.3
Mali	Parenteral administration of anti-convulsants	41.5	60.9	95.0	29.4

Country	Intervention	10% Increase	25% increase	95% target	2% Attrition
Mali	Antibiotics for preterm or prolonged PROM	43.5	63.9	95.0	30.8
Mali	Parenteral administration of antibiotics	43.5	63.9	95.0	30.8
Mali	Assisted vaginal delivery	14.6	21.5	95.0	10.4
Mali	Neonatal resuscitation	31.9	46.9	95.0	22.6
Mali	Parenteral administration of uterotonics	51.9	76.2	95.0	36.7
Mali	Removal of retained products of conception	19.3	28.3	95.0	13.6
Mali	Induction of labour for post-term pregnancies	1.1	1.6	95.0	0.8
Mali	Antenatal corticosteroids for preterm labour	41.5	60.9	95.0	29.4
Mali	Breastfeeding promotion	41.0	60.2	95.0	29.0
Mali	KMC - Kangaroo mother care	2.9	4.3	95.0	2.1
Mali	Oral antibiotics for neonatal sepsis	0.0	0.0	0.0	0.0
Mali	Injectable antibiotics for neonatal sepsis	58.2	85.4	95.0	41.1
Mauritania	Folic acid supplementation/fortification	4.2	6.1	95.0	3.0
Mauritania	Safe abortion services	0.0	0.0	95.0	0.0
Mauritania	Post abortion case management	69.2	95.0	95.0	48.9
Mauritania	Ectopic pregnancy case management	69.2	95.0	95.0	48.9
Mauritania	TT - Tetanus toxoid vaccination	95.0	95.0	95.0	75.3
Mauritania	IPTp	33.5	49.2	95.0	23.7
Mauritania	Syphilis detection and treatment	28.6	42.0	95.0	20.2
Mauritania	Iron supplementation in pregnancy	8.1	11.9	95.0	5.7
Mauritania	Hypertensive disorder case management	20.1	29.5	95.0	14.2
Mauritania	Diabetes case management	15.7	23.0	95.0	11.1
Mauritania	Malaria case management	65.0	95.0	95.0	45.9
Mauritania	MgSO4 management of pre-eclampsia	39.8	58.4	95.0	28.1
Mauritania	Clean birth environment	58.6	85.9	95.0	41.4
Mauritania	Immediate drying and additional stimulation	84.8	95.0	95.0	60.0
Mauritania	Thermal protection	91.2	95.0	95.0	64.5
Mauritania	Clean cord care	73.5	95.0	95.0	52.0
Mauritania	Manual removal of placenta	61.5	90.2	95.0	43.5
Mauritania	Parenteral administration of anti-convulsants	33.9	49.8	95.0	24.0
Mauritania	Antibiotics for preterm or prolonged PROM	63.2	92.8	95.0	44.7
Mauritania	Parenteral administration of antibiotics	63.2	92.8	95.0	44.7
Mauritania	Assisted vaginal delivery	31.0	45.5	95.0	21.9
Mauritania	Neonatal resuscitation	43.1	63.3	95.0	30.5
Mauritania	Parenteral administration of uterotonics	72.9	95.0	95.0	51.6
Mauritania	Removal of retained products of conception	37.4	54.9	95.0	26.4
Mauritania	Induction of labour for post-term pregnancies	8.9	13.1	95.0	6.3
Mauritania	Antenatal corticosteroids for preterm labour	33.9	49.8	95.0	24.0
Mauritania	Breastfeeding promotion	52.4	77.0	95.0	37.1
Mauritania	KMC - Kangaroo mother care	4.6	6.8	95.0	3.3
Mauritania	Oral antibiotics for neonatal sepsis	0.0	0.0	0.0	0.0
Mauritania	Injectable antibiotics for neonatal sepsis	92.2	95.0	95.0	65.2
Mexico	Folic acid supplementation/fortification	6.3	9.2	95.0	4.4
Mexico	Safe abortion services	0.8	1.2	95.0	0.6
Mexico	Post abortion case management	95.0	95.0	95.0	68.4
Mexico	Ectopic pregnancy case management	95.0	95.0	95.0	68.4
Mexico	TT - Tetanus toxoid vaccination	96.0	96.0	96.0	90.4
Mexico	IPTp	0.0	0.0	95.0	0.0
Mexico	Syphilis detection and treatment	32.3	47.5	95.0	22.9
Mexico	Iron supplementation in pregnancy	0.0	0.0	95.0	0.0
Mexico	Hypertensive disorder case management	30.1	44.1	95.0	21.3
Mexico	Diabetes case management	23.4	34.4	95.0	16.6
Mexico	Malaria case management	95.0	95.0	95.0	68.6
Mexico	MgSO4 management of pre-eclampsia	59.5	87.3	95.0	42.1
Mexico	Clean birth environment	95.0	95.0	95.0	74.7
Mexico	Immediate drying and additional stimulation	95.0	95.0	95.0	83.5
Mexico	Thermal protection	95.7	95.7	95.7	90.1
Mexico	Clean cord care	95.0	95.0	95.0	87.0
Mexico	Manual removal of placenta	48.2	70.7	95.0	34.1
Mexico	Parenteral administration of anti-convulsants	92.2	95.0	95.0	65.2
Mexico	Antibiotics for preterm or prolonged PROM	95.0	95.0	95.0	68.2
Mexico	Parenteral administration of antibiotics	95.0	95.0	95.0	68.2
Mexico	Assisted vaginal delivery	32.6	47.9	95.0	23.1
Mexico	Neonatal resuscitation	70.9	95.0	95.0	50.2
Mexico	Parenteral administration of uterotonics	95.0	95.0	95.0	81.5

Country	Intervention	10% Increase	25% increase	95% target	2% Attrition
Mexico	Removal of retained products of conception	42.7	62.7	95.0	30.2
Mexico	Induction of labour for post-term pregnancies	2.3	3.3	95.0	1.6
Mexico	Antenatal corticosteroids for preterm labour	92.2	95.0	95.0	65.2
Mexico	Breastfeeding promotion	41.7	61.1	95.0	29.5
Mexico	KMC - Kangaroo mother care	6.4	9.5	95.0	4.6
Mexico	Oral antibiotics for neonatal sepsis	0.0	0.0	0.0	0.0
Mexico	Injectable antibiotics for neonatal sepsis	96.9	96.9	96.9	91.2
Morocco	Folic acid supplementation/fortification	2.0	2.9	95.0	1.4
Morocco	Safe abortion services	2.9	4.3	95.0	2.1
Morocco	Post abortion case management	72.6	95.0	95.0	51.3
Morocco	Ectopic pregnancy case management	72.6	95.0	95.0	51.3
Morocco	TT - Tetanus toxoid vaccination	95.0	95.0	95.0	82.8
Morocco	IPTp	0.0	0.0	95.0	0.0
Morocco	Syphilis detection and treatment	22.2	32.6	95.0	15.7
Morocco	Iron supplementation in pregnancy	6.9	10.2	95.0	4.9
Morocco	Hypertensive disorder case management	9.6	14.1	95.0	6.8
Morocco	Diabetes case management	7.5	10.9	95.0	5.3
Morocco	Malaria case management	31.0	45.5	95.0	21.9
Morocco	MgSO4 management of pre-eclampsia	19.0	27.9	95.0	13.5
Morocco	Clean birth environment	79.3	95.0	95.0	56.1
Morocco	Immediate drying and additional stimulation	88.6	95.0	95.0	62.7
Morocco	Thermal protection	95.0	95.0	95.0	67.7
Morocco	Clean cord care	92.4	95.0	95.0	65.3
Morocco	Manual removal of placenta	36.2	53.1	95.0	25.6
Morocco	Parenteral administration of anti-convulsants	69.2	95.0	95.0	48.9
Morocco	Antibiotics for preterm or prolonged PROM	72.4	95.0	95.0	51.2
Morocco	Parenteral administration of antibiotics	72.4	95.0	95.0	51.2
Morocco	Assisted vaginal delivery	24.5	35.9	95.0	17.3
Morocco	Neonatal resuscitation	53.2	78.1	95.0	37.6
Morocco	Parenteral administration of uterotonics	86.5	95.0	95.0	61.2
Morocco	Removal of retained products of conception	32.1	47.1	95.0	22.7
Morocco	Induction of labour for post-term pregnancies	1.7	2.5	95.0	1.2
Morocco	Antenatal corticosteroids for preterm labour	69.2	95.0	95.0	48.9
Morocco	Breastfeeding promotion	35.3	51.8	95.0	24.9
Morocco	KMC - Kangaroo mother care	4.8	7.1	95.0	3.4
Morocco	Oral antibiotics for neonatal sepsis	0.0	0.0	0.0	0.0
Morocco	Injectable antibiotics for neonatal sepsis	95.0	95.0	95.0	68.4
Mozambique	Folic acid supplementation/fortification	3.6	5.3	95.0	2.5
Mozambique	Safe abortion services	4.4	6.4	95.0	3.1
Mozambique	Post abortion case management	64.7	94.9	95.0	45.7
Mozambique	Ectopic pregnancy case management	64.7	94.9	95.0	45.7
Mozambique	TT - Tetanus toxoid vaccination	95.0	95.0	95.0	80.9
Mozambique	IPTp	47.6	69.9	95.0	33.7
Mozambique	Syphilis detection and treatment	29.8	43.8	95.0	21.1
Mozambique	Iron supplementation in pregnancy	34.5	50.6	95.0	24.4
Mozambique	Hypertensive disorder case management	17.2	25.2	95.0	12.1
Mozambique	Diabetes case management	13.4	19.7	95.0	9.5
Mozambique	Malaria case management	55.5	81.4	95.0	39.2
Mozambique	MgSO4 management of pre-eclampsia	34.1	50.0	95.0	24.1
Mozambique	Clean birth environment	70.8	95.0	95.0	50.1
Mozambique	Immediate drying and additional stimulation	79.1	95.0	95.0	55.9
Mozambique	Thermal protection	85.3	95.0	95.0	60.3
Mozambique	Clean cord care	82.4	95.0	95.0	58.3
Mozambique	Manual removal of placenta	32.3	47.5	95.0	22.9
Mozambique	Parenteral administration of anti-convulsants	61.8	90.6	95.0	43.7
Mozambique	Antibiotics for preterm or prolonged PROM	64.6	94.7	95.0	45.6
Mozambique	Parenteral administration of antibiotics	64.6	94.7	95.0	45.6
Mozambique	Assisted vaginal delivery	21.8	32.0	95.0	15.4
Mozambique	Neonatal resuscitation	47.5	69.7	95.0	33.6
Mozambique	Parenteral administration of uterotonics	77.1	95.0	95.0	54.5
Mozambique	Removal of retained products of conception	28.6	42.0	95.0	20.2
Mozambique	Induction of labour for post-term pregnancies	1.5	2.1	95.0	1.0
Mozambique	Antenatal corticosteroids for preterm labour	61.8	90.6	95.0	43.7
Mozambique	Breastfeeding promotion	69.3	95.0	95.0	49.0
Mozambique	KMC - Kangaroo mother care	4.3	6.3	95.0	3.0

Country	Intervention	10% Increase	25% increase	95% target	2% Attrition
Mozambique	Oral antibiotics for neonatal sepsis	0.0	0.0	0.0	0.0
Mozambique	Injectable antibiotics for neonatal sepsis	86.2	95.0	95.0	61.0
Myanmar	Folic acid supplementation/fortification	4.0	5.8	95.0	2.8
Myanmar	Safe abortion services	51.9	76.2	95.0	36.7
Myanmar	Post abortion case management	24.7	36.2	95.0	17.5
Myanmar	Ectopic pregnancy case management	24.7	36.2	95.0	17.5
Myanmar	TT - Tetanus toxoid vaccination	95.0	95.0	95.0	84.7
Myanmar	IPTp	0.0	0.0	95.0	0.0
Myanmar	Syphilis detection and treatment	27.3	40.0	95.0	19.3
Myanmar	Iron supplementation in pregnancy	78.9	95.0	95.0	55.8
Myanmar	Hypertensive disorder case management	19.0	27.9	95.0	13.5
Myanmar	Diabetes case management	14.9	21.9	95.0	10.5
Myanmar	Malaria case management	61.4	90.0	95.0	43.4
Myanmar	MgSO4 management of pre-eclampsia	37.7	55.3	95.0	26.6
Myanmar	Clean birth environment	40.5	59.4	95.0	28.6
Myanmar	Immediate drying and additional stimulation	45.1	66.2	95.0	31.9
Myanmar	Thermal protection	48.7	71.5	95.0	34.4
Myanmar	Clean cord care	47.1	69.1	95.0	33.3
Myanmar	Manual removal of placenta	18.5	27.1	95.0	13.1
Myanmar	Parenteral administration of anti-convulsants	35.3	51.8	95.0	24.9
Myanmar	Antibiotics for preterm or prolonged PROM	36.9	54.1	95.0	26.1
Myanmar	Parenteral administration of antibiotics	36.9	54.1	95.0	26.1
Myanmar	Assisted vaginal delivery	12.5	18.4	95.0	8.8
Myanmar	Neonatal resuscitation	27.2	39.8	95.0	19.2
Myanmar	Parenteral administration of uterotonics	44.1	64.6	95.0	31.2
Myanmar	Removal of retained products of conception	16.4	24.0	95.0	11.6
Myanmar	Induction of labour for post-term pregnancies	0.9	1.4	95.0	0.7
Myanmar	Antenatal corticosteroids for preterm labour	35.3	51.8	95.0	24.9
Myanmar	Breastfeeding promotion	65.4	95.0	95.0	46.2
Myanmar	KMC - Kangaroo mother care	2.5	3.6	95.0	1.7
Myanmar	Oral antibiotics for neonatal sepsis	0.0	0.0	0.0	0.0
Myanmar	Injectable antibiotics for neonatal sepsis	49.4	72.5	95.0	34.9
Namibia	Folic acid supplementation/fortification	4.2	6.1	95.0	2.9
Namibia	Safe abortion services	56.6	83.0	95.0	40.0
Namibia	Post abortion case management	87.5	95.0	95.0	61.9
Namibia	Ectopic pregnancy case management	87.5	95.0	95.0	61.9
Namibia	TT - Tetanus toxoid vaccination	95.0	95.0	95.0	82.8
Namibia	IPTp	6.8	10.0	95.0	4.8
Namibia	Syphilis detection and treatment	95.0	95.0	95.0	87.2
Namibia	Iron supplementation in pregnancy	51.4	75.4	95.0	36.3
Namibia	Hypertensive disorder case management	56.0	82.2	95.0	39.6
Namibia	Diabetes case management	42.9	62.9	95.0	30.3
Namibia	Malaria case management	60.6	88.9	95.0	42.8
Namibia	MgSO4 management of pre-eclampsia	42.3	62.1	95.0	29.9
Namibia	Clean birth environment	92.0	95.0	95.0	65.0
Namibia	Immediate drying and additional stimulation	95.0	95.0	95.0	81.4
Namibia	Thermal protection	95.0	95.0	95.0	81.6
Namibia	Clean cord care	95.0	95.0	95.0	82.1
Namibia	Manual removal of placenta	86.9	95.0	95.0	61.5
Namibia	Parenteral administration of anti-convulsants	95.0	95.0	95.0	80.9
Namibia	Antibiotics for preterm or prolonged PROM	95.0	95.0	95.0	77.1
Namibia	Parenteral administration of antibiotics	95.0	95.0	95.0	77.1
Namibia	Assisted vaginal delivery	52.4	77.0	95.0	37.1
Namibia	Neonatal resuscitation	95.0	95.0	95.0	73.6
Namibia	Parenteral administration of uterotonics	95.0	95.0	95.0	72.4
Namibia	Removal of retained products of conception	30.6	44.9	95.0	21.6
Namibia	Induction of labour for post-term pregnancies	2.0	2.9	95.0	1.4
Namibia	Antenatal corticosteroids for preterm labour	95.0	95.0	95.0	80.9
Namibia	Breastfeeding promotion	61.1	89.6	95.0	43.2
Namibia	KMC - Kangaroo mother care	5.8	8.6	95.0	4.1
Namibia	Oral antibiotics for neonatal sepsis	0.0	0.0	0.0	0.0
Namibia	Injectable antibiotics for neonatal sepsis	95.0	95.0	95.0	82.5
Nepal	Folic acid supplementation/fortification	4.7	6.9	95.0	3.3
Nepal	Safe abortion services	46.7	68.6	95.0	33.0
Nepal	Post abortion case management	57.3	84.1	95.0	40.5

Country	Intervention	10% Increase	25% increase	95% target	2% Attrition
Nepal	Ectopic pregnancy case management	57.3	84.1	95.0	40.5
Nepal	TT - Tetanus toxoid vaccination	95.0	95.0	95.0	83.8
Nepal	IPTp	0.0	0.0	95.0	0.0
Nepal	Syphilis detection and treatment	10.9	16.0	95.0	7.7
Nepal	Iron supplementation in pregnancy	94.4	95.0	95.0	66.7
Nepal	Hypertensive disorder case management	20.2	29.7	95.0	14.3
Nepal	Diabetes case management	11.3	16.6	95.0	8.0
Nepal	Malaria case management	29.0	42.6	95.0	20.5
Nepal	MgSO4 management of pre-eclampsia	55.4	81.3	95.0	39.2
Nepal	Clean birth environment	72.0	95.0	95.0	50.9
Nepal	Immediate drying and additional stimulation	73.9	95.0	95.0	52.2
Nepal	Thermal protection	75.5	95.0	95.0	53.4
Nepal	Clean cord care	75.3	95.0	95.0	53.3
Nepal	Manual removal of placenta	20.4	29.9	95.0	14.4
Nepal	Parenteral administration of anti-convulsants	70.3	95.0	95.0	49.7
Nepal	Antibiotics for preterm or prolonged PROM	59.0	86.5	95.0	41.7
Nepal	Parenteral administration of antibiotics	59.0	86.5	95.0	41.7
Nepal	Assisted vaginal delivery	53.1	77.9	95.0	37.6
Nepal	Neonatal resuscitation	70.9	95.0	95.0	50.2
Nepal	Parenteral administration of uterotonics	74.3	95.0	95.0	52.5
Nepal	Removal of retained products of conception	49.9	73.2	95.0	35.3
Nepal	Induction of labour for post-term pregnancies	25.2	36.9	95.0	17.8
Nepal	Antenatal corticosteroids for preterm labour	70.3	95.0	95.0	49.7
Nepal	Breastfeeding promotion	84.3	95.0	95.0	59.6
Nepal	KMC - Kangaroo mother care	3.8	5.6	95.0	2.7
Nepal	Oral antibiotics for neonatal sepsis	0.0	0.0	0.0	0.0
Nepal	Injectable antibiotics for neonatal sepsis	76.4	95.0	95.0	54.0
Nicaragua	Folic acid supplementation/fortification	5.8	8.6	95.0	4.1
Nicaragua	Safe abortion services	0.8	1.2	95.0	0.6
Nicaragua	Post abortion case management	75.1	95.0	95.0	53.1
Nicaragua	Ectopic pregnancy case management	75.1	95.0	95.0	53.1
Nicaragua	TT - Tetanus toxoid vaccination	95.0	95.0	95.0	84.7
Nicaragua	IPTp	0.0	0.0	95.0	0.0
Nicaragua	Syphilis detection and treatment	28.9	42.4	95.0	20.4
Nicaragua	Iron supplementation in pregnancy	82.0	95.0	95.0	58.0
Nicaragua	Hypertensive disorder case management	28.1	41.2	95.0	19.9
Nicaragua	Diabetes case management	21.8	32.0	95.0	15.4
Nicaragua	Malaria case management	90.6	95.0	95.0	64.1
Nicaragua	MgSO4 management of pre-eclampsia	55.5	81.4	95.0	39.2
Nicaragua	Clean birth environment	82.1	95.0	95.0	58.1
Nicaragua	Immediate drying and additional stimulation	91.7	95.0	95.0	64.8
Nicaragua	Thermal protection	95.0	95.0	95.0	70.0
Nicaragua	Clean cord care	95.0	95.0	95.0	67.6
Nicaragua	Manual removal of placenta	37.4	54.9	95.0	26.4
Nicaragua	Parenteral administration of anti-convulsants	71.6	95.0	95.0	50.6
Nicaragua	Antibiotics for preterm or prolonged PROM	74.9	95.0	95.0	53.0
Nicaragua	Parenteral administration of antibiotics	74.9	95.0	95.0	53.0
Nicaragua	Assisted vaginal delivery	25.3	37.1	95.0	17.9
Nicaragua	Neonatal resuscitation	55.1	80.9	95.0	39.0
Nicaragua	Parenteral administration of uterotonics	89.4	95.0	95.0	63.2
Nicaragua	Removal of retained products of conception	33.1	48.6	95.0	23.4
Nicaragua	Induction of labour for post-term pregnancies	1.7	2.5	95.0	1.2
Nicaragua	Antenatal corticosteroids for preterm labour	71.6	95.0	95.0	50.6
Nicaragua	Breastfeeding promotion	36.2	53.1	95.0	25.6
Nicaragua	KMC - Kangaroo mother care	5.0	7.3	95.0	3.5
Nicaragua	Oral antibiotics for neonatal sepsis	0.0	0.0	0.0	0.0
Nicaragua	Injectable antibiotics for neonatal sepsis	95.0	95.0	95.0	70.8
Niger	Folic acid supplementation/fortification	2.2	3.2	95.0	1.6
Niger	Safe abortion services	0.0	0.0	95.0	0.0
Niger	Post abortion case management	4.0	5.8	95.0	2.8
Niger	Ectopic pregnancy case management	4.0	5.8	95.0	2.8
Niger	TT - Tetanus toxoid vaccination	95.0	95.0	95.0	76.2
Niger	IPTp	49.1	72.1	95.0	34.7
Niger	Syphilis detection and treatment	27.7	40.6	95.0	19.6
Niger	Iron supplementation in pregnancy	38.1	55.9	95.0	26.9

Country	Intervention	10% Increase	25% increase	95% target	2% Attrition
Niger	Hypertensive disorder case management	10.6	15.6	95.0	7.5
Niger	Diabetes case management	8.3	12.1	95.0	5.8
Niger	Malaria case management	34.3	50.4	95.0	24.3
Niger	MgSO4 management of pre-eclampsia	21.0	30.9	95.0	14.9
Niger	Clean birth environment	32.5	47.7	95.0	23.0
Niger	Immediate drying and additional stimulation	36.2	53.1	95.0	25.6
Niger	Thermal protection	39.1	57.4	95.0	27.7
Niger	Clean cord care	37.8	55.5	95.0	26.7
Niger	Manual removal of placenta	14.8	21.7	95.0	10.4
Niger	Parenteral administration of anti-convulsants	28.4	41.6	95.0	20.0
Niger	Antibiotics for preterm or prolonged PROM	29.7	43.6	95.0	21.0
Niger	Parenteral administration of antibiotics	29.7	43.6	95.0	21.0
Niger	Assisted vaginal delivery	10.0	14.6	95.0	7.1
Niger	Neonatal resuscitation	21.8	32.0	95.0	15.4
Niger	Parenteral administration of uterotonics	35.4	52.0	95.0	25.0
Niger	Removal of retained products of conception	13.2	19.3	95.0	9.3
Niger	Induction of labour for post-term pregnancies	0.7	1.0	95.0	0.5
Niger	Antenatal corticosteroids for preterm labour	28.4	41.6	95.0	20.0
Niger	Breastfeeding promotion	28.9	42.4	95.0	20.4
Niger	KMC - Kangaroo mother care	2.0	2.9	95.0	1.4
Niger	Oral antibiotics for neonatal sepsis	0.0	0.0	0.0	0.0
Niger	Injectable antibiotics for neonatal sepsis	39.7	58.2	95.0	28.0
Nigeria	Folic acid supplementation/fortification	3.3	4.8	95.0	2.3
Nigeria	Safe abortion services	0.0	0.0	95.0	0.0
Nigeria	Post abortion case management	25.0	36.6	95.0	17.6
Nigeria	Ectopic pregnancy case management	25.0	36.6	95.0	17.6
Nigeria	TT - Tetanus toxoid vaccination	73.2	95.0	95.0	51.8
Nigeria	IPTp	28.4	41.6	95.0	20.0
Nigeria	Syphilis detection and treatment	21.7	31.8	95.0	15.3
Nigeria	Iron supplementation in pregnancy	27.3	40.0	95.0	19.3
Nigeria	Hypertensive disorder case management	15.7	23.0	95.0	11.1
Nigeria	Diabetes case management	12.2	18.0	95.0	8.7
Nigeria	Malaria case management	50.7	74.4	95.0	35.9
Nigeria	MgSO4 management of pre-eclampsia	31.0	45.5	95.0	21.9
Nigeria	Clean birth environment	41.0	60.2	95.0	29.0
Nigeria	Immediate drying and additional stimulation	45.8	67.2	95.0	32.4
Nigeria	Thermal protection	49.4	72.5	95.0	34.9
Nigeria	Clean cord care	47.6	69.9	95.0	33.7
Nigeria	Manual removal of placenta	18.6	27.3	95.0	13.2
Nigeria	Parenteral administration of anti-convulsants	35.7	52.3	95.0	25.2
Nigeria	Antibiotics for preterm or prolonged PROM	37.4	54.9	95.0	26.4
Nigeria	Parenteral administration of antibiotics	37.4	54.9	95.0	26.4
Nigeria	Assisted vaginal delivery	12.6	18.6	95.0	8.9
Nigeria	Neonatal resuscitation	27.4	40.2	95.0	19.4
Nigeria	Parenteral administration of uterotonics	44.6	65.4	95.0	31.5
Nigeria	Removal of retained products of conception	16.5	24.2	95.0	11.7
Nigeria	Induction of labour for post-term pregnancies	0.9	1.4	95.0	0.7
Nigeria	Antenatal corticosteroids for preterm labour	35.7	52.3	95.0	25.2
Nigeria	Breastfeeding promotion	31.4	46.1	95.0	22.2
Nigeria	KMC - Kangaroo mother care	2.5	3.7	95.0	1.8
Nigeria	Oral antibiotics for neonatal sepsis	0.0	0.0	0.0	0.0
Nigeria	Injectable antibiotics for neonatal sepsis	49.9	73.2	95.0	35.3
Pakistan	Folic acid supplementation/fortification	3.4	5.0	95.0	2.4
Pakistan	Safe abortion services	46.7	68.6	95.0	33.0
Pakistan	Post abortion case management	66.1	95.0	95.0	46.7
Pakistan	Ectopic pregnancy case management	66.1	95.0	95.0	46.7
Pakistan	TT - Tetanus toxoid vaccination	95.0	95.0	95.0	80.0
Pakistan	IPTp	0.0	0.0	95.0	0.0
Pakistan	Syphilis detection and treatment	28.7	42.2	95.0	20.3
Pakistan	Iron supplementation in pregnancy	28.5	41.8	95.0	20.1
Pakistan	Hypertensive disorder case management	16.4	24.0	95.0	11.6
Pakistan	Diabetes case management	12.8	18.8	95.0	9.0
Pakistan	Malaria case management	53.0	77.7	95.0	37.5
Pakistan	MgSO4 management of pre-eclampsia	32.5	47.7	95.0	23.0
Pakistan	Clean birth environment	72.3	95.0	95.0	51.1

Country	Intervention	10% Increase	25% increase	95% target	2% Attrition
Pakistan	Immediate drying and additional stimulation	80.7	95.0	95.0	57.0
Pakistan	Thermal protection	87.0	95.0	95.0	61.6
Pakistan	Clean cord care	84.1	95.0	95.0	59.5
Pakistan	Manual removal of placenta	33.0	48.4	95.0	23.3
Pakistan	Parenteral administration of anti-convulsants	63.0	92.4	95.0	44.5
Pakistan	Antibiotics for preterm or prolonged PROM	65.9	95.0	95.0	46.6
Pakistan	Parenteral administration of antibiotics	65.9	95.0	95.0	46.6
Pakistan	Assisted vaginal delivery	22.2	32.6	95.0	15.7
Pakistan	Neonatal resuscitation	48.4	71.1	95.0	34.3
Pakistan	Parenteral administration of uterotonics	78.8	95.0	95.0	55.7
Pakistan	Removal of retained products of conception	29.1	42.8	95.0	20.6
Pakistan	Induction of labour for post-term pregnancies	1.6	2.3	95.0	1.1
Pakistan	Antenatal corticosteroids for preterm labour	63.0	92.4	95.0	44.5
Pakistan	Breastfeeding promotion	48.4	71.1	95.0	34.3
Pakistan	KMC - Kangaroo mother care	4.4	6.5	95.0	3.1
Pakistan	Oral antibiotics for neonatal sepsis	0.0	0.0	0.0	0.0
Pakistan	Injectable antibiotics for neonatal sepsis	88.1	95.0	95.0	62.3
Panama	Folic acid supplementation/fortification	5.8	8.6	95.0	4.1
Panama	Safe abortion services	0.8	1.2	95.0	0.6
Panama	Post abortion case management	91.0	95.0	95.0	64.4
Panama	Ectopic pregnancy case management	91.0	95.0	95.0	64.4
Panama	TT - Tetanus toxoid vaccination	0.0	0.0	95.0	0.0
Panama	IPTp	0.0	0.0	95.0	0.0
Panama	Syphilis detection and treatment	30.7	45.1	95.0	21.7
Panama	Iron supplementation in pregnancy	0.0	0.0	95.0	0.0
Panama	Hypertensive disorder case management	28.1	41.2	95.0	19.9
Panama	Diabetes case management	22.0	32.2	95.0	15.5
Panama	Malaria case management	90.6	95.0	95.0	64.1
Panama	MgSO4 management of pre-eclampsia	55.6	81.6	95.0	39.3
Panama	Clean birth environment	95.0	95.0	95.0	70.4
Panama	Immediate drying and additional stimulation	95.0	95.0	95.0	78.6
Panama	Thermal protection	95.0	95.0	95.0	84.9
Panama	Clean cord care	95.0	95.0	95.0	81.9
Panama	Manual removal of placenta	45.4	66.6	95.0	32.1
Panama	Parenteral administration of anti-convulsants	86.8	95.0	95.0	61.4
Panama	Antibiotics for preterm or prolonged PROM	90.9	95.0	95.0	64.3
Panama	Parenteral administration of antibiotics	90.9	95.0	95.0	64.3
Panama	Assisted vaginal delivery	30.7	45.1	95.0	21.7
Panama	Neonatal resuscitation	66.8	95.0	95.0	47.2
Panama	Parenteral administration of uterotonics	95.0	95.0	95.0	76.7
Panama	Removal of retained products of conception	40.2	59.0	95.0	28.4
Panama	Induction of labour for post-term pregnancies	2.1	3.1	95.0	1.5
Panama	Antenatal corticosteroids for preterm labour	86.8	95.0	95.0	61.4
Panama	Breastfeeding promotion	24.4	35.7	95.0	17.2
Panama	KMC - Kangaroo mother care	6.1	8.9	95.0	4.3
Panama	Oral antibiotics for neonatal sepsis	0.0	0.0	0.0	0.0
Panama	Injectable antibiotics for neonatal sepsis	95.0	95.0	95.0	85.8
Papua New Guinea	Folic acid supplementation/fortification	3.7	5.4	95.0	2.6
Papua New Guinea	Safe abortion services	95.0	95.0	95.0	80.0
Papua New Guinea	Post abortion case management	51.7	75.9	95.0	36.6
Papua New Guinea	Ectopic pregnancy case management	51.7	75.9	95.0	36.6
Papua New Guinea	TT - Tetanus toxoid vaccination	93.2	95.0	95.0	65.9
Papua New Guinea	IPTp	0.0	0.0	95.0	0.0
Papua New Guinea	Syphilis detection and treatment	24.4	35.7	95.0	17.2
Papua New Guinea	Iron supplementation in pregnancy	0.0	0.0	95.0	0.0
Papua New Guinea	Hypertensive disorder case management	17.6	25.8	95.0	12.4
Papua New Guinea	Diabetes case management	13.7	20.1	95.0	9.7
Papua New Guinea	Malaria case management	56.7	83.2	95.0	40.1
Papua New Guinea	MgSO4 management of pre-eclampsia	34.7	51.0	95.0	24.6
Papua New Guinea	Clean birth environment	56.6	83.0	95.0	40.0
Papua New Guinea	Immediate drying and additional stimulation	63.1	92.6	95.0	44.6
Papua New Guinea	Thermal protection	68.1	95.0	95.0	48.2
Papua New Guinea	Clean cord care	65.8	95.0	95.0	46.5
Papua New Guinea	Manual removal of placenta	25.8	37.9	95.0	18.3
Papua New Guinea	Parenteral administration of anti-convulsants	49.2	72.3	95.0	34.8

Country	Intervention	10% Increase	25% increase	95% target	2% Attrition
Papua New Guinea	Antibiotics for preterm or prolonged PROM	51.6	75.8	95.0	36.5
Papua New Guinea	Parenteral administration of antibiotics	51.6	75.8	95.0	36.5
Papua New Guinea	Assisted vaginal delivery	17.4	25.6	95.0	12.3
Papua New Guinea	Neonatal resuscitation	37.9	55.7	95.0	26.8
Papua New Guinea	Parenteral administration of uterotonics	61.6	90.4	95.0	43.6
Papua New Guinea	Removal of retained products of conception	22.9	33.6	95.0	16.2
Papua New Guinea	Induction of labour for post-term pregnancies	1.2	1.8	95.0	0.8
Papua New Guinea	Antenatal corticosteroids for preterm labour	49.2	72.3	95.0	34.8
Papua New Guinea	Breastfeeding promotion	0.0	0.0	95.0	0.0
Papua New Guinea	KMC - Kangaroo mother care	3.4	5.1	95.0	2.4
Papua New Guinea	Oral antibiotics for neonatal sepsis	0.0	0.0	0.0	0.0
Papua New Guinea	Injectable antibiotics for neonatal sepsis	68.9	95.0	95.0	48.8
Paraguay	Folic acid supplementation/fortification	6.2	9.1	95.0	4.4
Paraguay	Safe abortion services	0.3	0.4	95.0	0.2
Paraguay	Post abortion case management	93.0	95.0	95.0	65.8
Paraguay	Ectopic pregnancy case management	93.0	95.0	95.0	65.8
Paraguay	TT - Tetanus toxoid vaccination	95.0	95.0	95.0	89.4
Paraguay	IPTp	0.0	0.0	95.0	0.0
Paraguay	Syphilis detection and treatment	32.5	47.7	95.0	23.0
Paraguay	Iron supplementation in pregnancy	0.0	0.0	95.0	0.0
Paraguay	Hypertensive disorder case management	29.9	43.9	95.0	21.2
Paraguay	Diabetes case management	23.3	34.2	95.0	16.5
Paraguay	Malaria case management	95.0	95.0	95.0	68.2
Paraguay	MgSO4 management of pre-eclampsia	59.2	86.9	95.0	41.9
Paraguay	Clean birth environment	95.0	95.0	95.0	71.9
Paraguay	Immediate drying and additional stimulation	95.0	95.0	95.0	80.3
Paraguay	Thermal protection	95.0	95.0	95.0	86.7
Paraguay	Clean cord care	95.0	95.0	95.0	83.7
Paraguay	Manual removal of placenta	46.5	68.2	95.0	32.8
Paraguay	Parenteral administration of anti-convulsants	88.8	95.0	95.0	62.8
Paraguay	Antibiotics for preterm or prolonged PROM	92.9	95.0	95.0	65.7
Paraguay	Parenteral administration of antibiotics	92.9	95.0	95.0	65.7
Paraguay	Assisted vaginal delivery	31.4	46.1	95.0	22.2
Paraguay	Neonatal resuscitation	68.3	95.0	95.0	48.3
Paraguay	Parenteral administration of uterotonics	95.0	95.0	95.0	78.4
Paraguay	Removal of retained products of conception	41.1	60.4	95.0	29.1
Paraguay	Induction of labour for post-term pregnancies	2.1	3.1	95.0	1.5
Paraguay	Antenatal corticosteroids for preterm labour	88.8	95.0	95.0	62.8
Paraguay	Breastfeeding promotion	40.1	58.8	95.0	28.3
Paraguay	KMC - Kangaroo mother care	6.2	9.1	95.0	4.4
Paraguay	Oral antibiotics for neonatal sepsis	0.0	0.0	0.0	0.0
Paraguay	Injectable antibiotics for neonatal sepsis	95.0	95.0	95.0	87.7
Peru	Folic acid supplementation/fortification	6.4	9.3	95.0	4.5
Peru	Safe abortion services	0.3	0.4	95.0	0.2
Peru	Post abortion case management	90.5	95.0	95.0	64.0
Peru	Ectopic pregnancy case management	90.5	95.0	95.0	64.0
Peru	TT - Tetanus toxoid vaccination	95.0	95.0	95.0	89.4
Peru	IPTp	0.0	0.0	95.0	0.0
Peru	Syphilis detection and treatment	32.1	47.1	95.0	22.7
Peru	Iron supplementation in pregnancy	79.5	95.0	95.0	56.2
Peru	Hypertensive disorder case management	30.6	44.9	95.0	21.6
Peru	Diabetes case management	23.8	35.0	95.0	16.8
Peru	Malaria case management	95.0	95.0	95.0	69.8
Peru	MgSO4 management of pre-eclampsia	60.6	88.9	95.0	42.8
Peru	Clean birth environment	95.0	95.0	95.0	70.0
Peru	Immediate drying and additional stimulation	95.0	95.0	95.0	78.2
Peru	Thermal protection	95.0	95.0	95.0	84.4
Peru	Clean cord care	95.0	95.0	95.0	81.5
Peru	Manual removal of placenta	45.1	66.2	95.0	31.9
Peru	Parenteral administration of anti-convulsants	86.4	95.0	95.0	61.1
Peru	Antibiotics for preterm or prolonged PROM	90.4	95.0	95.0	63.9
Peru	Parenteral administration of antibiotics	90.4	95.0	95.0	63.9
Peru	Assisted vaginal delivery	30.5	44.7	95.0	21.6
Peru	Neonatal resuscitation	66.4	95.0	95.0	47.0
Peru	Parenteral administration of uterotonics	95.0	95.0	95.0	76.3

Country	Intervention	10% Increase	25% increase	95% target	2% Attrition
Peru	Removal of retained products of conception	40.1	58.8	95.0	28.3
Peru	Induction of labour for post-term pregnancies	2.1	3.1	95.0	1.5
Peru	Antenatal corticosteroids for preterm labour	86.4	95.0	95.0	61.1
Peru	Breastfeeding promotion	92.2	95.0	95.0	65.2
Peru	KMC - Kangaroo mother care	6.0	8.9	95.0	4.3
Peru	Oral antibiotics for neonatal sepsis	0.0	0.0	0.0	0.0
Peru	Injectable antibiotics for neonatal sepsis	95.0	95.0	95.0	85.4
Philippines	Folic acid supplementation/fortification	5.7	8.4	95.0	4.1
Philippines	Safe abortion services	51.9	76.2	95.0	36.7
Philippines	Post abortion case management	77.6	95.0	95.0	54.8
Philippines	Ectopic pregnancy case management	77.6	95.0	95.0	54.8
Philippines	TT - Tetanus toxoid vaccination	95.0	95.0	95.0	84.7
Philippines	IPTp	0.0	0.0	95.0	0.0
Philippines	Syphilis detection and treatment	30.7	45.1	95.0	21.7
Philippines	Iron supplementation in pregnancy	67.3	95.0	95.0	47.6
Philippines	Hypertensive disorder case management	27.6	40.4	95.0	19.5
Philippines	Diabetes case management	21.6	31.6	95.0	15.2
Philippines	Malaria case management	88.9	95.0	95.0	62.9
Philippines	MgSO4 management of pre-eclampsia	54.6	80.1	95.0	38.6
Philippines	Clean birth environment	84.9	95.0	95.0	60.0
Philippines	Immediate drying and additional stimulation	94.8	95.0	95.0	67.0
Philippines	Thermal protection	95.0	95.0	95.0	72.3
Philippines	Clean cord care	95.0	95.0	95.0	69.8
Philippines	Manual removal of placenta	38.7	56.8	95.0	27.4
Philippines	Parenteral administration of anti-convulsants	74.0	95.0	95.0	52.3
Philippines	Antibiotics for preterm or prolonged PROM	77.5	95.0	95.0	54.8
Philippines	Parenteral administration of antibiotics	77.5	95.0	95.0	54.8
Philippines	Assisted vaginal delivery	26.2	38.5	95.0	18.5
Philippines	Neonatal resuscitation	57.0	83.6	95.0	40.3
Philippines	Parenteral administration of uterotonics	92.5	95.0	95.0	65.4
Philippines	Removal of retained products of conception	34.3	50.4	95.0	24.3
Philippines	Induction of labour for post-term pregnancies	1.9	2.7	95.0	1.3
Philippines	Antenatal corticosteroids for preterm labour	74.0	95.0	95.0	52.3
Philippines	Breastfeeding promotion	42.3	62.1	95.0	29.9
Philippines	KMC - Kangaroo mother care	5.2	7.6	95.0	3.7
Philippines	Oral antibiotics for neonatal sepsis	0.0	0.0	0.0	0.0
Philippines	Injectable antibiotics for neonatal sepsis	95.0	95.0	95.0	73.1
Rwanda	Folic acid supplementation/fortification	2.9	4.3	95.0	2.1
Rwanda	Safe abortion services	4.4	6.4	95.0	3.1
Rwanda	Post abortion case management	90.5	95.0	95.0	64.0
Rwanda	Ectopic pregnancy case management	90.5	95.0	95.0	64.0
Rwanda	TT - Tetanus toxoid vaccination	95.0	95.0	95.0	84.7
Rwanda	IPTp	0.0	0.0	95.0	0.0
Rwanda	Syphilis detection and treatment	69.2	95.0	95.0	48.9
Rwanda	Iron supplementation in pregnancy	4.5	6.6	95.0	3.2
Rwanda	Hypertensive disorder case management	2.5	3.7	95.0	1.8
Rwanda	Diabetes case management	11.0	16.2	95.0	7.8
Rwanda	Malaria case management	45.8	67.2	95.0	32.4
Rwanda	MgSO4 management of pre-eclampsia	10.2	15.0	95.0	7.2
Rwanda	Clean birth environment	95.0	95.0	95.0	74.0
Rwanda	Immediate drying and additional stimulation	95.0	95.0	95.0	69.1
Rwanda	Thermal protection	95.0	95.0	95.0	84.3
Rwanda	Clean cord care	95.0	95.0	95.0	76.6
Rwanda	Manual removal of placenta	34.2	50.2	95.0	24.2
Rwanda	Parenteral administration of anti-convulsants	80.0	95.0	95.0	56.6
Rwanda	Antibiotics for preterm or prolonged PROM	48.8	71.7	95.0	34.5
Rwanda	Parenteral administration of antibiotics	48.8	71.7	95.0	34.5
Rwanda	Assisted vaginal delivery	27.0	39.6	95.0	19.1
Rwanda	Neonatal resuscitation	48.2	70.7	95.0	34.1
Rwanda	Parenteral administration of uterotonics	44.6	65.4	95.0	31.5
Rwanda	Removal of retained products of conception	39.9	58.6	95.0	28.2
Rwanda	Induction of labour for post-term pregnancies	2.1	3.1	95.0	1.5
Rwanda	Antenatal corticosteroids for preterm labour	80.0	95.0	95.0	56.6
Rwanda	Breastfeeding promotion	95.0	95.0	95.0	81.1
Rwanda	KMC - Kangaroo mother care	6.0	8.9	95.0	4.3

Country	Intervention	10% Increase	25% increase	95% target	2% Attrition
Rwanda	Oral antibiotics for neonatal sepsis	0.0	0.0	0.0	0.0
Rwanda	Injectable antibiotics for neonatal sepsis	95.0	95.0	95.0	85.4
São Tomé and Principe	Folic acid supplementation/fortification	5.6	8.2	95.0	3.9
São Tomé and Principe	Safe abortion services	0.0	0.0	95.0	0.0
São Tomé and Principe	Post abortion case management	90.8	95.0	95.0	64.2
São Tomé and Principe	Ectopic pregnancy case management	90.8	95.0	95.0	64.2
São Tomé and Principe	TT - Tetanus toxoid vaccination	99.0	99.0	99.0	93.2
São Tomé and Principe	IPTp	75.7	95.0	95.0	53.6
São Tomé and Principe	Syphilis detection and treatment	32.1	47.1	95.0	22.7
São Tomé and Principe	Iron supplementation in pregnancy	71.2	95.0	95.0	50.4
São Tomé and Principe	Hypertensive disorder case management	26.8	39.3	95.0	18.9
São Tomé and Principe	Diabetes case management	20.9	30.7	95.0	14.8
São Tomé and Principe	Malaria case management	86.2	95.0	95.0	61.0
São Tomé and Principe	MgSO4 management of pre-eclampsia	53.0	77.7	95.0	37.5
São Tomé and Principe	Clean birth environment	95.0	95.0	95.0	70.2
São Tomé and Principe	Immediate drying and additional stimulation	95.0	95.0	95.0	78.4
São Tomé and Principe	Thermal protection	95.0	95.0	95.0	84.6
São Tomé and Principe	Clean cord care	95.0	95.0	95.0	81.7
São Tomé and Principe	Manual removal of placenta	45.3	66.4	95.0	32.0
São Tomé and Principe	Parenteral administration of anti-convulsants	86.6	95.0	95.0	61.3
São Tomé and Principe	Antibiotics for preterm or prolonged PROM	90.6	95.0	95.0	64.1
São Tomé and Principe	Parenteral administration of antibiotics	90.6	95.0	95.0	64.1
São Tomé and Principe	Assisted vaginal delivery	30.6	44.9	95.0	21.6
São Tomé and Principe	Neonatal resuscitation	66.6	95.0	95.0	47.1
São Tomé and Principe	Parenteral administration of uterotonics	95.0	95.0	95.0	76.5
São Tomé and Principe	Removal of retained products of conception	40.1	58.8	95.0	28.3
São Tomé and Principe	Induction of labour for post-term pregnancies	2.1	3.1	95.0	1.5
São Tomé and Principe	Antenatal corticosteroids for preterm labour	86.6	95.0	95.0	61.3
São Tomé and Principe	Breastfeeding promotion	95.0	95.0	95.0	67.9
São Tomé and Principe	KMC - Kangaroo mother care	6.1	8.9	95.0	4.3
São Tomé and Principe	Oral antibiotics for neonatal sepsis	0.0	0.0	0.0	0.0
São Tomé and Principe	Injectable antibiotics for neonatal sepsis	95.0	95.0	95.0	85.6
Senegal	Folic acid supplementation/fortification	3.7	5.5	95.0	2.6
Senegal	Safe abortion services	0.0	0.0	95.0	0.0
Senegal	Post abortion case management	69.8	95.0	95.0	49.3
Senegal	Ectopic pregnancy case management	69.8	95.0	95.0	49.3
Senegal	TT - Tetanus toxoid vaccination	95.0	95.0	95.0	89.4
Senegal	IPTp	83.3	95.0	95.0	58.9
Senegal	Syphilis detection and treatment	31.9	46.9	95.0	22.6
Senegal	Iron supplementation in pregnancy	84.0	95.0	95.0	59.4
Senegal	Hypertensive disorder case management	17.8	26.2	95.0	12.6
Senegal	Diabetes case management	14.0	20.5	95.0	9.9
Senegal	Malaria case management	57.8	84.8	95.0	40.8
Senegal	MgSO4 management of pre-eclampsia	35.4	52.0	95.0	25.0
Senegal	Clean birth environment	91.2	95.0	95.0	64.5
Senegal	Immediate drying and additional stimulation	91.7	95.0	95.0	64.8
Senegal	Thermal protection	92.0	95.0	95.0	65.0
Senegal	Clean cord care	91.4	95.0	95.0	64.7
Senegal	Manual removal of placenta	47.9	70.3	95.0	33.9
Senegal	Parenteral administration of anti-convulsants	78.8	95.0	95.0	55.7
Senegal	Antibiotics for preterm or prolonged PROM	69.6	95.0	95.0	49.2
Senegal	Parenteral administration of antibiotics	69.6	95.0	95.0	49.2
Senegal	Assisted vaginal delivery	9.8	14.5	95.0	7.0
Senegal	Neonatal resuscitation	67.2	95.0	95.0	47.5
Senegal	Parenteral administration of uterotonics	81.3	95.0	95.0	57.5
Senegal	Removal of retained products of conception	46.7	68.6	95.0	33.0
Senegal	Induction of labour for post-term pregnancies	10.8	15.8	95.0	7.6
Senegal	Antenatal corticosteroids for preterm labour	78.8	95.0	95.0	55.7
Senegal	Breastfeeding promotion	47.8	70.1	95.0	33.8
Senegal	KMC - Kangaroo mother care	4.7	6.8	95.0	3.3
Senegal	Oral antibiotics for neonatal sepsis	0.0	0.0	0.0	0.0
Senegal	Injectable antibiotics for neonatal sepsis	93.0	95.0	95.0	65.8
Sierra Leone	Folic acid supplementation/fortification	5.2	7.6	95.0	3.6
Sierra Leone	Safe abortion services	0.0	0.0	95.0	0.0
Sierra Leone	Post abortion case management	76.6	95.0	95.0	54.1

Country	Intervention	10% Increase	25% increase	95% target	2% Attrition
Sierra Leone	Ectopic pregnancy case management	76.6	95.0	95.0	54.1
Sierra Leone	TT - Tetanus toxoid vaccination	95.0	95.0	95.0	84.7
Sierra Leone	IPTp	91.4	95.0	95.0	64.7
Sierra Leone	Syphilis detection and treatment	24.2	35.5	95.0	17.1
Sierra Leone	Iron supplementation in pregnancy	39.9	58.6	95.0	28.2
Sierra Leone	Hypertensive disorder case management	55.1	80.9	95.0	39.0
Sierra Leone	Diabetes case management	15.4	22.7	95.0	10.9
Sierra Leone	Malaria case management	94.5	95.0	95.0	66.8
Sierra Leone	MgSO4 management of pre-eclampsia	90.6	95.0	95.0	64.1
Sierra Leone	Clean birth environment	87.0	95.0	95.0	61.6
Sierra Leone	Immediate drying and additional stimulation	95.0	95.0	95.0	67.2
Sierra Leone	Thermal protection	95.0	95.0	95.0	71.3
Sierra Leone	Clean cord care	93.0	95.0	95.0	65.8
Sierra Leone	Manual removal of placenta	21.6	31.6	95.0	15.2
Sierra Leone	Parenteral administration of anti-convulsants	90.2	95.0	95.0	63.8
Sierra Leone	Antibiotics for preterm or prolonged PROM	88.2	95.0	95.0	62.4
Sierra Leone	Parenteral administration of antibiotics	88.2	95.0	95.0	62.4
Sierra Leone	Assisted vaginal delivery	58.0	85.2	95.0	41.0
Sierra Leone	Neonatal resuscitation	43.3	63.5	95.0	30.6
Sierra Leone	Parenteral administration of uterotonics	92.6	95.0	95.0	65.5
Sierra Leone	Removal of retained products of conception	45.9	67.4	95.0	32.5
Sierra Leone	Induction of labour for post-term pregnancies	1.1	1.6	95.0	0.8
Sierra Leone	Antenatal corticosteroids for preterm labour	90.2	95.0	95.0	63.8
Sierra Leone	Breastfeeding promotion	40.3	59.2	95.0	28.5
Sierra Leone	KMC - Kangaroo mother care	5.1	7.5	95.0	3.6
Sierra Leone	Oral antibiotics for neonatal sepsis	0.0	0.0	0.0	0.0
Sierra Leone	Injectable antibiotics for neonatal sepsis	95.0	95.0	95.0	72.2
Solomon Islands	Folic acid supplementation/fortification	4.3	6.3	95.0	3.0
Solomon Islands	Safe abortion services	95.0	95.0	95.0	80.0
Solomon Islands	Post abortion case management	84.4	95.0	95.0	59.6
Solomon Islands	Ectopic pregnancy case management	84.4	95.0	95.0	59.6
Solomon Islands	TT - Tetanus toxoid vaccination	95.0	95.0	95.0	80.0
Solomon Islands	IPTp	0.0	0.0	95.0	0.0
Solomon Islands	Syphilis detection and treatment	26.4	38.7	95.0	18.6
Solomon Islands	Iron supplementation in pregnancy	0.0	0.0	95.0	0.0
Solomon Islands	Hypertensive disorder case management	20.6	30.3	95.0	14.6
Solomon Islands	Diabetes case management	16.1	23.6	95.0	11.4
Solomon Islands	Malaria case management	66.7	95.0	95.0	47.2
Solomon Islands	MgSO4 management of pre-eclampsia	40.9	60.0	95.0	28.9
Solomon Islands	Clean birth environment	92.2	95.0	95.0	65.2
Solomon Islands	Immediate drying and additional stimulation	95.0	95.0	95.0	72.8
Solomon Islands	Thermal protection	95.0	95.0	95.0	78.6
Solomon Islands	Clean cord care	95.0	95.0	95.0	75.9
Solomon Islands	Manual removal of placenta	42.1	61.7	95.0	29.7
Solomon Islands	Parenteral administration of anti-convulsants	80.4	95.0	95.0	56.8
Solomon Islands	Antibiotics for preterm or prolonged PROM	84.1	95.0	95.0	59.5
Solomon Islands	Parenteral administration of antibiotics	84.1	95.0	95.0	59.5
Solomon Islands	Assisted vaginal delivery	28.5	41.8	95.0	20.1
Solomon Islands	Neonatal resuscitation	61.9	90.8	95.0	43.8
Solomon Islands	Parenteral administration of uterotonics	95.0	95.0	95.0	71.1
Solomon Islands	Removal of retained products of conception	37.3	54.7	95.0	26.4
Solomon Islands	Induction of labour for post-term pregnancies	2.0	2.9	95.0	1.4
Solomon Islands	Antenatal corticosteroids for preterm labour	80.4	95.0	95.0	56.8
Solomon Islands	Breastfeeding promotion	0.0	0.0	95.0	0.0
Solomon Islands	KMC - Kangaroo mother care	5.6	8.3	95.0	4.0
Solomon Islands	Oral antibiotics for neonatal sepsis	0.0	0.0	0.0	0.0
Solomon Islands	Injectable antibiotics for neonatal sepsis	95.0	95.0	95.0	79.5
Somalia	Folic acid supplementation/fortification	0.1	0.2	95.0	0.1
Somalia	Safe abortion services	4.4	6.4	95.0	3.1
Somalia	Post abortion case management	1.3	1.8	95.0	0.9
Somalia	Ectopic pregnancy case management	1.3	1.8	95.0	0.9
Somalia	TT - Tetanus toxoid vaccination	89.2	95.0	95.0	63.1
Somalia	IPTp	0.0	0.0	95.0	0.0
Somalia	Syphilis detection and treatment	8.7	12.7	95.0	6.1
Somalia	Iron supplementation in pregnancy	0.0	0.0	95.0	0.0

Country	Intervention	10% Increase	25% increase	95% target	2% Attrition
Somalia	Hypertensive disorder case management	0.7	1.0	95.0	0.5
Somalia	Diabetes case management	0.5	0.8	95.0	0.4
Somalia	Malaria case management	2.0	2.9	95.0	1.4
Somalia	MgSO4 management of pre-eclampsia	1.2	1.8	95.0	0.8
Somalia	Clean birth environment	10.2	15.0	95.0	7.2
Somalia	Immediate drying and additional stimulation	11.4	16.8	95.0	8.1
Somalia	Thermal protection	12.4	18.2	95.0	8.8
Somalia	Clean cord care	11.8	17.4	95.0	8.4
Somalia	Manual removal of placenta	4.7	6.8	95.0	3.3
Somalia	Parenteral administration of anti-convulsants	8.9	13.1	95.0	6.3
Somalia	Antibiotics for preterm or prolonged PROM	9.3	13.7	95.0	6.6
Somalia	Parenteral administration of antibiotics	9.3	13.7	95.0	6.6
Somalia	Assisted vaginal delivery	3.2	4.7	95.0	2.3
Somalia	Neonatal resuscitation	6.8	10.0	95.0	4.8
Somalia	Parenteral administration of uterotonics	11.2	16.4	95.0	7.9
Somalia	Removal of retained products of conception	4.1	6.1	95.0	2.9
Somalia	Induction of labour for post-term pregnancies	0.3	0.4	95.0	0.2
Somalia	Antenatal corticosteroids for preterm labour	8.9	13.1	95.0	6.3
Somalia	Breastfeeding promotion	11.7	17.2	95.0	8.3
Somalia	KMC - Kangaroo mother care	0.6	0.9	95.0	0.4
Somalia	Oral antibiotics for neonatal sepsis	0.0	0.0	0.0	0.0
Somalia	Injectable antibiotics for neonatal sepsis	12.5	18.4	95.0	8.8
South Africa	Folic acid supplementation/fortification	5.0	7.4	95.0	3.6
South Africa	Safe abortion services	56.6	83.0	95.0	40.0
South Africa	Post abortion case management	95.0	95.0	95.0	67.7
South Africa	Ectopic pregnancy case management	95.0	95.0	95.0	67.7
South Africa	TT - Tetanus toxoid vaccination	95.0	95.0	95.0	84.7
South Africa	IPTp	0.0	0.0	95.0	0.0
South Africa	Syphilis detection and treatment	29.9	43.9	95.0	21.2
South Africa	Iron supplementation in pregnancy	67.2	95.0	95.0	47.5
South Africa	Hypertensive disorder case management	24.1	35.4	95.0	17.0
South Africa	Diabetes case management	18.8	27.5	95.0	13.3
South Africa	Malaria case management	77.9	95.0	95.0	55.1
South Africa	MgSO4 management of pre-eclampsia	47.8	70.1	95.0	33.8
South Africa	Clean birth environment	95.0	95.0	95.0	74.1
South Africa	Immediate drying and additional stimulation	95.0	95.0	95.0	82.6
South Africa	Thermal protection	95.0	95.0	95.0	89.2
South Africa	Clean cord care	95.0	95.0	95.0	86.1
South Africa	Manual removal of placenta	47.8	70.1	95.0	33.8
South Africa	Parenteral administration of anti-convulsants	91.3	95.0	95.0	64.6
South Africa	Antibiotics for preterm or prolonged PROM	95.0	95.0	95.0	67.6
South Africa	Parenteral administration of antibiotics	95.0	95.0	95.0	67.6
South Africa	Assisted vaginal delivery	32.2	47.3	95.0	22.8
South Africa	Neonatal resuscitation	70.1	95.0	95.0	49.6
South Africa	Parenteral administration of uterotonics	95.0	95.0	95.0	80.7
South Africa	Removal of retained products of conception	42.3	62.1	95.0	29.9
South Africa	Induction of labour for post-term pregnancies	2.3	3.3	95.0	1.6
South Africa	Antenatal corticosteroids for preterm labour	91.3	95.0	95.0	64.6
South Africa	Breastfeeding promotion	8.1	11.9	95.0	5.7
South Africa	KMC - Kangaroo mother care	6.4	9.4	95.0	4.5
South Africa	Oral antibiotics for neonatal sepsis	0.0	0.0	0.0	0.0
South Africa	Injectable antibiotics for neonatal sepsis	95.9	95.9	95.9	90.3
South Sudan	Folic acid supplementation/fortification	1.2	1.7	95.0	0.8
South Sudan	Safe abortion services	2.9	4.3	95.0	2.1
South Sudan	Post abortion case management	1.6	2.3	95.0	1.1
South Sudan	Ectopic pregnancy case management	1.6	2.3	95.0	1.1
South Sudan	TT - Tetanus toxoid vaccination	90.5	95.0	95.0	64.0
South Sudan	IPTp	13.6	19.9	95.0	9.6
South Sudan	Syphilis detection and treatment	13.3	19.5	95.0	9.4
South Sudan	Iron supplementation in pregnancy	0.0	0.0	95.0	0.0
South Sudan	Hypertensive disorder case management	5.6	8.2	95.0	4.0
South Sudan	Diabetes case management	4.3	6.3	95.0	3.0
South Sudan	Malaria case management	17.8	26.2	95.0	12.6
South Sudan	MgSO4 management of pre-eclampsia	10.9	16.0	95.0	7.7
South Sudan	Clean birth environment	12.8	18.8	95.0	9.0

Country	Intervention	10% Increase	25% increase	95% target	2% Attrition
South Sudan	Immediate drying and additional stimulation	14.2	20.9	95.0	10.1
South Sudan	Thermal protection	15.3	22.5	95.0	10.8
South Sudan	Clean cord care	14.8	21.7	95.0	10.4
South Sudan	Manual removal of placenta	5.9	8.6	95.0	4.1
South Sudan	Parenteral administration of anti-convulsants	11.0	16.2	95.0	7.8
South Sudan	Antibiotics for preterm or prolonged PROM	11.6	17.0	95.0	8.2
South Sudan	Parenteral administration of antibiotics	11.6	17.0	95.0	8.2
South Sudan	Assisted vaginal delivery	3.9	5.7	95.0	2.7
South Sudan	Neonatal resuscitation	8.5	12.5	95.0	6.0
South Sudan	Parenteral administration of uterotonics	13.8	20.3	95.0	9.8
South Sudan	Removal of retained products of conception	5.2	7.6	95.0	3.7
South Sudan	Induction of labour for post-term pregnancies	0.3	0.4	95.0	0.2
South Sudan	Antenatal corticosteroids for preterm labour	11.0	16.2	95.0	7.8
South Sudan	Breastfeeding promotion	54.7	80.3	95.0	38.7
South Sudan	KMC - Kangaroo mother care	0.8	1.1	95.0	0.6
South Sudan	Oral antibiotics for neonatal sepsis	0.0	0.0	0.0	0.0
South Sudan	Injectable antibiotics for neonatal sepsis	15.6	22.9	95.0	11.0
Sudan	Folic acid supplementation/fortification	3.4	5.0	95.0	2.4
Sudan	Safe abortion services	2.9	4.3	95.0	2.1
Sudan	Post abortion case management	3.7	5.4	95.0	2.6
Sudan	Ectopic pregnancy case management	3.7	5.4	95.0	2.6
Sudan	TT - Tetanus toxoid vaccination	95.0	95.0	95.0	75.3
Sudan	IPTp	0.0	0.0	95.0	0.0
Sudan	Syphilis detection and treatment	26.1	38.3	95.0	18.4
Sudan	Iron supplementation in pregnancy	0.0	0.0	95.0	0.0
Sudan	Hypertensive disorder case management	16.2	23.8	95.0	11.5
Sudan	Diabetes case management	12.6	18.6	95.0	8.9
Sudan	Malaria case management	52.3	76.8	95.0	37.0
Sudan	MgSO4 management of pre-eclampsia	32.1	47.1	95.0	22.7
Sudan	Clean birth environment	30.2	44.3	95.0	21.4
Sudan	Immediate drying and additional stimulation	33.8	49.6	95.0	23.9
Sudan	Thermal protection	36.5	53.5	95.0	25.8
Sudan	Clean cord care	35.3	51.8	95.0	24.9
Sudan	Manual removal of placenta	13.8	20.3	95.0	9.8
Sudan	Parenteral administration of anti-convulsants	26.4	38.7	95.0	18.6
Sudan	Antibiotics for preterm or prolonged PROM	27.6	40.4	95.0	19.5
Sudan	Parenteral administration of antibiotics	27.6	40.4	95.0	19.5
Sudan	Assisted vaginal delivery	9.3	13.7	95.0	6.6
Sudan	Neonatal resuscitation	20.2	29.7	95.0	14.3
Sudan	Parenteral administration of uterotonics	33.0	48.4	95.0	23.3
Sudan	Removal of retained products of conception	12.2	18.0	95.0	8.7
Sudan	Induction of labour for post-term pregnancies	0.7	1.0	95.0	0.5
Sudan	Antenatal corticosteroids for preterm labour	26.4	38.7	95.0	18.6
Sudan	Breastfeeding promotion	70.5	95.0	95.0	49.9
Sudan	KMC - Kangaroo mother care	1.8	2.7	95.0	1.3
Sudan	Oral antibiotics for neonatal sepsis	0.0	0.0	0.0	0.0
Sudan	Injectable antibiotics for neonatal sepsis	36.9	54.1	95.0	26.1
Suriname	Folic acid supplementation/fortification	4.4	6.5	95.0	3.1
Suriname	Safe abortion services	0.3	0.4	95.0	0.2
Suriname	Post abortion case management	92.1	95.0	95.0	65.2
Suriname	Ectopic pregnancy case management	92.1	95.0	95.0	65.2
Suriname	TT - Tetanus toxoid vaccination	95.0	95.0	95.0	87.5
Suriname	IPTp	0.0	0.0	95.0	0.0
Suriname	Syphilis detection and treatment	31.3	45.9	95.0	22.1
Suriname	Iron supplementation in pregnancy	0.0	0.0	95.0	0.0
Suriname	Hypertensive disorder case management	21.3	31.3	95.0	15.1
Suriname	Diabetes case management	16.6	24.4	95.0	11.8
Suriname	Malaria case management	68.9	95.0	95.0	48.8
Suriname	MgSO4 management of pre-eclampsia	42.2	61.9	95.0	29.8
Suriname	Clean birth environment	95.0	95.0	95.0	71.2
Suriname	Immediate drying and additional stimulation	95.0	95.0	95.0	79.5
Suriname	Thermal protection	95.0	95.0	95.0	85.8
Suriname	Clean cord care	95.0	95.0	95.0	82.9
Suriname	Manual removal of placenta	45.9	67.4	95.0	32.5
Suriname	Parenteral administration of anti-convulsants	87.8	95.0	95.0	62.1

Country	Intervention	10% Increase	25% increase	95% target	2% Attrition
Suriname	Antibiotics for preterm or prolonged PROM	92.0	95.0	95.0	65.0
Suriname	Parenteral administration of antibiotics	92.0	95.0	95.0	65.0
Suriname	Assisted vaginal delivery	31.0	45.5	95.0	21.9
Suriname	Neonatal resuscitation	67.5	95.0	95.0	47.7
Suriname	Parenteral administration of uterotonics	95.0	95.0	95.0	77.6
Suriname	Removal of retained products of conception	40.7	59.8	95.0	28.8
Suriname	Induction of labour for post-term pregnancies	2.1	3.1	95.0	1.5
Suriname	Antenatal corticosteroids for preterm labour	87.8	95.0	95.0	62.1
Suriname	Breastfeeding promotion	1.1	1.6	95.0	0.8
Suriname	KMC - Kangaroo mother care	6.1	9.0	95.0	4.3
Suriname	Oral antibiotics for neonatal sepsis	0.0	0.0	0.0	0.0
Suriname	Injectable antibiotics for neonatal sepsis	95.0	95.0	95.0	86.9
Tajikistan	Folic acid supplementation/fortification	4.3	6.3	95.0	3.0
Tajikistan	Safe abortion services	46.7	68.6	95.0	33.0
Tajikistan	Post abortion case management	88.0	95.0	95.0	62.3
Tajikistan	Ectopic pregnancy case management	88.0	95.0	95.0	62.3
Tajikistan	TT - Tetanus toxoid vaccination	0.0	0.0	95.0	0.0
Tajikistan	IPTp	0.0	0.0	95.0	0.0
Tajikistan	Syphilis detection and treatment	30.2	44.3	95.0	21.4
Tajikistan	Iron supplementation in pregnancy	1.2	1.8	95.0	0.8
Tajikistan	Hypertensive disorder case management	20.5	30.1	95.0	14.5
Tajikistan	Diabetes case management	16.0	23.4	95.0	11.3
Tajikistan	Malaria case management	66.3	95.0	95.0	46.9
Tajikistan	MgSO4 management of pre-eclampsia	40.6	59.6	95.0	28.7
Tajikistan	Clean birth environment	95.0	95.0	95.0	68.0
Tajikistan	Immediate drying and additional stimulation	95.0	95.0	95.0	76.0
Tajikistan	Thermal protection	95.0	95.0	95.0	82.1
Tajikistan	Clean cord care	95.0	95.0	95.0	79.2
Tajikistan	Manual removal of placenta	43.9	64.5	95.0	31.1
Tajikistan	Parenteral administration of anti-convulsants	84.0	95.0	95.0	59.4
Tajikistan	Antibiotics for preterm or prolonged PROM	87.8	95.0	95.0	62.1
Tajikistan	Parenteral administration of antibiotics	87.8	95.0	95.0	62.1
Tajikistan	Assisted vaginal delivery	29.7	43.6	95.0	21.0
Tajikistan	Neonatal resuscitation	64.6	94.7	95.0	45.6
Tajikistan	Parenteral administration of uterotonics	95.0	95.0	95.0	74.2
Tajikistan	Removal of retained products of conception	38.9	57.0	95.0	27.5
Tajikistan	Induction of labour for post-term pregnancies	2.1	3.1	95.0	1.5
Tajikistan	Antenatal corticosteroids for preterm labour	84.0	95.0	95.0	59.4
Tajikistan	Breastfeeding promotion	42.1	61.7	95.0	29.7
Tajikistan	KMC - Kangaroo mother care	5.9	8.6	95.0	4.2
Tajikistan	Oral antibiotics for neonatal sepsis	0.0	0.0	0.0	0.0
Tajikistan	Injectable antibiotics for neonatal sepsis	95.0	95.0	95.0	83.0
Timor-Leste	Folic acid supplementation/fortification	5.1	7.5	95.0	3.6
Timor-Leste	Safe abortion services	51.9	76.2	95.0	36.7
Timor-Leste	Post abortion case management	32.3	47.4	95.0	22.8
Timor-Leste	Ectopic pregnancy case management	32.3	47.4	95.0	22.8
Timor-Leste	TT - Tetanus toxoid vaccination	95.0	95.0	95.0	78.1
Timor-Leste	IPTp	0.0	0.0	95.0	0.0
Timor-Leste	Syphilis detection and treatment	27.8	40.8	95.0	19.7
Timor-Leste	Iron supplementation in pregnancy	17.2	25.2	95.0	12.1
Timor-Leste	Hypertensive disorder case management	24.6	36.1	95.0	17.4
Timor-Leste	Diabetes case management	19.2	28.1	95.0	13.6
Timor-Leste	Malaria case management	79.3	95.0	95.0	56.1
Timor-Leste	MgSO4 management of pre-eclampsia	48.6	71.3	95.0	34.4
Timor-Leste	Clean birth environment	53.0	77.7	95.0	37.5
Timor-Leste	Immediate drying and additional stimulation	59.1	86.7	95.0	41.8
Timor-Leste	Thermal protection	63.9	93.8	95.0	45.2
Timor-Leste	Clean cord care	61.6	90.4	95.0	43.6
Timor-Leste	Manual removal of placenta	24.1	35.4	95.0	17.0
Timor-Leste	Parenteral administration of anti-convulsants	46.2	67.8	95.0	32.7
Timor-Leste	Antibiotics for preterm or prolonged PROM	48.3	70.9	95.0	34.2
Timor-Leste	Parenteral administration of antibiotics	48.3	70.9	95.0	34.2
Timor-Leste	Assisted vaginal delivery	16.4	24.0	95.0	11.6
Timor-Leste	Neonatal resuscitation	35.5	52.1	95.0	25.1
Timor-Leste	Parenteral administration of uterotonics	57.8	84.8	95.0	40.8

Country	Intervention	10% Increase	25% increase	95% target	2% Attrition
Timor-Leste	Removal of retained products of conception	21.4	31.4	95.0	15.2
Timor-Leste	Induction of labour for post-term pregnancies	1.2	1.8	95.0	0.8
Timor-Leste	Antenatal corticosteroids for preterm labour	46.2	67.8	95.0	32.7
Timor-Leste	Breastfeeding promotion	65.2	95.0	95.0	46.1
Timor-Leste	KMC - Kangaroo mother care	3.2	4.7	95.0	2.3
Timor-Leste	Oral antibiotics for neonatal sepsis	0.0	0.0	0.0	0.0
Timor-Leste	Injectable antibiotics for neonatal sepsis	64.6	94.7	95.0	45.6
Togo	Folic acid supplementation/fortification	3.8	5.5	95.0	2.7
Togo	Safe abortion services	0.0	0.0	95.0	0.0
Togo	Post abortion case management	61.3	89.9	95.0	43.3
Togo	Ectopic pregnancy case management	61.3	89.9	95.0	43.3
Togo	TT - Tetanus toxoid vaccination	95.0	95.0	95.0	78.1
Togo	IPTp	90.8	95.0	95.0	64.2
Togo	Syphilis detection and treatment	24.5	35.9	95.0	17.3
Togo	Iron supplementation in pregnancy	49.4	72.5	95.0	34.9
Togo	Hypertensive disorder case management	22.2	32.6	95.0	15.7
Togo	Diabetes case management	24.4	35.7	95.0	17.2
Togo	Malaria case management	65.4	95.0	95.0	46.2
Togo	MgSO4 management of pre-eclampsia	12.6	18.6	95.0	8.9
Togo	Clean birth environment	57.0	83.6	95.0	40.3
Togo	Immediate drying and additional stimulation	73.7	95.0	95.0	52.1
Togo	Thermal protection	80.8	95.0	95.0	57.1
Togo	Clean cord care	61.8	90.6	95.0	43.7
Togo	Manual removal of placenta	48.0	70.5	95.0	34.0
Togo	Parenteral administration of anti-convulsants	14.5	21.3	95.0	10.3
Togo	Antibiotics for preterm or prolonged PROM	64.6	94.7	95.0	45.6
Togo	Parenteral administration of antibiotics	64.6	94.7	95.0	45.6
Togo	Assisted vaginal delivery	10.8	15.8	95.0	7.6
Togo	Neonatal resuscitation	17.0	25.0	95.0	12.0
Togo	Parenteral administration of uterotonics	73.7	95.0	95.0	52.1
Togo	Removal of retained products of conception	22.5	33.0	95.0	15.9
Togo	Induction of labour for post-term pregnancies	1.5	2.1	95.0	1.0
Togo	Antenatal corticosteroids for preterm labour	14.5	21.3	95.0	10.3
Togo	Breastfeeding promotion	74.5	95.0	95.0	52.7
Togo	KMC - Kangaroo mother care	4.1	6.0	95.0	2.9
Togo	Oral antibiotics for neonatal sepsis	0.0	0.0	0.0	0.0
Togo	Injectable antibiotics for neonatal sepsis	81.7	95.0	95.0	57.8
Turkmenistan	Folic acid supplementation/fortification	6.4	9.4	95.0	4.5
Turkmenistan	Safe abortion services	46.7	68.6	95.0	33.0
Turkmenistan	Post abortion case management	95.0	95.0	95.0	70.2
Turkmenistan	Ectopic pregnancy case management	95.0	95.0	95.0	70.2
Turkmenistan	TT - Tetanus toxoid vaccination	0.0	0.0	95.0	0.0
Turkmenistan	IPTp	0.0	0.0	95.0	0.0
Turkmenistan	Syphilis detection and treatment	32.9	48.2	95.0	23.2
Turkmenistan	Iron supplementation in pregnancy	0.0	0.0	95.0	0.0
Turkmenistan	Hypertensive disorder case management	30.7	45.1	95.0	21.7
Turkmenistan	Diabetes case management	24.0	35.2	95.0	16.9
Turkmenistan	Malaria case management	95.0	95.0	95.0	70.2
Turkmenistan	MgSO4 management of pre-eclampsia	60.8	89.3	95.0	43.0
Turkmenistan	Clean birth environment	95.0	95.0	95.0	76.7
Turkmenistan	Immediate drying and additional stimulation	95.0	95.0	95.0	85.6
Turkmenistan	Thermal protection	98.3	98.3	98.3	92.5
Turkmenistan	Clean cord care	95.0	95.0	95.0	89.3
Turkmenistan	Manual removal of placenta	49.5	72.7	95.0	35.0
Turkmenistan	Parenteral administration of anti-convulsants	94.6	95.0	95.0	66.9
Turkmenistan	Antibiotics for preterm or prolonged PROM	95.0	95.0	95.0	70.0
Turkmenistan	Parenteral administration of antibiotics	95.0	95.0	95.0	70.0
Turkmenistan	Assisted vaginal delivery	33.4	49.0	95.0	23.6
Turkmenistan	Neonatal resuscitation	72.8	95.0	95.0	51.5
Turkmenistan	Parenteral administration of uterotonics	95.0	95.0	95.0	83.6
Turkmenistan	Removal of retained products of conception	43.8	64.3	95.0	31.0
Turkmenistan	Induction of labour for post-term pregnancies	2.3	3.3	95.0	1.6
Turkmenistan	Antenatal corticosteroids for preterm labour	94.6	95.0	95.0	66.9
Turkmenistan	Breastfeeding promotion	72.5	95.0	95.0	51.3
Turkmenistan	KMC - Kangaroo mother care	6.6	9.7	95.0	4.7

Country	Intervention	10% Increase	25% increase	95% target	2% Attrition
Turkmenistan	Oral antibiotics for neonatal sepsis	0.0	0.0	0.0	0.0
Turkmenistan	Injectable antibiotics for neonatal sepsis	99.4	99.4	99.4	93.6
Uganda	Folic acid supplementation/fortification	4.0	5.9	95.0	2.8
Uganda	Safe abortion services	4.4	6.4	95.0	3.1
Uganda	Post abortion case management	73.3	95.0	95.0	51.8
Uganda	Ectopic pregnancy case management	73.3	95.0	95.0	51.8
Uganda	TT - Tetanus toxoid vaccination	95.0	95.0	95.0	80.0
Uganda	IPTp	61.1	89.6	95.0	43.2
Uganda	Syphilis detection and treatment	37.4	54.9	95.0	26.4
Uganda	Iron supplementation in pregnancy	30.1	44.1	95.0	21.3
Uganda	Hypertensive disorder case management	23.6	34.6	95.0	16.7
Uganda	Diabetes case management	13.4	19.7	95.0	9.5
Uganda	Malaria case management	54.0	79.3	95.0	38.2
Uganda	MgSO4 management of pre-eclampsia	30.7	45.1	95.0	21.7
Uganda	Clean birth environment	76.8	95.0	95.0	54.3
Uganda	Immediate drying and additional stimulation	75.6	95.0	95.0	53.5
Uganda	Thermal protection	95.0	95.0	95.0	68.2
Uganda	Clean cord care	84.7	95.0	95.0	59.9
Uganda	Manual removal of placenta	32.2	47.3	95.0	22.8
Uganda	Parenteral administration of anti-convulsants	54.4	79.9	95.0	38.5
Uganda	Antibiotics for preterm or prolonged PROM	66.6	95.0	95.0	47.1
Uganda	Parenteral administration of antibiotics	66.6	95.0	95.0	47.1
Uganda	Assisted vaginal delivery	34.1	50.0	95.0	24.1
Uganda	Neonatal resuscitation	48.2	70.7	95.0	34.1
Uganda	Parenteral administration of uterotonics	87.3	95.0	95.0	61.7
Uganda	Removal of retained products of conception	37.8	55.5	95.0	26.7
Uganda	Induction of labour for post-term pregnancies	15.3	22.5	95.0	10.8
Uganda	Antenatal corticosteroids for preterm labour	54.4	79.9	95.0	38.5
Uganda	Breastfeeding promotion	81.6	95.0	95.0	57.7
Uganda	KMC - Kangaroo mother care	4.9	7.2	95.0	3.5
Uganda	Oral antibiotics for neonatal sepsis	0.0	0.0	0.0	0.0
Uganda	Injectable antibiotics for neonatal sepsis	95.0	95.0	95.0	69.1
United Republic of Tanzania	Folic acid supplementation/fortification	3.3	4.8	95.0	2.3
United Republic of Tanzania	Safe abortion services	4.4	6.4	95.0	3.1
United Republic of Tanzania	Post abortion case management	62.5	91.7	95.0	44.2
United Republic of Tanzania	Ectopic pregnancy case management	62.5	91.7	95.0	44.2
United Republic of Tanzania	TT - Tetanus toxoid vaccination	95.0	95.0	95.0	84.7
United Republic of Tanzania	IPTp	74.7	95.0	95.0	52.8
United Republic of Tanzania	Syphilis detection and treatment	74.8	95.0	95.0	52.9
United Republic of Tanzania	Iron supplementation in pregnancy	28.5	41.8	95.0	20.1
United Republic of Tanzania	Hypertensive disorder case management	15.7	23.0	95.0	11.1
United Republic of Tanzania	Diabetes case management	12.2	18.0	95.0	8.7
United Republic of Tanzania	Malaria case management	56.3	82.6	95.0	39.8
United Republic of Tanzania	MgSO4 management of pre-eclampsia	46.3	68.0	95.0	32.8
United Republic of Tanzania	Clean birth environment	68.7	95.0	95.0	48.6
United Republic of Tanzania	Immediate drying and additional stimulation	76.4	95.0	95.0	54.0
United Republic of Tanzania	Thermal protection	82.1	95.0	95.0	58.1
United Republic of Tanzania	Clean cord care	80.9	95.0	95.0	57.2
United Republic of Tanzania	Manual removal of placenta	32.6	47.9	95.0	23.1
United Republic of Tanzania	Parenteral administration of anti-convulsants	66.4	95.0	95.0	47.0
United Republic of Tanzania	Antibiotics for preterm or prolonged PROM	42.9	62.9	95.0	30.3
United Republic of Tanzania	Parenteral administration of antibiotics	42.9	62.9	95.0	30.3
United Republic of Tanzania	Assisted vaginal delivery	21.0	30.9	95.0	14.9
United Republic of Tanzania	Neonatal resuscitation	71.3	95.0	95.0	50.4
United Republic of Tanzania	Parenteral administration of uterotonics	76.0	95.0	95.0	53.7
United Republic of Tanzania	Removal of retained products of conception	20.4	29.9	95.0	14.4
United Republic of Tanzania	Induction of labour for post-term pregnancies	8.7	12.7	95.0	6.1
United Republic of Tanzania	Antenatal corticosteroids for preterm labour	66.4	95.0	95.0	47.0
United Republic of Tanzania	Breastfeeding promotion	69.3	95.0	95.0	49.0
United Republic of Tanzania	KMC - Kangaroo mother care	4.2	6.1	95.0	2.9
United Republic of Tanzania	Oral antibiotics for neonatal sepsis	0.0	0.0	0.0	0.0
United Republic of Tanzania	Injectable antibiotics for neonatal sepsis	83.3	95.0	95.0	58.9
Uzbekistan	Folic acid supplementation/fortification	5.2	7.6	95.0	3.7
Uzbekistan	Safe abortion services	46.7	68.6	95.0	33.0
Uzbekistan	Post abortion case management	95.0	95.0	95.0	68.7

Country	Intervention	10% Increase	25% increase	95% target	2% Attrition
Uzbekistan	Ectopic pregnancy case management	95.0	95.0	95.0	68.7
Uzbekistan	TT - Tetanus toxoid vaccination	0.0	0.0	95.0	0.0
Uzbekistan	IPTp	0.0	0.0	95.0	0.0
Uzbekistan	Syphilis detection and treatment	32.6	47.9	95.0	23.1
Uzbekistan	Iron supplementation in pregnancy	0.0	0.0	95.0	0.0
Uzbekistan	Hypertensive disorder case management	25.0	36.7	95.0	17.7
Uzbekistan	Diabetes case management	19.6	28.7	95.0	13.8
Uzbekistan	Malaria case management	80.8	95.0	95.0	57.1
Uzbekistan	MgSO4 management of pre-eclampsia	49.5	72.7	95.0	35.0
Uzbekistan	Clean birth environment	95.0	95.0	95.0	75.1
Uzbekistan	Immediate drying and additional stimulation	95.0	95.0	95.0	83.9
Uzbekistan	Thermal protection	96.2	96.2	96.2	90.5
Uzbekistan	Clean cord care	95.0	95.0	95.0	87.4
Uzbekistan	Manual removal of placenta	48.4	71.1	95.0	34.3
Uzbekistan	Parenteral administration of anti-convulsants	92.6	95.0	95.0	65.5
Uzbekistan	Antibiotics for preterm or prolonged PROM	95.0	95.0	95.0	68.5
Uzbekistan	Parenteral administration of antibiotics	95.0	95.0	95.0	68.5
Uzbekistan	Assisted vaginal delivery	32.7	48.0	95.0	23.2
Uzbekistan	Neonatal resuscitation	71.2	95.0	95.0	50.4
Uzbekistan	Parenteral administration of uterotonics	95.0	95.0	95.0	81.9
Uzbekistan	Removal of retained products of conception	43.0	63.1	95.0	30.4
Uzbekistan	Induction of labour for post-term pregnancies	2.3	3.3	95.0	1.6
Uzbekistan	Antenatal corticosteroids for preterm labour	92.6	95.0	95.0	65.5
Uzbekistan	Breastfeeding promotion	31.7	46.5	95.0	22.4
Uzbekistan	KMC - Kangaroo mother care	6.5	9.5	95.0	4.6
Uzbekistan	Oral antibiotics for neonatal sepsis	0.0	0.0	0.0	0.0
Uzbekistan	Injectable antibiotics for neonatal sepsis	97.3	97.3	97.3	91.6
Venezuela	Folic acid supplementation/fortification	0.0	0.0	95.0	0.0
Venezuela	Safe abortion services	0.3	0.4	95.0	0.2
Venezuela	Post abortion case management	94.8	95.0	95.0	67.1
Venezuela	Ectopic pregnancy case management	94.8	95.0	95.0	67.1
Venezuela	TT - Tetanus toxoid vaccination	93.2	95.0	95.0	65.9
Venezuela	IPTp	0.0	0.0	95.0	0.0
Venezuela	Syphilis detection and treatment	0.0	0.0	95.0	0.0
Venezuela	Iron supplementation in pregnancy	0.0	0.0	95.0	0.0
Venezuela	Hypertensive disorder case management	0.0	0.0	95.0	0.0
Venezuela	Diabetes case management	0.0	0.0	95.0	0.0
Venezuela	Malaria case management	0.0	0.0	95.0	0.0
Venezuela	MgSO4 management of pre-eclampsia	0.0	0.0	95.0	0.0
Venezuela	Clean birth environment	95.0	95.0	95.0	73.3
Venezuela	Immediate drying and additional stimulation	95.0	95.0	95.0	81.9
Venezuela	Thermal protection	95.0	95.0	95.0	88.4
Venezuela	Clean cord care	95.0	95.0	95.0	85.4
Venezuela	Manual removal of placenta	47.3	69.3	95.0	33.4
Venezuela	Parenteral administration of anti-convulsants	90.4	95.0	95.0	63.9
Venezuela	Antibiotics for preterm or prolonged PROM	94.6	95.0	95.0	66.9
Venezuela	Parenteral administration of antibiotics	94.6	95.0	95.0	66.9
Venezuela	Assisted vaginal delivery	31.9	46.9	95.0	22.6
Venezuela	Neonatal resuscitation	69.5	95.0	95.0	49.1
Venezuela	Parenteral administration of uterotonics	95.0	95.0	95.0	79.9
Venezuela	Removal of retained products of conception	41.9	61.5	95.0	29.6
Venezuela	Induction of labour for post-term pregnancies	2.3	3.3	95.0	1.6
Venezuela	Antenatal corticosteroids for preterm labour	90.4	95.0	95.0	63.9
Venezuela	Breastfeeding promotion	0.0	0.0	95.0	0.0
Venezuela	KMC - Kangaroo mother care	6.3	9.3	95.0	4.5
Venezuela	Oral antibiotics for neonatal sepsis	0.0	0.0	0.0	0.0
Venezuela	Injectable antibiotics for neonatal sepsis	95.0	95.0	95.0	89.4
Viet Nam	Folic acid supplementation/fortification	4.9	7.2	95.0	3.5
Viet Nam	Safe abortion services	51.9	76.2	95.0	36.7
Viet Nam	Post abortion case management	93.4	95.0	95.0	66.1
Viet Nam	Ectopic pregnancy case management	93.4	95.0	95.0	66.1
Viet Nam	TT - Tetanus toxoid vaccination	95.0	95.0	95.0	88.5
Viet Nam	IPTp	0.0	0.0	95.0	0.0
Viet Nam	Syphilis detection and treatment	31.5	46.3	95.0	22.3
Viet Nam	Iron supplementation in pregnancy	0.0	0.0	95.0	0.0

Country	Intervention	10% Increase	25% increase	95% target	2% Attrition
Viet Nam	Hypertensive disorder case management	23.6	34.6	95.0	16.7
Viet Nam	Diabetes case management	18.4	27.0	95.0	13.0
Viet Nam	Malaria case management	76.0	95.0	95.0	53.7
Viet Nam	MgSO4 management of pre-eclampsia	46.6	68.4	95.0	32.9
Viet Nam	Clean birth environment	95.0	95.0	95.0	72.3
Viet Nam	Immediate drying and additional stimulation	95.0	95.0	95.0	80.7
Viet Nam	Thermal protection	95.0	95.0	95.0	87.1
Viet Nam	Clean cord care	95.0	95.0	95.0	84.1
Viet Nam	Manual removal of placenta	46.6	68.4	95.0	32.9
Viet Nam	Parenteral administration of anti-convulsants	89.2	95.0	95.0	63.1
Viet Nam	Antibiotics for preterm or prolonged PROM	93.3	95.0	95.0	66.0
Viet Nam	Parenteral administration of antibiotics	93.3	95.0	95.0	66.0
Viet Nam	Assisted vaginal delivery	31.5	46.3	95.0	22.3
Viet Nam	Neonatal resuscitation	68.5	95.0	95.0	48.5
Viet Nam	Parenteral administration of uterotonics	95.0	95.0	95.0	78.8
Viet Nam	Removal of retained products of conception	41.3	60.5	95.0	29.2
Viet Nam	Induction of labour for post-term pregnancies	2.1	3.1	95.0	1.5
Viet Nam	Antenatal corticosteroids for preterm labour	89.2	95.0	95.0	63.1
Viet Nam	Breastfeeding promotion	30.9	45.3	95.0	21.8
Viet Nam	KMC - Kangaroo mother care	6.2	9.1	95.0	4.4
Viet Nam	Oral antibiotics for neonatal sepsis	0.0	0.0	0.0	0.0
Viet Nam	Injectable antibiotics for neonatal sepsis	95.0	95.0	95.0	88.1
Yemen	Folic acid supplementation/fortification	1.6	2.4	95.0	1.2
Yemen	Safe abortion services	53.2	78.1	95.0	37.6
Yemen	Post abortion case management	20.4	30.0	95.0	14.4
Yemen	Ectopic pregnancy case management	20.4	30.0	95.0	14.4
Yemen	TT - Tetanus toxoid vaccination	93.2	95.0	95.0	65.9
Yemen	IPTp	0.0	0.0	95.0	0.0
Yemen	Syphilis detection and treatment	19.6	28.7	95.0	13.8
Yemen	Iron supplementation in pregnancy	7.3	10.7	95.0	5.2
Yemen	Hypertensive disorder case management	7.9	11.5	95.0	5.6
Yemen	Diabetes case management	6.1	9.0	95.0	4.3
Yemen	Malaria case management	25.4	37.3	95.0	18.0
Yemen	MgSO4 management of pre-eclampsia	15.6	22.9	95.0	11.0
Yemen	Clean birth environment	33.5	49.2	95.0	23.7
Yemen	Immediate drying and additional stimulation	37.4	54.9	95.0	26.4
Yemen	Thermal protection	40.3	59.2	95.0	28.5
Yemen	Clean cord care	39.0	57.2	95.0	27.6
Yemen	Manual removal of placenta	15.3	22.5	95.0	10.8
Yemen	Parenteral administration of anti-convulsants	29.1	42.8	95.0	20.6
Yemen	Antibiotics for preterm or prolonged PROM	30.5	44.7	95.0	21.6
Yemen	Parenteral administration of antibiotics	30.5	44.7	95.0	21.6
Yemen	Assisted vaginal delivery	10.4	15.2	95.0	7.3
Yemen	Neonatal resuscitation	22.5	33.0	95.0	15.9
Yemen	Parenteral administration of uterotonics	36.5	53.5	95.0	25.8
Yemen	Removal of retained products of conception	13.6	19.9	95.0	9.6
Yemen	Induction of labour for post-term pregnancies	0.7	1.0	95.0	0.5
Yemen	Antenatal corticosteroids for preterm labour	29.1	42.8	95.0	20.6
Yemen	Breastfeeding promotion	11.6	17.0	95.0	8.2
Yemen	KMC - Kangaroo mother care	2.0	3.0	95.0	1.4
Yemen	Oral antibiotics for neonatal sepsis	0.0	0.0	0.0	0.0
Yemen	Injectable antibiotics for neonatal sepsis	40.9	60.0	95.0	28.9
Zambia	Folic acid supplementation/fortification	3.6	5.3	95.0	2.5
Zambia	Safe abortion services	4.4	6.4	95.0	3.1
Zambia	Post abortion case management	67.0	95.0	95.0	47.4
Zambia	Ectopic pregnancy case management	67.0	95.0	95.0	47.4
Zambia	TT - Tetanus toxoid vaccination	95.0	95.0	95.0	80.0
Zambia	IPTp	95.0	95.0	95.0	76.5
Zambia	Syphilis detection and treatment	31.4	46.1	95.0	22.2
Zambia	Iron supplementation in pregnancy	78.7	95.0	95.0	55.6
Zambia	Hypertensive disorder case management	17.3	25.4	95.0	12.2
Zambia	Diabetes case management	13.4	19.7	95.0	9.5
Zambia	Malaria case management	55.9	82.0	95.0	39.5
Zambia	MgSO4 management of pre-eclampsia	34.2	50.2	95.0	24.2
Zambia	Clean birth environment	73.3	95.0	95.0	51.9

Country	Intervention	10% Increase	25% increase	95% target	2% Attrition
Zambia	Immediate drying and additional stimulation	81.9	95.0	95.0	57.9
Zambia	Thermal protection	88.4	95.0	95.0	62.5
Zambia	Clean cord care	85.3	95.0	95.0	60.3
Zambia	Manual removal of placenta	33.4	49.0	95.0	23.6
Zambia	Parenteral administration of anti-convulsants	63.9	93.8	95.0	45.2
Zambia	Antibiotics for preterm or prolonged PROM	66.8	95.0	95.0	47.2
Zambia	Parenteral administration of antibiotics	66.8	95.0	95.0	47.2
Zambia	Assisted vaginal delivery	22.6	33.2	95.0	16.0
Zambia	Neonatal resuscitation	49.1	72.1	95.0	34.7
Zambia	Parenteral administration of uterotonics	79.9	95.0	95.0	56.5
Zambia	Removal of retained products of conception	29.5	43.4	95.0	20.9
Zambia	Induction of labour for post-term pregnancies	1.6	2.3	95.0	1.1
Zambia	Antenatal corticosteroids for preterm labour	63.9	93.8	95.0	45.2
Zambia	Breastfeeding promotion	92.2	95.0	95.0	65.2
Zambia	KMC - Kangaroo mother care	4.5	6.6	95.0	3.2
Zambia	Oral antibiotics for neonatal sepsis	0.0	0.0	0.0	0.0
Zambia	Injectable antibiotics for neonatal sepsis	89.3	95.0	95.0	63.2
Zimbabwe	Folic acid supplementation/fortification	4.9	7.2	95.0	3.5
Zimbabwe	Safe abortion services	4.4	6.4	95.0	3.1
Zimbabwe	Post abortion case management	76.9	95.0	95.0	54.4
Zimbabwe	Ectopic pregnancy case management	76.9	95.0	95.0	54.4
Zimbabwe	TT - Tetanus toxoid vaccination	95.0	95.0	95.0	81.9
Zimbabwe	IPTp	16.1	23.6	95.0	11.4
Zimbabwe	Syphilis detection and treatment	95.0	95.0	95.0	73.2
Zimbabwe	Iron supplementation in pregnancy	52.8	77.5	95.0	37.4
Zimbabwe	Hypertensive disorder case management	39.5	58.0	95.0	28.0
Zimbabwe	Diabetes case management	38.3	56.3	95.0	27.1
Zimbabwe	Malaria case management	94.2	95.0	95.0	66.6
Zimbabwe	MgSO4 management of pre-eclampsia	95.0	95.0	95.0	67.7
Zimbabwe	Clean birth environment	81.1	95.0	95.0	57.3
Zimbabwe	Immediate drying and additional stimulation	91.4	95.0	95.0	64.7
Zimbabwe	Thermal protection	95.0	95.0	95.0	72.5
Zimbabwe	Clean cord care	90.5	95.0	95.0	64.0
Zimbabwe	Manual removal of placenta	10.1	14.8	95.0	7.2
Zimbabwe	Parenteral administration of anti-convulsants	95.0	95.0	95.0	72.3
Zimbabwe	Antibiotics for preterm or prolonged PROM	55.0	80.7	95.0	38.9
Zimbabwe	Parenteral administration of antibiotics	55.0	80.7	95.0	38.9
Zimbabwe	Assisted vaginal delivery	21.6	31.6	95.0	15.2
Zimbabwe	Neonatal resuscitation	84.4	95.0	95.0	59.7
Zimbabwe	Parenteral administration of uterotonics	95.0	95.0	95.0	69.6
Zimbabwe	Removal of retained products of conception	20.1	29.5	95.0	14.2
Zimbabwe	Induction of labour for post-term pregnancies	1.9	2.7	95.0	1.3
Zimbabwe	Antenatal corticosteroids for preterm labour	95.0	95.0	95.0	72.3
Zimbabwe	Breastfeeding promotion	55.0	80.7	95.0	38.9
Zimbabwe	KMC - Kangaroo mother care	5.1	7.5	95.0	3.6
Zimbabwe	Oral antibiotics for neonatal sepsis	0.0	0.0	0.0	0.0
Zimbabwe	Injectable antibiotics for neonatal sepsis	95.0	95.0	95.0	72.5

D Lives Saved Tool – methodological differences relative to 2014

Although the Lives Saved Tool (LiST) model was used in 2014 for a similar analysis, the software has undergone extensive revision in order to improve the quality of the results and refine its ability to model health impacts on women, children, and stillbirths. Many of the updated model parameters can be seen clearly in the LiST visualizer (listvisualizer.org).

Briefly, the stillbirth model was revised in 2016 and the description of how it was changed is available in Blencowe et al.[d] The most notable changes included the removal of periconceptual folic acid and fetal growth restriction detection and management, both of which were included in the Homer et al analysis.[e]

The antenatal care section was revised in 2017 as described in two Kanyangarara articles.[f] [g] Earlier versions of the software assumed a standard relationship between antenatal care coverage and the specific health interventions which could be delivered during antenatal care. In other words, a higher level of coverage (four antenatal care visits) was assumed to translate into a higher level of coverage of interventions being delivered to women. The Kanyangarara analyses of Service Provision Assessments and DHIS/MICS surveys suggested a more refined relationship between indicators of health care quality and the receipt of services during antenatal care. The resultant changes in assumed coverage at baseline are likely to affect the potential impact of specific health interventions.

In late 2019, the childbirth section of LiST was also revised using a similar methodology as used for the antenatal care revisions. The overall analysis was published by Kanyangarara[h] and further detailed in a technical note which will become available later in 2020 on the Lives Saved Tool website (www.livessavedtool.org). Earlier iterations of LiST had a formal linkage between facility delivery and basic emergency obstetric care and comprehensive obstetric care, similar to that used for antenatal care coverage. Thus, higher levels of facility delivery were assumed to equate to higher levels of coverage of interventions which would be part of comprehensive care. This relationship was dropped in preference of a direct link between facility delivery and specific health interventions, based on the Kanyangarara work. One critical difference can be described with the intervention which was previously called 'skilled attendance at birth', which has now been reorganized into several discrete interventions. One of these discrete interventions is called 'assisted vaginal delivery'. Although they are strongly related in terms of effectiveness impacts, the average coverage levels are dramatically different – dropping from the range of 50-80% to the range of 14-23%, in the 2014 and current analyses, respectively.

The nutrition interventions within LiST were also revised extensively in 2017, based on a series of articles in the *Journal of Nutrition*.[i]

Thus, although many of the specific interventions and linkages between interventions and causes of death are similar in the current analysis to that used in 2014, there are also notable differences which mean that the current results may differ from the earlier analysis. For each analysis, the best available data was used – the standard global sources. This should not be taken as a criticism of the 2014 results, but a recognition that the LiST tool is not a static tool, but it continually being improved with better and more up-to-date data (surveys, analyses, etc.), assumptions, and estimates.

---

[d] Blencowe H, Chou VB, Lawn JE, Bhutta ZA. Modelling stillbirth mortality reduction with the Lives Saved Tool. *BMC Public Health* 2017; 17(Suppl 4): 784.
[e] Homer CSE, Friberg IK, Dias MAB, *et al.* The projected effect of scaling up midwifery. *Lancet* 2014; **384**: 1146–57
[f] Kanyangarara M, Munos MK, Walker N. Quality of antenatal care service provision in health facilities across sub-Saharan Africa: evidence from nationally representative health facility assessments. *J Glob Health* 2017; 7(2): 021101.
[g] Kanyangarara M, Chou VB. Linking household surveys and health facility assessments to estimate intervention coverage for the Lives Saved Tool (LiST). *BMC Public Health* 2017; 17(Suppl 4): 780.
[h] Kanyangarara M, Chou VB, Creanga AA, Walker N. Linking household and health facility surveys to assess obstetric service availability, readiness and coverage: evidence from 17 low- and middle-income countries. *J Glob Health* 2018; 8(1): 010603.
[i] Walker N, Clermont A. Nutrition modelling in the Lives Saved Tool (LiST). *J Nutr* 2017; 147(11).
